# The differentially mitochondrial proteomic dataset in human ovarian cancer relative to control tissues

**DOI:** 10.1016/j.dib.2018.08.028

**Published:** 2018-08-14

**Authors:** Xianquan Zhan, Tian Zhou, Na Li, Huanni Li

**Affiliations:** a Key Laboratory of Cancer Proteomics of Chinese Ministry of Health, Xiangya Hospital, Central South University, 87 Xiangya Road, Changsha, Hunan 410008, PR China; b Hunan Engineering Laboratory for Structural Biology and Drug Design, Xiangya Hospital, Central South University, 87 Xiangya Road, Changsha, Hunan 410008, PR China; c State Local Joint Engineering Laboratory for Anticancer Drugs, Xiangya Hospital, Central South University, 87 Xiangya Road, Changsha, Hunan 410008, PR China; d The Laboratory of Medical Genetics, Central South University, 88 Xiangya Road, Changsha, Hunan 410008, PR China; e Department of Obstetrics and Gynecology, Xiangya Hospital, Central South University, Changsha, Hunan 410008, PR China

## Abstract

This data article presents a differentially mitochondrial proteomic dataset in human ovarian cancer tissues relative to control tissues. The mitochondrial samples were prepared from human ovarian cancer and control ovary tissues, and were digested with trypsin. The tryptic peptides from ovarian cancer and control mitochondrial samples were labeled by isobaric tags for relative and absolute quantification (iTRAQ) reagents, followed by strong-cation exchange (SCX) chromatography, liquid chromatography (LC)-tandem mass spectrometry (MS/MS), and bioinformatic analysis. This data article is related to a published article (Li et al., 2018) [1].

**Specifications Table**

**Table:** 

Subject area	Biological medicine
More specific subject area	Mitochondrial proteomics
Type of data	Table
How data was acquired	Isobaric tags for relative and absolute quantification (iTRAQ)-labeled samples were mixed and fractionated with strong-cation exchange (SCX) chromatography, followed by a 60-min liquid chromatography-tandem mass spectrometry (LC-MS/MS) analysis on a Q Exactive mass spectrometer (Thermo Scientific) that was coupled to Easy nLC (Proxeon Biosystems, now Thermo Fisher Scientific) for each fraction.
Data format	PDF file (Raw)
Experimental factors	Mitochondria were separated and purified from human ovarian cancer tissues and control ovary tissues.
Experimental features	A. Mitochondrial samples were digested with trypsin. B. The tryptic peptides were labeled with iTRAQ reagents. C. The iTRAQ labeled samples were mixed equally. D. The mixed iTRAQ samples were fractionated with SCX. E. Each fractionation was analyzed with LC-MS/MS. F. Database search and data analysis to determine each differentially expressed protein.
Data source location	Xiangya Hospital, Central South University, Hunan 410008 PR China
Data accessibility	Data is within this article, provided as supplementary file.
Related research article	N. Li, X.H. Zhan, X. Zhan. The lncRNA SNHG3 regulates energy metabolism of ovarian cancer by an analysis of mitochondrial proteomes. Gynecologic Oncology, 2018, 150 (2): 343–354. 10.1016/j.ygyno.2018.06.013[1]

**Value of the data**

• Mitochondria were separated and purified from ovarian cancer tissues and control ovary tissues, with high purity.
• Dataset includes 1198 differentially expressed proteins identified from ovarian cancer and control mitochondrial samples.
• Relative abundance (ratio of cancer to control), statistically significant *p*-value, peptide spectrum matches (PSMs), and database accession number might be useful for other researchers to select the method of choice according to the target of interests.

## 1 Data

Differentially expressed proteins (DEPs) identified between ovarian cancer and control mitochondrial samples are reported, with the corresponding PSMs, relative abundance (ratio of cancer to control), statistically significant *p*-value, and database accession number. Data are provided as supplementary file (Supplementary Table 1).

## 2 Experimental design, materials and methods

### 2.1 Sample preparation

Ovarian tissue samples, which include seven ovarian cancers and eleven control ovaries with benign gynecologic diseases, were collected from Department of Gynecology, Xiangya Hospital, which was approved by the Xiangya Hospital Medical Ethics Committee of Central South University, China. Mitochondria were prepared, their proteins were extracted, according to the protocols [1], [2], [3].

### 2.2 iTRAQ labeling and SCX-LC-MS/MS analysis

iTRAQ-labeling technology integrated with SCX-LC-MS/MS analysis [4], [5] were performed to identify DEPs in extracted mitochondrial protein samples from ovarian cancers and controls. In brief, mitochondrial proteins were digested with trypsin, and the tryptic peptides were labeled with the iTRAQ reagents according to the manufacturer׳s instructions (Applied Biosystems). The iTRAQ-labeled peptides were equally pooled and fractionated by SCX. Each collected fractionation was subjected to a 60-min LC-MS/MS analysis with a Q Exactive mass spectrometer (Thermo Scientific) equipped with Easy nLC (Proxeon Biosystems, now Thermo Fisher Scientific). MS/MS data were used to identify proteins with MASCOT engine (Matrix Science, London, UK; version 2.2) embedded into Proteome Discoverer 1.4. Mitochondrial DEPs were determined with the intensity difference of iTRAQ reporter ions [1].

### 2.3 Bioinformatic analysis

DAVID Bioinformatics Resources 6.7 (https://david.abcc.ncifcrf.gov/home.jsp) was used to analyze the mitochondrial DEPs, including biological process, cellular compartment, molecular function, and KEGG pathway enrichments [6].
