# Supplementary material for: The differentially mitochondrial proteomic dataset in human ovarian cancer relative to control tissues
Source: Data Brief. 2018 Aug 14;20:459–62. doi: 10.1016/j.dib.2018.08.028 (PMC6123609; doi:10.1016/j.dib.2018.08.028)
Supplement: Supplementary file 2 — Supplementary material [file mmc2.pdf]

Supplementary table 1. Differentially expressed proteins between epithelial ovarian cancer mitochondria relative to controls

Accession No.	Protein	Coverage (%)	Unique Peptides	PSMs	MW [kDa]	calc. pI	Ratio (T/N)	p value (*t* test)
P02458	Collagen alpha-1(II) chain OS=Homo sapiens GN=COL2A1 PE=1 SV=3 - [CO2A1_HUMAN]	3.03	3	3	141.70	6.9	0.17	7.01E-04
A1YBP2	Nanospan OS=Homo sapiens GN=NSPN PE=2 SV=1 - [A1YBP2_HUMAN]	8.08	1	1	10.48	5.8	0.17	6.84E-06
H0Y686	SUN domain-containing protein 2 (Fragment) OS=Homo sapiens GN=SUN2 PE=1 SV=1 - [H0Y686_HUMAN]	7.69	1	3	16.60	10.3	0.17	2.02E-04
H0YEH2	Pumilio homolog 1 (Fragment) OS=Homo sapiens GN=PUM1 PE=1 SV=1 - [H0YEH2_HUMAN]	0.62	1	1	120.15	6.8	0.17	9.26E-04
L8B4J3	Ubiquitin C OS=Homo sapiens GN=UbC PE=4 SV=1 - [L8B4J3_HUMAN]	68.32	1	39	76.95	7.7	0.18	3.35E-04
Q702N8	Xin actin-binding repeat-containing protein 1 OS=Homo sapiens GN=XIRP1 PE=1 SV=1 - [XIRP1_HUMAN]	0.38	1	1	198.44	6.2	0.20	3.11E-04
Q96KJ9	Cytochrome c oxidase subunit 4 isoform 2, mitochondrial OS=Homo sapiens GN=COX4I2 PE=1 SV=2 - [COX42_HUMAN]	5.85	1	1	20.00	9.6	0.21	6.28E-04
Q8IUE6	Histone H2A type 2-B OS=Homo sapiens GN=HIST2H2AB PE=1 SV=3 - [H2A2B_HUMAN]	23.08	1	32	13.99	10.9	0.22	5.61E-05

Q03692	Collagen alpha-1(X) chain OS=Homo sapiens GN=COL10A1 PE=1 SV=2 - [COAA1_HUMAN]	1.91	1	1	66.12	9.7	0.24	2.71E-04
D3DPH5	Collagen, type V, alpha 2, isoform CRA_b OS=Homo sapiens GN=COL5A2 PE=4 SV=1 - [D3DPH5_HUMAN]	2.14	2	2	86.03	6.1	0.24	3.59E-03
K7ELV1	Mitogen-activated protein kinase 4 OS=Homo sapiens GN=MAPK4 PE=1 SV=1 - [K7ELV1_HUMAN]	5.58	1	2	26.17	8.7	0.24	1.85E-03
P0C0S5	Histone H2A.Z OS=Homo sapiens GN=H2AFZ PE=1 SV=2 - [H2AZ_HUMAN]	31.25	2	22	13.54	10.6	0.25	8.19E-04
Q59F15	COL4A1 protein variant (Fragment) OS=Homo sapiens PE=2 SV=1 - [Q59F15_HUMAN]	5.55	3	6	82.93	8.7	0.26	2.88E-05
P13591	Neural cell adhesion molecule 1 OS=Homo sapiens GN=NCAM1 PE=1 SV=3 - [NCAM1_HUMAN]	14.92	1	23	94.52	4.9	0.26	8.31E-04
E7ESX4	S-arrestin (Fragment) OS=Homo sapiens GN=SAG PE=1 SV=1 - [E7ESX4_HUMAN]	7.69	1	1	18.67	6.6	0.27	1.43E-03
P35555	Fibrillin-1 OS=Homo sapiens GN=FBN1 PE=1 SV=3 - [FBN1_HUMAN]	30.02	67	200	312.02	4.9	0.27	1.12E-05
P02743	Serum amyloid P-component OS=Homo sapiens GN=APCS PE=1 SV=2 - [SAMP_HUMAN]	33.18	8	39	25.37	6.5	0.29	1.99E-04
A0A087WTA8	Collagen alpha-2(I) chain OS=Homo sapiens GN=COL1A2 PE=1 SV=1 - [A0A087WTA8_HUMAN]	13.27	15	55	129.07	9.0	0.29	1.84E-03
B4DWC1	cDNA FLJ51345, highly similar to Histamine N-methyltransferase (EC 2.1.1.8) OS=Homo sapiens PE=2 SV=1 - [B4DWC1_HUMAN]	5.59	1	1	18.42	6.8	0.29	6.83E-04

Q9NT22	EMILIN-3 OS=Homo sapiens GN=EMILIN3 PE=2 SV=2 - [EMIL3_HUMAN]	4.83	3	3	82.60	7.7	0.30	2.83E-04
P02461	Collagen alpha-1(III) chain OS=Homo sapiens GN=COL3A1 PE=1 SV=4 - [CO3A1_HUMAN]	6.41	9	21	138.48	6.6	0.30	6.85E-04
F2WW55	Catechol-O-methyltransferase (Fragment) OS=Homo sapiens GN=COMT PE=4 SV=1 - [F2WW55_HUMAN]	29.63	1	1	3.05	6.5	0.30	1.47E-03
P02452	Collagen alpha-1(I) chain OS=Homo sapiens GN=COL1A1 PE=1 SV=5 - [CO1A1_HUMAN]	14.96	7	87	138.86	5.8	0.30	8.28E-05
P50150	Guanine nucleotide-binding protein G(I)/G(S)/G(O) subunit gamma-4 OS=Homo sapiens GN=GNG4 PE=1 SV=1 - [GBG4_HUMAN]	32	2	3	8.38	7.1	0.30	1.39E-04
B4DE51	cDNA FLJ53714, highly similar to Mas-related G-protein coupled receptor memberF OS=Homo sapiens PE=2 SV=1 - [B4DE51_HUMAN]	6.98	2	2	34.90	8.3	0.30	1.17E-04
A0A024R2C4	B-cell CLL/lymphoma 2, isoform CRA_b OS=Homo sapiens GN=BCL2 PE=4 SV=1 - [A0A024R2C4_HUMAN]	4.88	1	1	22.32	7.0	0.31	3.30E-05
P06899	Histone H2B type 1-J OS=Homo sapiens GN=HIST1H2BJ PE=1 SV=3 - [H2B1J_HUMAN]	57.94	2	116	13.90	10.3	0.31	9.11E-04
O75937	DnaJ homolog subfamily C member 8 OS=Homo sapiens GN=DNAJC8 PE=1 SV=2 - [DNJC8_HUMAN]	6.32	1	1	29.82	9.1	0.31	1.78E-03
P02760	Protein AMBP OS=Homo sapiens GN=AMBP PE=1 SV=1 - [AMBP_HUMAN]	14.49	6	9	38.97	6.3	0.31	5.45E-05
Q6VY07	Phosphofurin acidic cluster sorting protein 1 OS=Homo sapiens GN=PACS1 PE=1 SV=2 - [PACS1_HUMAN]	2.39	2	2	104.83	7.7	0.31	1.53E-03

I3L2H6	3 beta-hydroxysteroid dehydrogenase type 7 (Fragment) OS=Homo sapiens GN=HSD3B7 PE=1 SV=1 - [I3L2H6_HUMAN]	6.25	1	1	19.21	7.0	0.31	8.57E-04
Q8IZQ1	WD repeat and FYVE domain-containing protein 3 OS=Homo sapiens GN=WDFY3 PE=1 SV=2 - [WDFY3_HUMAN]	0.77	1	3	395.01	6.8	0.31	2.06E-03
B2RBH2	cDNA, FLJ95508, highly similar to Homo sapiens 5'-nucleotidase, ecto (CD73) (NT5E), mRNA OS=Homo sapiens PE=2 SV=1 - [B2RBH2_HUMAN]	33.8	13	26	63.36	7.0	0.32	9.67E-04
H3BU16	Hematological and neurological-expressed 1-like protein (Fragment) OS=Homo sapiens GN=HN1L PE=1 SV=1 - [H3BU16_HUMAN]	8.38	1	1	17.59	9.1	0.32	4.71E-04
B4E1T8	cDNA FLJ51452, highly similar to Lysyl oxidase homolog 1 (EC1.4.3.-) OS=Homo sapiens PE=2 SV=1 - [B4E1T8_HUMAN]	5.28	2	2	48.16	6.5	0.32	1.76E-04
L0R4T3	Histone H2B OS=Homo sapiens GN=ABCF2 PE=3 SV=1 - [L0R4T3_HUMAN]	44.78	2	49	7.30	8.9	0.32	2.67E-03
P0C842	Putative uncharacterized protein encoded by LINC00614 OS=Homo sapiens GN=LINC00614 PE=5 SV=1 - [CJ052_HUMAN]	14.88	1	1	13.54	4.6	0.32	1.92E-03
H7BYY1	Tropomyosin 1 (Alpha), isoform CRA_m OS=Homo sapiens GN=TPM1 PE=1 SV=1 - [H7BYY1_HUMAN]	37.5	1	29	28.73	4.8	0.32	1.72E-04
Q9HA11	Histone H2A OS=Homo sapiens PE=2 SV=1 - [Q9HA11_HUMAN]	20.36	3	11	29.88	10.1	0.32	6.11E-04
P07305	Histone H1.0 OS=Homo sapiens GN=H1F0 PE=1 SV=3 - [H10_HUMAN]	20.62	4	22	20.85	10.8	0.32	4.06E-04
H3BTP0	Cysteine-rich secretory protein LCCL domain-containing 2 (Fragment) OS=Homo sapiens GN=CRISPLD2 PE=1 SV=8 - [H3BTP0_HUMAN]	9.01	1	1	12.76	5.4	0.32	3.84E-04

K7EN06	GTP-binding protein Di-Ras1 (Fragment) OS=Homo sapiens GN=DIRAS1 PE=1 SV=1 - [K7EN06_HUMAN]	12.5	1	2	15.11	6.5	0.32	4.69E-04
H0YGS3	Microfibrillar-associated protein 5 (Fragment) OS=Homo sapiens GN=MFAP5 PE=1 SV=1 - [H0YGS3_HUMAN]	29.11	2	3	9.44	9.5	0.32	1.27E-03
Q07507	Dermatopontin OS=Homo sapiens GN=DPT PE=1 SV=2 - [DERM_HUMAN]	9.45	2	2	23.99	4.8	0.32	5.73E-04
P05108	Cholesterol side-chain cleavage enzyme, mitochondrial OS=Homo sapiens GN=CYP11A1 PE=1 SV=2 - [CP11A_HUMAN]	38.58	18	69	60.06	8.8	0.32	8.26E-04
P16104	Histone H2AX OS=Homo sapiens GN=H2AFX PE=1 SV=2 - [H2AX_HUMAN]	21.68	1	35	15.14	10.7	0.33	1.15E-04
C9J6H1	Myelin basic protein (Fragment) OS=Homo sapiens GN=MBP PE=1 SV=1 - [C9J6H1_HUMAN]	7.92	1	1	11.09	10.3	0.33	7.08E-03
Q9NZN4	EH domain-containing protein 2 OS=Homo sapiens GN=EHD2 PE=1 SV=2 - [EHD2_HUMAN]	43.83	19	43	61.12	6.5	0.33	2.35E-04
P27658	Collagen alpha-1(VIII) chain OS=Homo sapiens GN=COL8A1 PE=1 SV=2 - [CO8A1_HUMAN]	4.84	3	8	73.32	9.6	0.33	9.61E-04
P07585	Decorin OS=Homo sapiens GN=DCN PE=1 SV=1 - [PGS2_HUMAN]	17.27	7	17	39.72	8.5	0.33	3.58E-04
K7EMY9	Cold-inducible RNA-binding protein OS=Homo sapiens GN=CIRBP PE=1 SV=1 - [K7EMY9_HUMAN]	14.47	2	2	17.04	9.9	0.33	2.73E-04
Q8TDB4	Protein MGARP OS=Homo sapiens GN=MGARP PE=1 SV=1 - [HUMMR_HUMAN]	37.5	5	10	25.37	4.4	0.33	8.37E-04

Q92522	Histone H1x OS=Homo sapiens GN=H1FX PE=1 SV=1 - [H1X_HUMAN]	24.41	5	11	22.47	10.8	0.34	7.07E-04
P10412	Histone H1.4 OS=Homo sapiens GN=HIST1H1E PE=1 SV=2 - [H14_HUMAN]	30.14	1	57	21.85	11.0	0.34	2.51E-04
Q6GPG7	LPAR1 protein OS=Homo sapiens GN=LPAR1 PE=1 SV=1 - [Q6GPG7_HUMAN]	3.76	1	1	39.24	8.5	0.34	3.55E-04
P02768	Serum albumin OS=Homo sapiens GN=ALB PE=1 SV=2 - [ALBU_HUMAN]	80.3	1	1066	69.32	6.3	0.34	6.04E-04
Q8N9M5	Transmembrane protein 102 OS=Homo sapiens GN=TMEM102 PE=1 SV=1 - [TM102_HUMAN]	1.57	1	1	54.14	5.9	0.34	2.28E-04
P51888	Prolargin OS=Homo sapiens GN=PRELP PE=1 SV=1 - [PRELP_HUMAN]	39.27	13	25	43.78	9.4	0.34	8.31E-04
Q92629	Delta-sarcoglycan OS=Homo sapiens GN=SGCD PE=1 SV=2 - [SGCD_HUMAN]	19.38	5	9	32.05	9.1	0.34	2.98E-04
Q03252	Lamin-B2 OS=Homo sapiens GN=LMNB2 PE=1 SV=4 - [LMNB2_HUMAN]	32.74	21	39	69.91	5.6	0.34	6.10E-04
Q9BX67	Junctional adhesion molecule C OS=Homo sapiens GN=JAM3 PE=1 SV=1 - [JAM3_HUMAN]	10	3	5	35.00	7.6	0.34	1.04E-03
B7Z6U8	cDNA FLJ53665, highly similar to Four and a half LIM domains protein 1 OS=Homo sapiens PE=2 SV=1 - [B7Z6U8_HUMAN]	43.85	10	20	29.48	8.3	0.34	1.12E-03
P48061	Stromal cell-derived factor 1 OS=Homo sapiens GN=CXCL12 PE=1 SV=1 - [SDF1_HUMAN]	17.2	2	4	10.66	9.9	0.35	5.10E-03

Q8IYS1	Peptidase M20 domain-containing protein 2 OS=Homo sapiens GN=PM20D2 PE=1 SV=2 - [P20D2_HUMAN]	2.52	1	1	47.75	5.8	0.35	5.12E-04
A8K2W3	cDNA FLJ78516 OS=Homo sapiens PE=2 SV=1 - [A8K2W3_HUMAN]	18.12	8	9	47.07	5.2	0.35	1.40E-03
Q5CZB5	Putative uncharacterized protein DKFZp686M0430 OS=Homo sapiens GN=DKFZp686M0430 PE=2 SV=1 - [Q5CZB5_HUMAN]	21.69	1	26	125.01	4.5	0.35	1.42E-03
O00479	High mobility group nucleosome-binding domain-containing protein 4 OS=Homo sapiens GN=HMGN4 PE=1 SV=3 - [HMGN4_HUMAN]	7.78	1	1	9.53	10.5	0.35	1.21E-03
B4DW52	cDNA FLJ55253, highly similar to Actin, cytoplasmic 1 OS=Homo sapiens PE=2 SV=1 - [B4DW52_HUMAN]	69.45	1	549	38.61	5.4	0.36	5.52E-04
D3DTX7	Collagen, type I, alpha 1, isoform CRA_a OS=Homo sapiens GN=COL1A1 PE=4 SV=1 - [D3DTX7_HUMAN]	18.53	2	61	84.69	6.2	0.36	5.47E-05
P55083	Microfibril-associated glycoprotein 4 OS=Homo sapiens GN=MFAP4 PE=1 SV=2 - [MFAP4_HUMAN]	14.51	4	23	28.63	5.6	0.36	5.61E-04
B3KRY5	cDNA FLJ35087 fis, clone PLACE6005546, highly similar to Polymerase I and transcript release factor OS=Homo sapiens PE=2 SV=1 - [B3KRY5_HUMAN]	22.03	7	18	39.02	6.5	0.36	8.42E-05
O00168	Phospholemman OS=Homo sapiens GN=FXYD1 PE=1 SV=2 - [PLM_HUMAN]	13.04	1	2	10.43	9.1	0.36	8.86E-03
P25101	Endothelin-1 receptor OS=Homo sapiens GN=EDNRA PE=1 SV=1 - [EDNRA_HUMAN]	1.64	1	1	48.69	8.4	0.36	2.74E-04
B4E2J2	cDNA FLJ51755, highly similar to Glutathione S-transferase Mu 3 (EC 2.5.1.18) OS=Homo sapiens PE=2 SV=1 - [B4E2J2_HUMAN]	53.68	1	26	16.12	5.2	0.37	6.56E-04

B3KY43	cDNA FLJ46798 fis, clone TRACH3031660, highly similar to cAMP-dependent protein kinase type II-beta regulatory subunit OS=Homo sapiens PE=2 SV=1 - [B3KY43_HUMAN]	38.27	11	22	44.84	5.2	0.37	1.35E-04
E7ENL6	Collagen alpha-3(VI) chain OS=Homo sapiens GN=COL6A3 PE=1 SV=2 - [E7ENL6_HUMAN]	31.1	1	165	277.95	8.2	0.37	4.07E-04
P21266	Glutathione S-transferase Mu 3 OS=Homo sapiens GN=GSTM3 PE=1 SV=3 - [GSTM3_HUMAN]	52.44	4	43	26.54	5.5	0.37	5.16E-04
Q9UBX5	Fibulin-5 OS=Homo sapiens GN=FBLN5 PE=1 SV=1 - [FBLN5_HUMAN]	19.42	8	15	50.15	4.7	0.37	2.40E-03
P08913	Alpha-2A adrenergic receptor OS=Homo sapiens GN=ADRA2A PE=1 SV=3 - [ADA2A_HUMAN]	2.67	1	1	48.93	9.7	0.37	1.30E-03
Q16344	Protein-tyrosine phosphatase (Fragment) OS=Homo sapiens GN=BPTP-3 PE=2 SV=3 - [Q16344_HUMAN]	8.79	1	1	10.56	6.3	0.37	1.06E-03
E7EWB4	Dihydropyrimidinase-related protein 5 (Fragment) OS=Homo sapiens GN=DPYSL5 PE=1 SV=8 - [E7EWB4_HUMAN]	3.7	1	1	20.75	6.4	0.37	8.07E-04
Q7Z351	Putative uncharacterized protein DKFZp686N02209 OS=Homo sapiens GN=DKFZp686N02209 PE=2 SV=1 - [Q7Z351_HUMAN]	40.25	1	155	52.82	8.5	0.37	2.12E-03
Q8NGA1	Olfactory receptor 1M1 OS=Homo sapiens GN=OR1M1 PE=2 SV=1 - [OR1M1_HUMAN]	2.24	1	8	34.82	8.9	0.38	1.28E-03
A1L0S7	TNS1 protein (Fragment) OS=Homo sapiens GN=TNS1 PE=2 SV=2 - [A1L0S7_HUMAN]	9.05	11	13	188.92	7.9	0.38	5.60E-04
A0A059T3A1	NADH-ubiquinone oxidoreductase chain 2 OS=Homo sapiens GN=ND2 PE=3 SV=1 - [A0A059T3A1_HUMAN]	4.61	1	2	38.93	9.8	0.38	6.03E-04

Q86VY4	Testis-specific Y-encoded-like protein 5 OS=Homo sapiens GN=TSPYL5 PE=1 SV=2 - [TSYL5_HUMAN]	2.4	1	1	45.12	9.5	0.38	5.64E-03
A5Z217	Mutant desmin OS=Homo sapiens PE=2 SV=1 - [A5Z217_HUMAN]	45.96	17	38	53.56	5.3	0.38	3.25E-04
A0A024F8S6	HLA-DRB5 protein (Fragment) OS=Homo sapiens GN=HLA-DRB5 PE=3 SV=1 - [A0A024F8S6_HUMAN]	42.48	1	23	25.73	6.5	0.38	2.40E-04
P80723	Brain acid soluble protein 1 OS=Homo sapiens GN=BASP1 PE=1 SV=2 - [BASP1_HUMAN]	78.41	12	22	22.68	4.6	0.38	7.48E-05
Q5TEC6	Histone H3 OS=Homo sapiens GN=HIST2H3PS2 PE=1 SV=1 - [Q5TEC6_HUMAN]	26.47	2	18	15.42	11.3	0.38	1.14E-02
A0A096WB60	NADH-ubiquinone oxidoreductase chain 5 OS=Homo sapiens GN=ND5 PE=3 SV=1 - [A0A096WB60_HUMAN]	5.14	1	6	67.01	9.0	0.38	3.34E-04
I3L102	Ethanolamine-phosphate cytidylyltransferase OS=Homo sapiens GN=PCYT2 PE=1 SV=1 - [I3L102_HUMAN]	31.82	1	2	4.59	10.0	0.39	4.67E-03
A0A024RAV4	Cold shock domain protein A, isoform CRA_b OS=Homo sapiens GN=CSDA PE=4 SV=1 - [A0A024RAV4_HUMAN]	8.58	1	3	31.93	9.7	0.39	1.90E-03
B2RBF8	cDNA, FLJ95486, highly similar to Homo sapiens phospholipid scramblase 3 (PLSCR3), mRNA OS=Homo sapiens PE=2 SV=1 - [B2RBF8_HUMAN]	6.44	1	1	31.64	6.4	0.39	6.63E-03
A0A024RCH0	Transcription elongation factor A (SII)-like 6, isoform CRA_a OS=Homo sapiens GN=TCEAL6 PE=4 SV=1 - [A0A024RCH0_HUMAN]	4.92	1	1	20.33	4.8	0.39	2.20E-04
B4DVP5	cDNA FLJ60908, highly similar to Homo sapiens fibroblast growth factor receptor 4 (FGFR4), transcript variant 2, mRNA OS=Homo sapiens PE=2 SV=1 - [B4DVP5_HUMAN]	14.03	7	42	80.40	6.3	0.39	1.76E-05

P08572	Collagen alpha-2(IV) chain OS=Homo sapiens GN=COL4A2 PE=1 SV=4 - [CO4A2_HUMAN]	6.43	7	18	167.45	8.7	0.39	4.18E-03
Q9NQC0	Extraneuronal monoamine transporter (EMT) (Fragment) OS=Homo sapiens GN=SLC22A3 PE=4 SV=1 - [Q9NQC0_HUMAN]	6.99	1	1	15.75	7.7	0.39	1.78E-03
Q13268	Dehydrogenase/reductase SDR family member 2, mitochondrial OS=Homo sapiens GN=DHRS2 PE=1 SV=4 - [DHRS2_HUMAN]	33.93	7	18	29.91	9.0	0.39	1.43E-03
B4DH22	cDNA FLJ59884, highly similar to Secernin-1 OS=Homo sapiens PE=2 SV=1 - [B4DH22_HUMAN]	5.05	1	1	24.78	4.7	0.39	3.53E-03
B0YJ73	Ras suppressor protein 1 variant 5 OS=Homo sapiens GN=RSU1 PE=4 SV=1 - [B0YJ73_HUMAN]	11.35	3	3	25.72	7.2	0.39	1.66E-04
F5HB16	cDNA FLJ51682, highly similar to Alcohol dehydrogenase 1B (EC 1.1.1.1) OS=Homo sapiens PE=2 SV=1 - [F5HB16_HUMAN]	41.99	15	53	38.32	8.0	0.40	2.01E-04
P12109	Collagen alpha-1(VI) chain OS=Homo sapiens GN=COL6A1 PE=1 SV=3 - [CO6A1_HUMAN]	32.78	27	57	108.46	5.4	0.40	1.54E-03
Q13813	Spectrin alpha chain, non-erythrocytic 1 OS=Homo sapiens GN=SPTAN1 PE=1 SV=3 - [SPTN1_HUMAN]	41.95	1	179	284.36	5.4	0.40	5.44E-04
A6NMZ7	Collagen alpha-6(VI) chain OS=Homo sapiens GN=COL6A6 PE=1 SV=2 - [CO6A6_HUMAN]	0.71	1	2	247.02	6.9	0.40	2.11E-03
P35749	Myosin-11 OS=Homo sapiens GN=MYH11 PE=1 SV=3 - [MYH11_HUMAN]	50.61	80	389	227.20	5.5	0.40	7.12E-04
A8VPY0	Collagen alpha 6 type IV (Fragment) OS=Homo sapiens PE=2 SV=1 - [A8VPY0_HUMAN]	4.8	1	1	25.46	7.6	0.40	3.83E-04

B3KQX7	cDNA FLJ33256 fis, clone ASTRO2005557, highly similar to MAJOR PRION PROTEIN OS=Homo sapiens PE=2 SV=1 - [B3KQX7_HUMAN]	17.79	3	4	18.16	5.8	0.40	6.61E-03
L8E853	von Willebrand factor OS=Homo sapiens GN=VWF PE=4 SV=1 - [L8E853_HUMAN]	11.42	2	37	298.18	5.5	0.40	2.98E-04
P12277	Creatine kinase B-type OS=Homo sapiens GN=CKB PE=1 SV=1 - [KCRB_HUMAN]	29.4	6	10	42.62	5.6	0.40	7.78E-04
B7Z2F7	cDNA FLJ54655, highly similar to Heat shock 70 kDa protein 12A OS=Homo sapiens PE=2 SV=1 - [B7Z2F7_HUMAN]	19.76	8	17	66.37	8.3	0.40	3.09E-04
E5RJF6	Carbonic anhydrase 1 (Fragment) OS=Homo sapiens GN=CA1 PE=1 SV=1 - [E5RJF6_HUMAN]	43.68	4	15	9.40	9.6	0.41	1.28E-03
Q6UWR7	Ectonucleotide pyrophosphatase/phosphodiesterase family member 6 OS=Homo sapiens GN=ENPP6 PE=1 SV=2 - [ENPP6_HUMAN]	23.18	8	10	50.21	8.0	0.41	7.22E-04
P02545	Prelamin-A/C OS=Homo sapiens GN=LMNA PE=1 SV=1 - [LMNA_HUMAN]	46.54	29	52	74.09	7.0	0.41	1.12E-03
P27338	Amine oxidase [flavin-containing] B OS=Homo sapiens GN=MAOB PE=1 SV=3 - [AOFB_HUMAN]	43.46	18	143	58.73	7.5	0.41	2.86E-04
Q6UX72	UDP-GlcNAc:betaGal beta-1,3-N-acetylglucosaminyltransferase 9 OS=Homo sapiens GN=B3GNT9 PE=2 SV=1 - [B3GN9_HUMAN]	3.73	1	1	43.72	9.2	0.41	3.04E-04
H0YAZ3	Vinexin (Fragment) OS=Homo sapiens GN=SORBS3 PE=1 SV=1 - [H0YAZ3_HUMAN]	8.6	1	1	20.54	9.0	0.41	6.43E-04
Q16853	Membrane primary amine oxidase OS=Homo sapiens GN=AOC3 PE=1 SV=3 - [AOC3_HUMAN]	14.15	9	17	84.57	6.5	0.41	1.03E-03

B7ZKQ9	SCARB1 protein OS=Homo sapiens GN=SCARB1 PE=1 SV=1 - [B7ZKQ9_HUMAN]	21.62	9	20	53.81	7.9	0.42	6.99E-04
Q9BQJ4	Transmembrane protein 47 OS=Homo sapiens GN=TMEM47 PE=1 SV=1 - [TMM47_HUMAN]	6.08	1	1	19.98	6.7	0.42	4.69E-03
B2R8L0	cDNA, FLJ93951, highly similar to Homo sapiens hydroxy-delta-5-steroid dehydrogenase, 3 beta- andsteroid delta-isomerase 2 (HSD3B2), mRNA OS=Homo sapiens PE=2 SV=1 - [B2R8L0_HUMAN]	33.33	8	12	42.05	8.0	0.42	2.99E-04
A0A0B4J1Z1	Serine/arginine-rich-splicing factor 7 OS=Homo sapiens GN=SRSF7 PE=1 SV=1 - [A0A0B4J1Z1_HUMAN]	21.9	2	4	15.75	9.8	0.42	7.48E-04
A0A0C4DFX3	EMILIN-1 OS=Homo sapiens GN=EMILIN1 PE=1 SV=1 - [A0A0C4DFX3_HUMAN]	28.05	22	39	106.63	5.2	0.42	3.74E-04
B3KMX6	cDNA FLJ12864 fis, clone NT2RP2003604, highly similar to Alpha-catulin OS=Homo sapiens PE=2 SV=1 - [B3KMX6_HUMAN]	4.5	2	3	81.85	6.6	0.42	4.35E-03
E5RH11	Heparan-alpha-glucosaminide N-acetyltransferase OS=Homo sapiens GN=HGSNAT PE=4 SV=1 - [E5RH11_HUMAN]	8.25	1	1	10.65	8.9	0.42	4.23E-03
Q3MIR4	Cell cycle control protein 50B OS=Homo sapiens GN=TMEM30B PE=1 SV=1 - [CC50B_HUMAN]	3.99	1	1	38.92	7.9	0.42	8.38E-03
Q9UH97	Hematopoietic progenitor cell antigen CD34 (Fragment) OS=Homo sapiens GN=CD34 PE=2 SV=1 - [Q9UH97_HUMAN]	4.15	1	2	20.70	7.2	0.42	2.14E-04
A8K9G2	cDNA FLJ76366 OS=Homo sapiens PE=2 SV=1 - [A8K9G2_HUMAN]	18.41	1	35	22.05	4.9	0.42	3.87E-03
P62805	Histone H4 OS=Homo sapiens GN=HIST1H4A PE=1 SV=2 - [H4_HUMAN]	51.46	6	153	11.36	11.4	0.43	3.15E-04

Q96GP6	Scavenger receptor class F member 2 OS=Homo sapiens GN=SCARF2 PE=1 SV=4 - [SREC2_HUMAN]	1.26	1	1	92.42	8.4	0.43	7.96E-04
P11511	Aromatase OS=Homo sapiens GN=CYP19A1 PE=1 SV=3 - [CP19A_HUMAN]	18.49	8	14	57.84	7.5	0.43	3.50E-04
Q07092	Collagen alpha-1(XVI) chain OS=Homo sapiens GN=COL16A1 PE=1 SV=2 - [COGA1_HUMAN]	1.81	3	4	157.65	7.8	0.43	7.50E-04
K7EP04	Heat shock protein beta-6 OS=Homo sapiens GN=HSPB6 PE=1 SV=1 - [K7EP04_HUMAN]	24.82	3	5	14.65	8.9	0.43	4.80E-03
B4DJ89	Polymerase (RNA) II (DNA directed) polypeptide E, 25kDa, isoform CRA_c OS=Homo sapiens GN=POLR2E PE=2 SV=1 - [B4DJ89_HUMAN]	8.55	1	1	13.54	8.4	0.43	6.77E-03
O75475	PC4 and SFRS1-interacting protein OS=Homo sapiens GN=PSIP1 PE=1 SV=1 - [PSIP1_HUMAN]	1.51	1	1	60.07	9.1	0.43	1.40E-03
E7ETP7	Elastin (Fragment) OS=Homo sapiens GN=ELN PE=1 SV=1 - [E7ETP7_HUMAN]	6.46	1	1	26.16	10.3	0.43	1.02E-02
F8TAD1	Pax7-forkhead fusion protein (Fragment) OS=Homo sapiens PE=2 SV=1 - [F8TAD1_HUMAN]	1.98	1	1	44.15	9.3	0.43	2.30E-03
P51911	Calponin-1 OS=Homo sapiens GN=CNN1 PE=1 SV=2 - [CNN1_HUMAN]	41.08	9	25	33.15	9.1	0.43	2.66E-06
I3L2I2	Misshapen-like kinase 1 (Fragment) OS=Homo sapiens GN=MINK1 PE=1 SV=1 - [I3L2I2_HUMAN]	2.89	1	1	44.94	7.2	0.43	3.69E-05
P0DJ93	Small integral membrane protein 13 OS=Homo sapiens GN=SMIM13 PE=3 SV=1 - [SIM13_HUMAN]	12.09	1	1	10.34	6.7	0.44	1.12E-03

Q8TE73	Dynein heavy chain 5, axonemal OS=Homo sapiens GN=DNAH5 PE=1 SV=3 - [DYH5_HUMAN]	0.3	1	3	528.68	6.1	0.44	3.12E-03
Q59FA2	Splicing factor, arginine/serine-rich 1 (Splicing factor 2, alternate splicing factor) variant (Fragment) OS=Homo sapiens PE=2 SV=1 - [Q59FA2_HUMAN]	16.74	4	5	25.61	9.0	0.44	5.40E-04
B8ZZG1	MAGUK p55 subfamily member 6 OS=Homo sapiens GN=MPP6 PE=1 SV=1 - [B8ZZG1_HUMAN]	2.34	1	1	48.73	8.8	0.44	2.93E-03
C9JD84	Latent-transforming growth factor beta-binding protein 1 OS=Homo sapiens GN=LTBP1 PE=1 SV=1 - [C9JD84_HUMAN]	0.67	1	2	146.91	4.9	0.44	2.78E-03
P49863	Granzyme K OS=Homo sapiens GN=GZMK PE=1 SV=1 - [GRAK_HUMAN]	7.2	1	1	28.86	9.4	0.44	3.53E-03
P12110	Collagen alpha-2(VI) chain OS=Homo sapiens GN=COL6A2 PE=1 SV=4 - [CO6A2_HUMAN]	18.84	18	50	108.51	6.2	0.44	5.27E-04
K7EK07	Histone H3 (Fragment) OS=Homo sapiens GN=H3F3B PE=1 SV=1 - [K7EK07_HUMAN]	53.03	4	34	14.91	11.3	0.44	9.51E-05
P00918	Carbonic anhydrase 2 OS=Homo sapiens GN=CA2 PE=1 SV=2 - [CAH2_HUMAN]	30	6	14	29.23	7.4	0.44	3.08E-04
P00352	Retinal dehydrogenase 1 OS=Homo sapiens GN=ALDH1A1 PE=1 SV=2 - [AL1A1_HUMAN]	44.71	16	47	54.83	6.7	0.44	1.39E-04
P08779	Keratin, type I cytoskeletal 16 OS=Homo sapiens GN=KRT16 PE=1 SV=4 - [K1C16_HUMAN]	21.78	1	12	51.24	5.0	0.44	3.73E-04
Q53TN4	Cytochrome b reductase 1 OS=Homo sapiens GN=CYBRD1 PE=1 SV=1 - [CYBR1_HUMAN]	7.69	2	3	31.62	8.8	0.44	2.46E-03

M0R317	Striatin-4 (Fragment) OS=Homo sapiens GN=STRN4 PE=1 SV=1 - [M0R317_HUMAN]	5.05	1	1	20.98	6.7	0.45	7.25E-03
B4DI81	Gap junction protein OS=Homo sapiens PE=2 SV=1 - [B4DI81_HUMAN]	10.25	3	8	40.49	8.7	0.45	2.63E-03
A0A0S2Z3V1	EGF containing fibulin-like extracellular matrix protein 1 isoform 2 (Fragment) OS=Homo sapiens GN=EFEMP1 PE=2 SV=1 - [A0A0S2Z3V1_HUMAN]	22.28	7	10	45.69	5.5	0.45	3.69E-04
B4E397	cDNA FLJ55140, highly similar to SPARC-like protein 1 OS=Homo sapiens PE=2 SV=1 - [B4E397_HUMAN]	6.53	2	2	55.48	4.8	0.45	8.65E-03
O76062	Delta(14)-sterol reductase OS=Homo sapiens GN=TM7SF2 PE=2 SV=3 - [ERG24_HUMAN]	9.09	4	7	46.38	8.9	0.45	9.60E-07
Q13884	Beta-1-syntrophin OS=Homo sapiens GN=SNTB1 PE=1 SV=3 - [SNTB1_HUMAN]	5.39	1	3	58.03	8.6	0.45	3.56E-03
Q01995	Transgelin OS=Homo sapiens GN=TAGLN PE=1 SV=4 - [TAGL_HUMAN]	73.13	14	98	22.60	8.8	0.45	2.32E-04
P51884	Lumican OS=Homo sapiens GN=LUM PE=1 SV=2 - [LUM_HUMAN]	27.22	9	15	38.40	6.6	0.45	1.20E-03
Q9H1E3	Nuclear ubiquitous casein and cyclin-dependent kinase substrate 1 OS=Homo sapiens GN=NUCKS1 PE=1 SV=1 - [NUCKS_HUMAN]	3.7	1	3	27.28	5.1	0.45	7.70E-04
Q8ND94	LRRN4 C-terminal-like protein OS=Homo sapiens GN=LRRN4CL PE=2 SV=1 - [LRN4L_HUMAN]	9.24	1	1	25.25	6.5	0.45	4.36E-03
P84157	Matrix-remodeling-associated protein 7 OS=Homo sapiens GN=MXRA7 PE=1 SV=1 - [MXRA7_HUMAN]	26.96	3	6	21.45	4.3	0.45	2.58E-03

Q9BXN1	Asporin OS=Homo sapiens GN=ASPN PE=1 SV=2 - [ASPN_HUMAN]	10.53	4	9	43.39	7.1	0.45	1.91E-03
Q5JZ08	Hephaestin (Fragment) OS=Homo sapiens GN=HEPH PE=1 SV=1 - [Q5JZ08_HUMAN]	1.36	1	1	91.30	5.9	0.45	8.44E-03
P10109	Adrenodoxin, mitochondrial OS=Homo sapiens GN=FDX1 PE=1 SV=1 - [ADX_HUMAN]	22.83	5	9	19.38	5.8	0.46	2.50E-03
P09488	Glutathione S-transferase Mu 1 OS=Homo sapiens GN=GSTM1 PE=1 SV=3 - [GSTM1_HUMAN]	48.17	3	24	25.70	6.7	0.46	6.84E-04
B3KME0	cDNA FLJ10760 fis, clone NT2RP3004618, highly similar to Eukaryotic translation initiation factor 2C 1 OS=Homo sapiens PE=2 SV=1 - [B3KME0_HUMAN]	5.37	2	3	88.66	9.2	0.46	3.25E-05
Q4ZG81	Putative uncharacterized protein FLJ20701 (Fragment) OS=Homo sapiens GN=FLJ20701 PE=4 SV=1 - [Q4ZG81_HUMAN]	8.86	1	1	18.03	6.0	0.46	1.39E-03
A0A024QZE7	Transforming growth factor beta 1 induced transcript 1, isoform CRA_a OS=Homo sapiens GN=TGFB1I1 PE=4 SV=1 - [A0A024QZE7_HUMAN]	6.76	3	3	47.91	7.6	0.46	4.72E-04
B2R815	cDNA, FLJ93695, highly similar to Homo sapiens serpin peptidase inhibitor, clade A (alpha-1 antiproteinase, antitrypsin), member 4 (SERPINA4), mRNA OS=Homo sapiens PE=2 SV=1 - [B2R815_HUMAN]	2.81	1	1	48.50	7.8	0.46	4.19E-03
B4DWG8	cDNA FLJ60923, highly similar to Phosphate-regulating neutral endopeptidase (EC3.4.24.-) OS=Homo sapiens PE=2 SV=1 - [B4DWG8_HUMAN]	3.22	2	2	76.05	9.1	0.46	1.81E-03
P50440	Glycine amidinotransferase, mitochondrial OS=Homo sapiens GN=GATM PE=1 SV=1 - [GATM_HUMAN]	47.04	18	45	48.42	8.0	0.46	1.69E-03
Q96RY7	Intraflagellar transport protein 140 homolog OS=Homo sapiens GN=IFT140 PE=1 SV=1 - [IF140_HUMAN]	1.3	2	2	165.09	6.0	0.46	3.55E-04

Q05B42	SARM1 protein (Fragment) OS=Homo sapiens GN=SARM1 PE=2 SV=1 - [Q05B42_HUMAN]	1.78	1	1	67.90	6.5	0.46	1.07E-03
O75367	Core histone macro-H2A.1 OS=Homo sapiens GN=H2AFY PE=1 SV=4 - [H2AY_HUMAN]	17.74	4	22	39.59	9.8	0.47	2.73E-03
P09455	Retinol-binding protein 1 OS=Homo sapiens GN=RBP1 PE=1 SV=2 - [RET1_HUMAN]	70.37	10	34	15.84	5.1	0.47	7.06E-04
C9IY88	3-hydroxyanthranilate 3,4-dioxygenase (Fragment) OS=Homo sapiens GN=HAAO PE=1 SV=8 - [C9IY88_HUMAN]	6.25	1	1	20.44	5.2	0.47	3.94E-03
P20774	Mimecan OS=Homo sapiens GN=OGN PE=1 SV=1 - [MIME_HUMAN]	34.56	10	30	33.90	5.6	0.47	5.42E-04
P54289	Voltage-dependent calcium channel subunit alpha-2/delta-1 OS=Homo sapiens GN=CACNA2D1 PE=1 SV=3 - [CA2D1_HUMAN]	13.96	13	19	124.49	5.3	0.47	1.26E-04
Q05707	Collagen alpha-1(XIV) chain OS=Homo sapiens GN=COL14A1 PE=1 SV=3 - [COEA1_HUMAN]	29.34	41	88	193.39	5.3	0.47	1.11E-03
A7YME7	IASPP short isoform OS=Homo sapiens GN=PPP1R13L PE=2 SV=1 - [A7YME7_HUMAN]	5.65	1	1	44.08	4.9	0.47	4.27E-03
H3BRU6	Poly(rC)-binding protein 2 (Fragment) OS=Homo sapiens GN=PCBP2 PE=1 SV=1 - [H3BRU6_HUMAN]	34.55	1	13	31.69	8.4	0.47	4.23E-03
A8K987	cDNA FLJ77911, highly similar to Homo sapiens glutathione S-transferase A2 (GSTA2), mRNA OS=Homo sapiens PE=2 SV=1 - [A8K987_HUMAN]	23.42	6	22	25.66	9.0	0.47	5.43E-04
F8VSC4	Methionine aminopeptidase 2 (Fragment) OS=Homo sapiens GN=METAP2 PE=1 SV=1 - [F8VSC4_HUMAN]	14.29	1	2	17.06	6.1	0.47	2.07E-03

Q03135	Caveolin-1 OS=Homo sapiens GN=CAV1 PE=1 SV=4 - [CAV1_HUMAN]	47.19	7	34	20.46	6.0	0.47	4.14E-05
B3KQU6	cDNA PSEC0207 fis, clone HEMBA1002981, highly similar to Homo sapiens nurim (nuclear envelope membrane protein) (NRM), mRNA OS=Homo sapiens PE=2 SV=1 - [B3KQU6_HUMAN]	16.79	4	9	29.37	8.6	0.47	1.25E-04
H0Y8S4	Ubiquitin thioesterase otulin (Fragment) OS=Homo sapiens GN=OTULIN PE=1 SV=1 - [H0Y8S4_HUMAN]	9.09	1	1	7.53	8.4	0.47	7.13E-04
O43930	Putative serine/threonine-protein kinase PRKY OS=Homo sapiens GN=PRKY PE=5 SV=1 - [PRKY_HUMAN]	6.86	1	1	31.69	7.0	0.47	2.12E-05
P13671	Complement component C6 OS=Homo sapiens GN=C6 PE=1 SV=3 - [CO6_HUMAN]	4.71	1	5	104.72	6.8	0.47	1.05E-02
B4DQA3	cDNA FLJ58603, highly similar to Actin-binding LIM protein 1 OS=Homo sapiens PE=2 SV=1 - [B4DQA3_HUMAN]	3.68	1	1	43.83	9.0	0.47	7.10E-03
Q9UNW9	RNA-binding protein Nova-2 OS=Homo sapiens GN=NOVA2 PE=1 SV=1 - [NOVA2_HUMAN]	1.63	1	1	48.98	8.2	0.47	1.95E-03
O75781	Paralemmin-1 OS=Homo sapiens GN=PALM PE=1 SV=2 - [PALM_HUMAN]	4.91	2	2	42.05	5.0	0.48	2.73E-03
Q5VY30	Retinol binding protein 4, plasma, isoform CRA_b OS=Homo sapiens GN=RBP4 PE=1 SV=2 - [Q5VY30_HUMAN]	5.03	1	2	22.96	6.1	0.48	1.47E-04
Q5JXC2	Migration and invasion-inhibitory protein OS=Homo sapiens GN=MIIP PE=1 SV=3 - [MIIP_HUMAN]	3.87	1	1	42.80	8.3	0.48	6.31E-04
O43705	Platelet glycoprotein IX (Fragment) OS=Homo sapiens PE=4 SV=1 - [O43705_HUMAN]	22.03	1	1	6.43	6.9	0.48	1.30E-03

D6RDM3	Solute carrier family 41 member 3 OS=Homo sapiens GN=SLC41A3 PE=1 SV=1 - [D6RDM3_HUMAN]	27.69	1	5	6.71	5.1	0.48	4.74E-03
H0YEX9	Junctional adhesion molecule B (Fragment) OS=Homo sapiens GN=JAM2 PE=1 SV=1 - [H0YEX9_HUMAN]	13.03	3	5	29.11	8.8	0.48	8.87E-04
E9PCT3	Caveolin OS=Homo sapiens GN=CAV2 PE=1 SV=1 - [E9PCT3_HUMAN]	15.93	1	3	12.94	5.1	0.48	1.12E-02
H0Y804	Aldo-keto reductase family 1 member C1 (Fragment) OS=Homo sapiens GN=AKR1C1 PE=1 SV=1 - [H0Y804_HUMAN]	6.05	2	2	28.16	8.8	0.48	6.06E-04
K7EN70	Protein FAM198B (Fragment) OS=Homo sapiens GN=FAM198B PE=1 SV=1 - [K7EN70_HUMAN]	9.09	2	3	21.57	8.0	0.48	4.36E-03
P63096	Guanine nucleotide-binding protein G(i) subunit alpha-1 OS=Homo sapiens GN=GNAI1 PE=1 SV=2 - [GNAI1_HUMAN]	27.4	1	67	40.34	6.0	0.48	1.71E-03
F6SDV2	Tubulointerstitial nephritis antigen-like OS=Homo sapiens GN=TINAGL1 PE=1 SV=1 - [F6SDV2_HUMAN]	17.13	5	6	40.84	7.0	0.48	1.22E-03
Q6ZR64	HBV PreS1-transactivated protein 1 OS=Homo sapiens GN=MXRA7 PE=1 SV=1 - [Q6ZR64_HUMAN]	16.9	1	6	23.40	6.5	0.48	3.55E-03
A0A087WTF6	Neural cell adhesion molecule 1 OS=Homo sapiens GN=NCAM1 PE=1 SV=1 - [A0A087WTF6_HUMAN]	14.74	1	22	93.27	4.9	0.48	2.36E-03
A0A140VK07	Testicular secretory protein Li 7 OS=Homo sapiens PE=2 SV=1 - [A0A140VK07_HUMAN]	32.31	13	30	73.86	6.4	0.48	1.05E-03
Q9NQG1	Protein MANBAL OS=Homo sapiens GN=MANBAL PE=1 SV=1 - [MANBL_HUMAN]	23.53	2	4	9.46	9.2	0.49	2.05E-03

P28161	Glutathione S-transferase Mu 2 OS=Homo sapiens GN=GSTM2 PE=1 SV=2 - [GSTM2_HUMAN]	61.47	8	31	25.73	6.4	0.49	4.40E-03
P31150	Rab GDP dissociation inhibitor alpha OS=Homo sapiens GN=GDI1 PE=1 SV=2 - [GDIA_HUMAN]	24.16	7	17	50.55	5.1	0.49	4.84E-03
V9GYG9	Apolipoprotein A-II (Fragment) OS=Homo sapiens GN=APOA2 PE=1 SV=1 - [V9GYG9_HUMAN]	41.05	4	8	10.64	6.6	0.49	3.01E-04
Q8TE57	A disintegrin and metalloproteinase with thrombospondin motifs 16 OS=Homo sapiens GN=ADAMTS16 PE=2 SV=3 - [ATS16_HUMAN]	3.92	3	4	136.11	8.6	0.49	4.02E-03
Q63ZY3	KN motif and ankyrin repeat domain-containing protein 2 OS=Homo sapiens GN=KANK2 PE=1 SV=1 - [KANK2_HUMAN]	25.03	16	17	91.12	5.6	0.49	2.09E-03
A0A024R5W6	Tropomyosin 1 (Alpha), isoform CRA_a OS=Homo sapiens GN=TPM1 PE=3 SV=1 - [A0A024R5W6_HUMAN]	47.18	1	38	32.66	4.7	0.49	7.59E-04
B4DLE4	cDNA FLJ54419, highly similar to Synaptopodin-2 OS=Homo sapiens PE=2 SV=1 - [B4DLE4_HUMAN]	3.58	2	3	74.72	9.4	0.49	1.90E-03
Q6UWY5	Olfactomedin-like protein 1 OS=Homo sapiens GN=OLFML1 PE=1 SV=2 - [OLFL1_HUMAN]	25.12	9	16	45.92	8.1	0.49	5.81E-04
A0A1B0GVV3	RILP-like protein 1 OS=Homo sapiens GN=RILPL1 PE=1 SV=1 - [A0A1B0GVV3_HUMAN]	3.11	1	1	44.74	5.1	0.49	3.37E-03
A0A075B6N8	Ig gamma-3 chain C region (Fragment) OS=Homo sapiens GN=IGHG3 PE=1 SV=1 - [A0A075B6N8_HUMAN]	47.21	5	78	41.30	7.9	0.49	1.96E-03
A8K3K1	cDNA FLJ78096, highly similar to Homo sapiens actin, alpha, cardiac muscle (ACTC), mRNA OS=Homo sapiens PE=2 SV=1 - [A8K3K1_HUMAN]	71.62	1	307	42.02	5.4	0.49	5.14E-03

F5H5G1	Limbic system-associated membrane protein OS=Homo sapiens GN=LSAMP PE=1 SV=2 - [F5H5G1_HUMAN]	3.82	1	2	31.72	5.9	0.49	2.42E-04
B2R5M1	cDNA, FLJ92527, highly similar to Homo sapiens cytochrome P450, family 17, subfamily A, polypeptide 1 (CYP17A1), mRNA OS=Homo sapiens PE=2 SV=1 - [B2R5M1_HUMAN]	5.91	3	7	57.28	8.7	0.49	9.74E-04
B3KXZ9	cDNA FLJ46477 fis, clone THYMU3025118, highly similar to Cell surface glycoprotein MUC18 OS=Homo sapiens PE=2 SV=1 - [B3KXZ9_HUMAN]	15.25	7	8	58.98	5.2	0.50	6.79E-04
Q12860	Contactin-1 OS=Homo sapiens GN=CNTN1 PE=1 SV=1 - [CNTN1_HUMAN]	4.42	4	4	113.25	5.9	0.50	1.66E-03
B2R8I2	cDNA, FLJ93914, highly similar to Homo sapiens histidine-rich glycoprotein (HRG), mRNA OS=Homo sapiens PE=2 SV=1 - [B2R8I2_HUMAN]	7.43	4	5	59.47	7.4	0.50	1.63E-03
Q4KMQ2	Anoctamin-6 OS=Homo sapiens GN=ANO6 PE=1 SV=2 - [ANO6_HUMAN]	7.8	4	6	106.10	7.8	0.50	2.64E-04
B1AJZ6	Protein Wnt (Fragment) OS=Homo sapiens GN=WNT4 PE=1 SV=1 - [B1AJZ6_HUMAN]	17.31	1	1	6.02	9.2	0.50	8.38E-04
Q7Z3B1	Neuronal growth regulator 1 OS=Homo sapiens GN=NEGR1 PE=1 SV=3 - [NEGR1_HUMAN]	11.58	2	2	38.69	6.2	0.50	2.01E-03
S6BGF5	IgG H chain OS=Homo sapiens PE=2 SV=1 - [S6BGF5_HUMAN]	20.42	2	11	25.58	8.6	0.50	1.61E-03
Q15124	Phosphoglucomutase-like protein 5 OS=Homo sapiens GN=PGM5 PE=1 SV=2 - [PGM5_HUMAN]	11.29	5	5	62.19	7.2	0.50	8.55E-04
J3QRI9	Developmentally-regulated GTP-binding protein 2 OS=Homo sapiens GN=DRG2 PE=1 SV=1 - [J3QRI9_HUMAN]	4.07	1	2	18.06	8.4	0.50	2.24E-03

A6NLN1	Polypyrimidine tract binding protein 1, isoform CRA_b OS=Homo sapiens GN=PTBP1 PE=1 SV=4 - [A6NLN1_HUMAN]	12.71	5	14	56.48	9.4	0.50	9.42E-04
P60903	Protein S100-A10 OS=Homo sapiens GN=S100A10 PE=1 SV=2 - [S10AA_HUMAN]	35.05	4	19	11.20	7.4	0.50	2.03E-04
A6NJJ6	UPF0575 protein C19orf67 OS=Homo sapiens GN=C19orf67 PE=3 SV=3 - [CS067_HUMAN]	6.15	1	1	39.75	4.9	0.50	5.77E-03
B4DPN0	cDNA FLJ51265, moderately similar to Beta-2-glycoprotein 1 (Beta-2-glycoprotein I) OS=Homo sapiens PE=2 SV=1 - [B4DPN0_HUMAN]	40.15	9	19	30.32	7.9	0.50	3.17E-04
E7ES33	Septin-7 OS=Homo sapiens GN=SEPT7 PE=1 SV=3 - [E7ES33_HUMAN]	30.46	11	23	48.69	8.7	0.50	2.70E-03
B4E0U6	cDNA FLJ53687, highly similar to Hsc70-interacting protein OS=Homo sapiens PE=2 SV=1 - [B4E0U6_HUMAN]	33.98	12	26	40.14	5.3	0.50	2.43E-04
A0A120HG46	GCT-A10 heavy chain variable region (Fragment) OS=Homo sapiens PE=2 SV=1 - [A0A120HG46_HUMAN]	12.6	2	3	13.59	8.8	0.50	1.89E-03
P60891	Ribose-phosphate pyrophosphokinase 1 OS=Homo sapiens GN=PRPS1 PE=1 SV=2 - [PRPS1_HUMAN]	25.79	2	15	34.81	7.0	0.50	9.47E-04
P09936	Ubiquitin carboxyl-terminal hydrolase isozyme L1 OS=Homo sapiens GN=UCHL1 PE=1 SV=2 - [UCHL1_HUMAN]	50.67	9	23	24.81	5.5	0.50	7.47E-05
Q9Y5S1	Transient receptor potential cation channel subfamily V member 2 OS=Homo sapiens GN=TRPV2 PE=1 SV=1 - [TRPV2_HUMAN]	1.18	1	1	85.93	5.8	0.50	2.24E-03
O76024	Wolframin OS=Homo sapiens GN=WFS1 PE=1 SV=2 - [WFS1_HUMAN]	20.34	15	23	100.23	8.0	0.50	4.52E-04

A0A087WWG1	Protein phosphatase Slingshot homolog 3 (Fragment) OS=Homo sapiens GN=SSH3 PE=1 SV=1 - [A0A087WWG1_HUMAN]	11.08	2	2	36.07	5.1	0.50	3.37E-03
P22694	cAMP-dependent protein kinase catalytic subunit beta OS=Homo sapiens GN=PRKACB PE=1 SV=2 - [KAPCB_HUMAN]	11.68	2	6	40.60	8.8	0.50	2.22E-03
B1AHA8	Heme oxygenase 1 (Fragment) OS=Homo sapiens GN=HMOX1 PE=1 SV=1 - [B1AHA8_HUMAN]	29.95	4	10	21.57	7.1	0.51	3.68E-04
Q9ULT8	E3 ubiquitin-protein ligase HECTD1 OS=Homo sapiens GN=HECTD1 PE=1 SV=3 - [HECD1_HUMAN]	1.19	2	3	289.20	5.4	0.51	1.01E-02
Q5T7C4	High mobility group protein B1 OS=Homo sapiens GN=HMGB1 PE=1 SV=1 - [Q5T7C4_HUMAN]	52.53	7	13	18.30	9.7	0.51	2.70E-03
B4DKV8	cDNA FLJ56665, highly similar to Nucleoporin Nup37 OS=Homo sapiens PE=2 SV=1 - [B4DKV8_HUMAN]	8.29	1	1	20.39	4.9	0.51	1.06E-05
Q5SSJ5	Heterochromatin protein 1-binding protein 3 OS=Homo sapiens GN=HP1BP3 PE=1 SV=1 - [HP1B3_HUMAN]	22.97	11	15	61.17	9.7	0.51	3.18E-04
Q9BUT1	3-hydroxybutyrate dehydrogenase type 2 OS=Homo sapiens GN=BDH2 PE=1 SV=2 - [BDH2_HUMAN]	17.14	4	6	26.71	7.6	0.51	1.81E-04
P16403	Histone H1.2 OS=Homo sapiens GN=HIST1H1C PE=1 SV=2 - [H12_HUMAN]	30.99	1	57	21.35	10.9	0.51	2.23E-03
P09382	Galectin-1 OS=Homo sapiens GN=LGALS1 PE=1 SV=2 - [LEG1_HUMAN]	57.04	7	48	14.71	5.5	0.51	2.53E-04
P27487	Dipeptidyl peptidase 4 OS=Homo sapiens GN=DPP4 PE=1 SV=2 - [DPP4_HUMAN]	7.05	6	9	88.22	6.0	0.51	1.70E-03

Q5TI65	Mitochondrial pyruvate carrier OS=Homo sapiens GN=BRP44L PE=1 SV=1 - [Q5TI65_HUMAN]	12.12	1	1	7.53	9.7	0.51	7.57E-04
A0A087WVT9	Nucleoside diphosphate kinase OS=Homo sapiens GN=NME4 PE=1 SV=1 - [A0A087WVT9_HUMAN]	11.11	1	2	16.77	10.9	0.51	3.39E-03
B0I1S0	DYNC2H1 variant protein OS=Homo sapiens PE=2 SV=1 - [B0I1S0_HUMAN]	0.88	2	4	492.34	6.5	0.51	2.49E-03
P08133	Annexin A6 OS=Homo sapiens GN=ANXA6 PE=1 SV=3 - [ANXA6_HUMAN]	60.62	1	152	75.83	5.6	0.51	1.28E-03
P22626	Heterogeneous nuclear ribonucleoproteins A2/B1 OS=Homo sapiens GN=HNRNPA2B1 PE=1 SV=2 - [ROA2_HUMAN]	19.26	6	20	37.41	9.0	0.51	1.27E-03
F5H3J7	CUGBP Elav-like family member 1 (Fragment) OS=Homo sapiens GN=CELF1 PE=1 SV=8 - [F5H3J7_HUMAN]	56.76	1	2	4.13	5.7	0.51	1.87E-02
B2R602	cDNA, FLJ92709, highly similar to Homo sapiens LanC lantibiotic synthetase component C-like 1 (bacterial) (LANCL1), mRNA OS=Homo sapiens PE=2 SV=1 - [B2R602_HUMAN]	14.04	4	6	45.27	7.9	0.51	5.96E-04
E5RIR0	Fatty acid-binding protein, adipocyte OS=Homo sapiens GN=FABP4 PE=1 SV=1 - [E5RIR0_HUMAN]	30.77	1	1	4.57	4.4	0.51	2.09E-02
Q15746	Myosin light chain kinase, smooth muscle OS=Homo sapiens GN=MYLK PE=1 SV=4 - [MYLK_HUMAN]	7.31	11	15	210.58	6.2	0.51	9.05E-04
B3KX20	cDNA FLJ44510 fis, clone UTERU3001652, highly similar to Leiomodin-1 OS=Homo sapiens PE=2 SV=1 - [B3KX20_HUMAN]	1.43	1	1	55.02	9.1	0.51	1.78E-03
P11766	Alcohol dehydrogenase class-3 OS=Homo sapiens GN=ADH5 PE=1 SV=4 - [ADHX_HUMAN]	30.75	11	28	39.70	7.5	0.52	6.16E-04

P28845	Corticosteroid 11-beta-dehydrogenase isozyme 1 OS=Homo sapiens GN=HSD11B1 PE=1 SV=3 - [DHI1_HUMAN]	26.71	7	17	32.38	8.6	0.52	2.22E-04
A0A024RC87	Ribonuclease/angiogenin inhibitor 1, isoform CRA_a OS=Homo sapiens GN=RNH1 PE=4 SV=1 - [A0A024RC87_HUMAN]	39.91	14	24	49.38	4.8	0.52	2.11E-04
Q4LEJ9	Prostaglandin transporter (Fragment) OS=Homo sapiens GN=SLC21A2 PE=4 SV=1 - [Q4LEJ9_HUMAN]	40.63	1	1	3.26	10.9	0.52	3.78E-03
B2R941	cDNA, FLJ94198, highly similar to Homo sapiens carboxypeptidase A3 (mast cell) (CPA3), mRNA OS=Homo sapiens PE=2 SV=1 - [B2R941_HUMAN]	11.27	5	7	48.68	9.0	0.52	9.91E-04
Q15599	Na(+)/H(+) exchange regulatory cofactor NHE-RF2 OS=Homo sapiens GN=SLC9A3R2 PE=1 SV=2 - [NHRF2_HUMAN]	4.15	1	1	37.39	7.9	0.52	4.12E-05
O60814	Histone H2B type 1-K OS=Homo sapiens GN=HIST1H2BK PE=1 SV=3 - [H2B1K_HUMAN]	57.94	2	118	13.88	10.3	0.52	4.19E-04
B4DMR3	cDNA FLJ51896, highly similar to Glia-derived nexin OS=Homo sapiens PE=2 SV=1 - [B4DMR3_HUMAN]	17.37	5	7	37.13	9.5	0.52	7.03E-04
O75954	Tetraspanin-9 OS=Homo sapiens GN=TSPAN9 PE=1 SV=1 - [TSN9_HUMAN]	4.6	1	1	26.76	7.7	0.52	3.45E-03
Q05DJ8	HTRA1 protein (Fragment) OS=Homo sapiens GN=HTRA1 PE=2 SV=1 - [Q05DJ8_HUMAN]	5.84	2	4	48.01	9.8	0.52	2.56E-03
B7Z997	cDNA FLJ54581, highly similar to Homo sapiens sorbin and SH3 domain containing 2 (SORBS2), transcript variant 1, mRNA OS=Homo sapiens PE=2 SV=1 - [B7Z997_HUMAN]	1.63	1	1	55.59	9.3	0.52	4.83E-03
D6RGI3	Septin 11, isoform CRA_b OS=Homo sapiens GN=SEPT11 PE=1 SV=1 - [D6RGI3_HUMAN]	24.71	3	24	48.98	7.0	0.52	2.91E-03

Q8TB36	Ganglioside-induced differentiation-associated protein 1 OS=Homo sapiens GN=GDAP1 PE=1 SV=3 - [GDAP1_HUMAN]	18.44	6	8	41.32	8.3	0.52	1.03E-02
Q05CW7	NAT10 protein (Fragment) OS=Homo sapiens GN=NAT10 PE=2 SV=1 - [Q05CW7_HUMAN]	2.17	1	1	62.35	9.3	0.52	1.27E-02
A0A0C4DH07	Latent-transforming growth factor beta-binding protein 4 OS=Homo sapiens GN=LTBP4 PE=1 SV=1 - [A0A0C4DH07_HUMAN]	2.71	3	3	169.34	5.3	0.52	2.48E-03
H0Y8B3	Adenosylhomocysteinase (Fragment) OS=Homo sapiens GN=AHCYL2 PE=1 SV=1 - [H0Y8B3_HUMAN]	16.41	1	9	58.08	8.0	0.52	4.41E-03
Q02952	A-kinase anchor protein 12 OS=Homo sapiens GN=AKAP12 PE=1 SV=4 - [AKA12_HUMAN]	24.58	10	44	191.37	4.4	0.52	3.24E-03
H0YE28	Protein SELENOH (Fragment) OS=Homo sapiens GN=SELENOH PE=1 SV=1 - [H0YE28_HUMAN]	9.57	1	1	10.43	8.9	0.52	1.08E-05
A0A087WYF8	PDZ and LIM domain protein 3 OS=Homo sapiens GN=PDLIM3 PE=1 SV=1 - [A0A087WYF8_HUMAN]	5.58	1	1	21.74	8.6	0.52	3.51E-03
B4E2M1	cDNA FLJ58478, highly similar to Glypican-6 OS=Homo sapiens PE=2 SV=1 - [B4E2M1_HUMAN]	7.71	2	4	48.53	6.8	0.53	3.01E-03
P23946	Chymase OS=Homo sapiens GN=CMA1 PE=1 SV=1 - [CMA1_HUMAN]	21.05	4	4	27.31	9.3	0.53	1.32E-03
H3BUU9	Cadherin-11 OS=Homo sapiens GN=CDH11 PE=1 SV=1 - [H3BUU9_HUMAN]	1.79	1	2	73.75	4.7	0.53	2.63E-03
P08758	Annexin A5 OS=Homo sapiens GN=ANXA5 PE=1 SV=2 - [ANXA5_HUMAN]	55.31	18	111	35.91	5.0	0.53	3.37E-04

D3DSW5	G protein-coupled receptor 124, isoform CRA_a OS=Homo sapiens GN=GPR124 PE=4 SV=1 - [D3DSW5_HUMAN]	2.45	2	2	139.27	8.5	0.53	2.71E-02
Q9GZT9	Egl nine homolog 1 OS=Homo sapiens GN=EGLN1 PE=1 SV=1 - [EGLN1_HUMAN]	5.63	1	1	45.99	8.5	0.53	4.51E-03
F5GXW1	Dedicator of cytokinesis protein 4 OS=Homo sapiens GN=DOCK4 PE=1 SV=2 - [F5GXW1_HUMAN]	1.19	1	1	125.98	7.9	0.53	2.29E-03
Q9NRN5	Olfactomedin-like protein 3 OS=Homo sapiens GN=OLFML3 PE=2 SV=1 - [OLFL3_HUMAN]	11.08	4	7	45.98	6.6	0.53	2.11E-03
Q9UDR5	Alpha-aminoadipic semialdehyde synthase, mitochondrial OS=Homo sapiens GN=AASS PE=1 SV=1 - [AASS_HUMAN]	29.81	20	37	102.07	6.6	0.53	7.82E-04
Q9UBI6	Guanine nucleotide-binding protein G(I)/G(S)/G(O) subunit gamma-12 OS=Homo sapiens GN=GNG12 PE=1 SV=3 - [GBG12_HUMAN]	77.78	6	13	8.00	9.0	0.53	4.63E-04
Q9HA72	Calcium homeostasis modulator protein 2 OS=Homo sapiens GN=CALHM2 PE=2 SV=1 - [CAHM2_HUMAN]	7.43	2	2	36.15	7.7	0.53	2.64E-03
Q9UH99	SUN domain-containing protein 2 OS=Homo sapiens GN=SUN2 PE=1 SV=3 - [SUN2_HUMAN]	32.91	14	21	80.26	6.7	0.53	1.03E-02
P62861	40S ribosomal protein S30 OS=Homo sapiens GN=FAU PE=1 SV=1 - [RS30_HUMAN]	16.95	1	2	6.64	12.1	0.53	3.97E-03
E9PIY1	Protein kinase C and casein kinase substrate in neurons protein 3 OS=Homo sapiens GN=PACSIN3 PE=1 SV=1 - [E9PIY1_HUMAN]	13.75	4	5	37.19	6.9	0.53	8.53E-03
K7EP07	Tubulin-folding cofactor B (Fragment) OS=Homo sapiens GN=TBCB PE=1 SV=8 - [K7EP07_HUMAN]	16.57	3	4	19.24	5.3	0.53	1.68E-02

Q5TBR0	Sialic acid synthase (Fragment) OS=Homo sapiens GN=NANS PE=1 SV=1 - [Q5TBR0_HUMAN]	15.65	1	1	12.39	5.8	0.53	3.41E-04
Q9BWP8	Collectin-11 OS=Homo sapiens GN=COLEC11 PE=1 SV=1 - [COL11_HUMAN]	4.06	1	1	28.65	5.4	0.53	7.65E-03
O60493	Sorting nexin-3 OS=Homo sapiens GN=SNX3 PE=1 SV=3 - [SNX3_HUMAN]	22.84	3	6	18.75	8.7	0.53	2.24E-02
H0Y7M8	Histone lysine demethylase PHF8 (Fragment) OS=Homo sapiens GN=PHF8 PE=1 SV=1 - [H0Y7M8_HUMAN]	11.19	1	1	15.45	6.7	0.54	7.12E-03
A0A024R5N4	WD repeat domain 71, isoform CRA_a OS=Homo sapiens GN=WDR71 PE=4 SV=1 - [A0A024R5N4_HUMAN]	2.13	1	1	40.41	6.7	0.54	8.17E-03
Q4TZM4	Hemoglobin beta chain (Fragment) OS=Homo sapiens GN=HBB PE=3 SV=1 - [Q4TZM4_HUMAN]	88.12	1	107	11.02	6.5	0.54	3.37E-02
A0A0A0MSU4	ATP-binding cassette sub-family A member 8 OS=Homo sapiens GN=ABCA8 PE=1 SV=1 - [A0A0A0MSU4_HUMAN]	9.78	12	20	182.95	7.1	0.54	2.56E-05
Q15019	Septin-2 OS=Homo sapiens GN=SEPT2 PE=1 SV=1 - [SEPT2_HUMAN]	51.8	12	26	41.46	6.6	0.54	1.82E-03
P58166	Inhibin beta E chain OS=Homo sapiens GN=INHBE PE=1 SV=1 - [INHBE_HUMAN]	8	2	3	38.54	9.2	0.54	1.51E-02
Q15417	Calponin-3 OS=Homo sapiens GN=CNN3 PE=1 SV=1 - [CNN3_HUMAN]	31	6	11	36.39	6.0	0.54	1.16E-03
A0A0S2Z4B5	Serine/threonine-protein phosphatase (Fragment) OS=Homo sapiens GN=PPP3CA PE=2 SV=1 - [A0A0S2Z4B5_HUMAN]	1.76	1	1	57.62	6.3	0.54	9.58E-04

H0Y544	Solute carrier family 23 member 2 (Fragment) OS=Homo sapiens GN=SLC23A2 PE=1 SV=1 - [H0Y544_HUMAN]	4.61	1	1	32.87	9.0	0.54	2.88E-03
P05204	Non-histone chromosomal protein HMG-17 OS=Homo sapiens GN=HMGN2 PE=1 SV=3 - [HMGN2_HUMAN]	8.89	1	2	9.39	10.0	0.54	2.74E-03
A0A0X9T0H6	GCT-A5 heavy chain variable region (Fragment) OS=Homo sapiens PE=2 SV=1 - [A0A0X9T0H6_HUMAN]	10.53	1	1	12.65	8.0	0.54	3.19E-03
P67809	Nuclease-sensitive element-binding protein 1 OS=Homo sapiens GN=YBX1 PE=1 SV=3 - [YBOX1_HUMAN]	20.99	3	6	35.90	9.9	0.54	4.22E-03
G4XID0	Rhesus blood group D antigen (Fragment) OS=Homo sapiens GN=RHD PE=4 SV=1 - [G4XID0_HUMAN]	37.5	1	4	2.99	8.6	0.54	1.55E-03
B4DVN5	cDNA FLJ55372, highly similar to FH1/FH2 domain-containing protein OS=Homo sapiens PE=2 SV=1 - [B4DVN5_HUMAN]	2.69	2	2	81.28	7.5	0.54	1.91E-03
B7Z938	cDNA FLJ61727, highly similar to Laminin alpha-4 chain (Fragment) OS=Homo sapiens PE=2 SV=1 - [B7Z938_HUMAN]	10.05	10	12	157.38	5.4	0.54	2.50E-04
H0Y4R5	Transmembrane protein 201 (Fragment) OS=Homo sapiens GN=TMEM201 PE=1 SV=1 - [H0Y4R5_HUMAN]	6.88	3	4	59.80	9.7	0.54	1.22E-03
A0A1B0GTR9	Tyrosine-protein kinase (Fragment) OS=Homo sapiens GN=JAK2 PE=1 SV=1 - [A0A1B0GTR9_HUMAN]	1.55	1	1	81.39	7.8	0.54	1.24E-03
Q8NC56	LEM domain-containing protein 2 OS=Homo sapiens GN=LEMD2 PE=1 SV=1 - [LEMD2_HUMAN]	17.5	8	17	56.94	9.0	0.54	1.33E-03
E9PHK0	Tetranectin OS=Homo sapiens GN=CLEC3B PE=1 SV=1 - [E9PHK0_HUMAN]	20	3	3	17.78	5.0	0.54	7.45E-04

B8ZZF0	Protein phosphatase 1B (Fragment) OS=Homo sapiens GN=PPM1B PE=1 SV=2 - [B8ZZF0_HUMAN]	4.62	1	1	33.21	6.3	0.54	6.97E-03
P62857	40S ribosomal protein S28 OS=Homo sapiens GN=RPS28 PE=1 SV=1 - [RS28_HUMAN]	46.38	3	8	7.84	10.7	0.54	5.04E-03
U3KPR1	Keratin, type II cuticular Hb6 (Fragment) OS=Homo sapiens GN=KRT86 PE=1 SV=1 - [U3KPR1_HUMAN]	9.79	1	8	15.37	8.5	0.54	5.75E-04
Q8N395	Putative uncharacterized protein DKFZp761N202 (Fragment) OS=Homo sapiens GN=DKFZp761N202 PE=2 SV=1 - [Q8N395_HUMAN]	3.82	1	2	34.96	10.0	0.54	3.13E-03
B4DY28	cDNA FLJ61189, highly similar to Cysteine and glycine-rich protein 1 OS=Homo sapiens PE=2 SV=1 - [B4DY28_HUMAN]	51.87	8	16	20.03	8.5	0.54	2.36E-04
J3QQN1	Proteasome subunit beta type-10 (Fragment) OS=Homo sapiens GN=PSMB10 PE=1 SV=1 - [J3QQN1_HUMAN]	7.97	1	1	14.20	6.8	0.54	1.67E-03
Q13418	Integrin-linked protein kinase OS=Homo sapiens GN=ILK PE=1 SV=2 - [ILK_HUMAN]	21.9	10	19	51.39	8.1	0.54	2.20E-04
F8VWL0	Alpha-globin transcription factor CP2 (Fragment) OS=Homo sapiens GN=TFCP2 PE=1 SV=1 - [F8VWL0_HUMAN]	3.15	1	1	43.91	7.0	0.54	3.60E-03
B4E278	cDNA FLJ55571, highly similar to Sad1/unc-84 protein-like 1 OS=Homo sapiens PE=2 SV=1 - [B4E278_HUMAN]	12.18	6	12	66.14	6.5	0.54	6.20E-06
P30086	Phosphatidylethanolamine-binding protein 1 OS=Homo sapiens GN=PEBP1 PE=1 SV=3 - [PEBP1_HUMAN]	71.66	9	17	21.04	7.5	0.55	3.07E-04
Q5T619	Zinc finger protein 648 OS=Homo sapiens GN=ZNF648 PE=2 SV=1 - [ZN648_HUMAN]	2.11	1	2	62.30	8.6	0.55	9.94E-04

Q56G89	Serum albumin OS=Homo sapiens PE=2 SV=1 - [Q56G89_HUMAN]	72.58	1	919	69.04	6.2	0.55	1.08E-02
Q96PD5	N-acetylmuramoyl-L-alanine amidase OS=Homo sapiens GN=PGLYRP2 PE=1 SV=1 - [PGRP2_HUMAN]	6.25	2	2	62.18	7.5	0.55	7.22E-03
P02649	Apolipoprotein E OS=Homo sapiens GN=APOE PE=1 SV=1 - [APOE_HUMAN]	54.89	17	29	36.13	5.7	0.55	2.73E-04
Q9NVD7	Alpha-parvin OS=Homo sapiens GN=PARVA PE=1 SV=1 - [PARVA_HUMAN]	18.82	6	12	42.22	5.9	0.55	6.72E-04
Q16658	Fascin OS=Homo sapiens GN=FSCN1 PE=1 SV=3 - [FSCN1_HUMAN]	26.77	10	18	54.50	7.2	0.55	1.58E-03
P01859	Ig gamma-2 chain C region OS=Homo sapiens GN=IGHG2 PE=1 SV=2 - [IGHG2_HUMAN]	45.4	5	59	35.88	7.6	0.55	9.01E-04
Q5T9B9	Endoglin (Osler-Rendu-Weber syndrome 1) OS=Homo sapiens GN=ENG PE=4 SV=1 - [Q5T9B9_HUMAN]	7.84	4	5	67.50	6.6	0.55	5.22E-04
P19784	Casein kinase II subunit alpha' OS=Homo sapiens GN=CSNK2A2 PE=1 SV=1 - [CSK22_HUMAN]	8.86	2	4	41.19	8.6	0.55	1.12E-03
P55001	Microfibrillar-associated protein 2 OS=Homo sapiens GN=MFAP2 PE=2 SV=1 - [MFAP2_HUMAN]	9.84	2	5	20.81	5.0	0.55	7.23E-03
Q3SY61	KCNT2 protein OS=Homo sapiens GN=KCNT2 PE=1 SV=1 - [Q3SY61_HUMAN]	5.95	1	1	41.89	6.7	0.55	2.25E-03
Q8N8Q8	Mitochondrial inner membrane protein COX18 OS=Homo sapiens GN=COX18 PE=2 SV=1 - [COX18_HUMAN]	2.7	1	1	37.04	9.8	0.55	5.41E-04

Q59EE7	Pro-alpha-1 type V collagen variant (Fragment) OS=Homo sapiens PE=2 SV=1 - [Q59EE7_HUMAN]	1.56	3	6	178.41	5.0	0.55	5.88E-03
Q96IZ0	PRKC apoptosis WT1 regulator protein OS=Homo sapiens GN=PAWR PE=1 SV=1 - [PAWR_HUMAN]	3.82	1	1	36.55	5.4	0.55	4.98E-03
A0A0D9SGF6	Spectrin alpha chain, non-erythrocytic 1 OS=Homo sapiens GN=SPTAN1 PE=1 SV=1 - [A0A0D9SGF6_HUMAN]	41.55	1	179	287.43	5.4	0.55	1.79E-02
B2RAA8	cDNA, FLJ94802 OS=Homo sapiens PE=2 SV=1 - [B2RAA8_HUMAN]	3.55	1	1	31.12	5.1	0.55	5.85E-03
Q9H330	Transmembrane protein 245 OS=Homo sapiens GN=TMEM245 PE=1 SV=2 - [TM245_HUMAN]	3.73	3	4	100.88	8.9	0.55	1.07E-02
H0Y485	Insulin-like growth factor-binding protein 3 (Fragment) OS=Homo sapiens GN=IGFBP3 PE=1 SV=1 - [H0Y485_HUMAN]	16.78	3	5	16.54	9.4	0.55	9.89E-04
V9GY97	Amyloid beta A4 precursor protein-binding family B member 1 (Fragment) OS=Homo sapiens GN=APBB1 PE=1 SV=8 - [V9GY97_HUMAN]	7.28	1	1	29.12	5.3	0.56	1.10E-02
A0A193CHQ9	10E8 heavy chain variable region (Fragment) OS=Homo sapiens PE=2 SV=1 - [A0A193CHQ9_HUMAN]	19.85	2	5	14.47	7.9	0.56	1.14E-03
P06396	Gelsolin OS=Homo sapiens GN=GSN PE=1 SV=1 - [GELS_HUMAN]	31.2	2	82	85.64	6.3	0.56	2.74E-03
A1Z1Q3	O-acetyl-ADP-ribose deacetylase MACROD2 OS=Homo sapiens GN=MACROD2 PE=1 SV=1 - [MACD2_HUMAN]	2.46	1	1	50.02	4.6	0.56	4.31E-03
E9PNW4	CD59 glycoprotein OS=Homo sapiens GN=CD59 PE=1 SV=1 - [E9PNW4_HUMAN]	27.78	3	13	11.98	5.7	0.56	8.55E-04

I7H119	RAB2, member RAS oncogene family-like (Fragment) OS=Homo sapiens GN=RGL2 PE=4 SV=1 - [I7H119_HUMAN]	9.33	1	1	28.66	5.5	0.56	5.86E-03
Q96H79	Zinc finger CCCH-type antiviral protein 1-like OS=Homo sapiens GN=ZC3HAV1L PE=1 SV=2 - [ZCCHL_HUMAN]	14	3	3	32.94	8.1	0.56	1.04E-02
B2R7F8	Plasminogen OS=Homo sapiens PE=2 SV=1 - [B2R7F8_HUMAN]	30	17	31	90.48	7.2	0.56	8.30E-04
A8K4W5	cDNA FLJ76813, highly similar to Homo sapiens acetyl-Coenzyme A acetyltransferase 2 (acetoacetyl Coenzyme A thiolase), mRNA OS=Homo sapiens PE=2 SV=1 - [A8K4W5_HUMAN]	11.59	3	5	41.35	6.9	0.56	3.16E-03
Q8N227	cDNA FLJ36253 fis, clone THYMU2002154 OS=Homo sapiens PE=2 SV=1 - [Q8N227_HUMAN]	2.64	1	1	55.69	6.0	0.56	4.74E-03
P39019	40S ribosomal protein S19 OS=Homo sapiens GN=RPS19 PE=1 SV=2 - [RS19_HUMAN]	39.31	6	25	16.05	10.3	0.56	3.77E-04
P36021	Monocarboxylate transporter 8 OS=Homo sapiens GN=SLC16A2 PE=1 SV=2 - [MOT8_HUMAN]	2.41	1	1	59.47	5.6	0.56	3.06E-03
Q01118	Sodium channel protein type 7 subunit alpha OS=Homo sapiens GN=SCN7A PE=1 SV=2 - [SCN7A_HUMAN]	3.45	5	6	193.37	8.0	0.56	1.07E-04
A0A0C4DGM1	Tryptase alpha/beta-1 (Fragment) OS=Homo sapiens GN=TPSAB1 PE=1 SV=1 - [A0A0C4DGM1_HUMAN]	20.66	4	6	23.88	6.4	0.56	2.05E-03
A2J1N0	Rheumatoid factor RF-IP14 (Fragment) OS=Homo sapiens PE=2 SV=1 - [A2J1N0_HUMAN]	18.75	1	3	10.49	9.0	0.56	2.78E-03
A0A140T8Y3	Tenascin-X OS=Homo sapiens GN=TNXB PE=1 SV=1 - [A0A140T8Y3_HUMAN]	6.13	14	22	455.86	5.2	0.56	7.63E-04

Q9HAV0	Guanine nucleotide-binding protein subunit beta-4 OS=Homo sapiens GN=GNB4 PE=1 SV=3 - [GBB4_HUMAN]	25.88	3	23	37.54	6.0	0.56	1.85E-04
Q16555	Dihydropyrimidinase-related protein 2 OS=Homo sapiens GN=DPYSL2 PE=1 SV=1 - [DPYL2_HUMAN]	35.84	5	40	62.25	6.4	0.56	2.54E-03
B2R960	cDNA, FLJ94230, highly similar to Homo sapiens thioredoxin-like 1 (TXNL1), mRNA OS=Homo sapiens PE=2 SV=1 - [B2R960_HUMAN]	21.8	4	8	32.22	5.0	0.56	1.40E-03
Q6N095	Uncharacterized protein OS=Homo sapiens GN=DKFZp686K03196 PE=1 SV=1 - [Q6N095_HUMAN]	40.21	1	156	52.33	8.6	0.56	6.32E-04
D2CPK1	Mutant mutant xeroderma pigmentosum complementation group C protein (Fragment) OS=Homo sapiens PE=4 SV=1 - [D2CPK1_HUMAN]	13.29	1	1	15.93	5.2	0.56	6.86E-03
A5PKY0	EPS15L1 protein (Fragment) OS=Homo sapiens GN=EPS15L1 PE=2 SV=1 - [A5PKY0_HUMAN]	2.83	2	3	66.31	5.3	0.56	1.52E-04
A0A0C4DG84	Anthrax toxin receptor 2 OS=Homo sapiens GN=ANTXR2 PE=1 SV=1 - [A0A0C4DG84_HUMAN]	9.39	2	2	26.09	6.8	0.56	1.86E-04
B4DPZ3	cDNA FLJ53290, highly similar to Cytoplasmic dynein 1 intermediate chain 2 OS=Homo sapiens PE=2 SV=1 - [B4DPZ3_HUMAN]	12.22	5	8	70.41	5.3	0.56	1.12E-03
O75084	Frizzled-7 OS=Homo sapiens GN=FZD7 PE=1 SV=2 - [FZD7_HUMAN]	7.32	3	7	63.58	7.8	0.56	1.36E-03
I1VSB5	Siglec-16 (Fragment) OS=Homo sapiens GN=SIGLEC16 PE=4 SV=1 - [I1VSB5_HUMAN]	2.11	1	1	47.22	9.0	0.56	1.12E-03
B9EGI2	Myosin phosphatase Rho interacting protein OS=Homo sapiens GN=MPRIP PE=2 SV=1 - [B9EGI2_HUMAN]	2.51	2	2	117.97	6.4	0.56	2.34E-02

Q6FG99	RPLP1 protein OS=Homo sapiens GN=RPLP1 PE=2 SV=1 - [Q6FG99_HUMAN]	44.74	2	8	11.56	4.4	0.56	1.05E-03
P32189	Glycerol kinase OS=Homo sapiens GN=GK PE=1 SV=3 - [GLPK_HUMAN]	15.56	8	9	61.21	6.5	0.56	4.99E-03
P02656	Apolipoprotein C-III OS=Homo sapiens GN=APOC3 PE=1 SV=1 - [APOC3_HUMAN]	34.34	3	5	10.85	5.4	0.56	7.16E-04
B1AP13	Complement decay-accelerating factor OS=Homo sapiens GN=CD55 PE=1 SV=1 - [B1AP13_HUMAN]	18.69	6	9	49.31	8.8	0.56	5.20E-04
F5H364	Protein Wnt (Fragment) OS=Homo sapiens GN=WNT5B PE=1 SV=1 - [F5H364_HUMAN]	7.01	1	1	17.33	8.6	0.56	8.72E-03
E9PJP1	Mth938 domain-containing protein OS=Homo sapiens GN=AAMDC PE=1 SV=1 - [E9PJP1_HUMAN]	17.2	1	1	10.12	8.0	0.57	1.46E-04
Q9UFM8	Neuroplastin (Fragment) OS=Homo sapiens GN=DKFZp566H1924 PE=1 SV=2 - [Q9UFM8_HUMAN]	6.17	2	2	34.79	7.7	0.57	8.75E-03
B7Z6Y2	cDNA FLJ54942, highly similar to Homo sapiens bridging integrator 1 (BIN1), transcript variant 10, mRNA OS=Homo sapiens PE=2 SV=1 - [B7Z6Y2_HUMAN]	4.94	1	2	43.14	5.3	0.57	4.15E-03
A0A024RAB6	Heparan sulfate proteoglycan 2 (Perlecan), isoform CRA_b OS=Homo sapiens GN=HSPG2 PE=4 SV=1 - [A0A024RAB6_HUMAN]	12.91	41	64	463.72	6.5	0.57	1.21E-03
C9JR67	Epsilon-sarcoglycan OS=Homo sapiens GN=SGCE PE=1 SV=1 - [C9JR67_HUMAN]	14.02	5	6	48.72	6.6	0.57	8.33E-04
P27816	Microtubule-associated protein 4 OS=Homo sapiens GN=MAP4 PE=1 SV=3 - [MAP4_HUMAN]	8.07	7	8	120.93	5.4	0.57	4.34E-03

P30050	60S ribosomal protein L12 OS=Homo sapiens GN=RPL12 PE=1 SV=1 - [RL12_HUMAN]	44.85	5	12	17.81	9.4	0.57	1.62E-03
P35625	Metalloproteinase inhibitor 3 OS=Homo sapiens GN=TIMP3 PE=1 SV=2 - [TIMP3_HUMAN]	19.43	4	7	24.13	8.7	0.57	6.23E-04
E9PHR9	Phospholipid scramblase 4 OS=Homo sapiens GN=PLSCR4 PE=1 SV=1 - [E9PHR9_HUMAN]	5.44	1	1	26.23	6.1	0.57	1.71E-02
Q7Z513	Raslp2 OS=Homo sapiens PE=2 SV=1 - [Q7Z513_HUMAN]	11.68	1	5	23.69	6.6	0.57	2.41E-03
M4VR43	Apolipoprotein B editing enzyme catalytic polypeptide-like 3F OS=Homo sapiens GN=APOBEC3F PE=2 SV=1 - [M4VR43_HUMAN]	10.46	2	4	45.02	7.2	0.57	2.71E-03
P15121	Aldose reductase OS=Homo sapiens GN=AKR1B1 PE=1 SV=3 - [ALDR_HUMAN]	20.25	6	17	35.83	7.0	0.57	4.64E-03
I6LM39	Mutant methyl CpG binding protein 2 OS=Homo sapiens GN=MECP2 PE=2 SV=1 - [I6LM39_HUMAN]	18.82	4	8	29.36	10.3	0.57	5.10E-04
Q9NWV4	UPF0587 protein C1orf123 OS=Homo sapiens GN=C1orf123 PE=1 SV=1 - [CA123_HUMAN]	20.63	3	6	18.04	5.0	0.57	1.81E-03
Q59FM5	Guanine nucleotide-binding protein G, alpha subunit variant (Fragment) OS=Homo sapiens PE=2 SV=1 - [Q59FM5_HUMAN]	22.97	3	22	41.86	5.7	0.57	3.14E-03
A0A169TED2	Protein kinase C OS=Homo sapiens GN=PRKCA PE=2 SV=1 - [A0A169TED2_HUMAN]	11.61	6	10	76.64	7.5	0.57	4.56E-03
B2R9V7	Superoxide dismutase [Cu-Zn] OS=Homo sapiens PE=2 SV=1 - [B2R9V7_HUMAN]	26.67	5	9	25.88	6.8	0.57	3.96E-03

B3KN52	cDNA FLJ13606 fis, clone PLACE1010579, highly similar to Homo sapiens GULP, engulfment adaptor PTB domain containing 1 (GULP1), mRNA OS=Homo sapiens PE=2 SV=1 - [B3KN52_HUMAN]	5.26	2	2	34.44	7.9	0.57	1.76E-03
Q13425	Beta-2-syntrophin OS=Homo sapiens GN=SNTB2 PE=1 SV=1 - [SNTB2_HUMAN]	25	11	19	57.91	8.8	0.57	8.50E-03
Q96K76	Ubiquitin carboxyl-terminal hydrolase 47 OS=Homo sapiens GN=USP47 PE=1 SV=3 - [UBP47_HUMAN]	1.67	2	2	157.21	5.1	0.57	1.97E-02
P36269	Gamma-glutamyltransferase 5 OS=Homo sapiens GN=GGT5 PE=1 SV=2 - [GGT5_HUMAN]	13.82	7	13	62.22	7.5	0.57	1.96E-03
J3QRJ1	Secernin-2 OS=Homo sapiens GN=SCRN2 PE=1 SV=1 - [J3QRJ1_HUMAN]	14.39	2	2	29.57	6.0	0.57	1.04E-02
P17612	cAMP-dependent protein kinase catalytic subunit alpha OS=Homo sapiens GN=PRKACA PE=1 SV=2 - [KAPCA_HUMAN]	22.22	4	8	40.56	8.8	0.57	1.15E-03
P51692	Signal transducer and activator of transcription 5B OS=Homo sapiens GN=STAT5B PE=1 SV=2 - [STA5B_HUMAN]	4.45	1	3	89.81	6.0	0.57	2.10E-03
Q9BS40	Latexin OS=Homo sapiens GN=LXN PE=1 SV=2 - [LXN_HUMAN]	16.67	3	7	25.73	5.8	0.57	3.74E-03
P09619	Platelet-derived growth factor receptor beta OS=Homo sapiens GN=PDGFRB PE=1 SV=1 - [PGFRB_HUMAN]	7.32	5	10	123.89	5.0	0.57	1.50E-02
Q04760	Lactoylglutathione lyase OS=Homo sapiens GN=GLO1 PE=1 SV=4 - [LGUL_HUMAN]	29.35	5	8	20.76	5.3	0.57	1.74E-04
O95425	Supervillin OS=Homo sapiens GN=SVIL PE=1 SV=2 - [SVIL_HUMAN]	5.28	9	13	247.59	7.0	0.57	1.38E-03

Q2NLD4	PURA protein (Fragment) OS=Homo sapiens GN=PURA PE=2 SV=1 - [Q2NLD4_HUMAN]	24.21	5	10	32.00	7.0	0.57	9.17E-04
M0QZP8	Immunity-related GTPase family Q protein (Fragment) OS=Homo sapiens GN=IRGQ PE=1 SV=1 - [M0QZP8_HUMAN]	10.29	1	1	13.49	4.5	0.58	1.22E-03
P35052	Glypican-1 OS=Homo sapiens GN=GPC1 PE=1 SV=2 - [GPC1_HUMAN]	15.05	6	7	61.64	7.3	0.58	6.03E-03
Q59F44	Cytochrome b-5 isoform 1 variant (Fragment) OS=Homo sapiens PE=2 SV=1 - [Q59F44_HUMAN]	50	5	20	14.56	5.7	0.58	4.80E-05
Q53QV1	Putative uncharacterized protein DTNB (Fragment) OS=Homo sapiens GN=DTNB PE=4 SV=1 - [Q53QV1_HUMAN]	20	1	6	19.26	6.1	0.58	6.00E-04
P17655	Calpain-2 catalytic subunit OS=Homo sapiens GN=CAPN2 PE=1 SV=6 - [CAN2_HUMAN]	15.43	7	13	79.94	5.0	0.58	2.52E-03
Q59FC9	Enigma homolog (Fragment) OS=Homo sapiens PE=2 SV=1 - [Q59FC9_HUMAN]	12.61	1	6	48.93	7.9	0.58	1.03E-03
A0A024R035	Complement component 9, isoform CRA_a OS=Homo sapiens GN=C9 PE=4 SV=1 - [A0A024R035_HUMAN]	18.78	9	24	63.16	5.5	0.58	1.36E-03
A0A087WZK9	Eukaryotic translation initiation factor 3 subunit H OS=Homo sapiens GN=EIF3H PE=1 SV=1 - [A0A087WZK9_HUMAN]	22.92	6	9	39.56	6.4	0.58	3.64E-03
F8WFC3	Regulator of microtubule dynamics protein 2 OS=Homo sapiens GN=RMDN2 PE=1 SV=1 - [F8WFC3_HUMAN]	11.88	2	5	18.15	6.2	0.58	6.18E-03
B4DKX4	cDNA FLJ58014, highly similar to Homo sapiens programmed cell death 4, transcript variant 1, mRNA OS=Homo sapiens PE=2 SV=1 - [B4DKX4_HUMAN]	28.79	10	13	50.20	5.5	0.58	1.69E-03

Q01082	Spectrin beta chain, non-erythrocytic 1 OS=Homo sapiens GN=SPTBN1 PE=1 SV=2 - [SPTB2_HUMAN]	38.92	69	158	274.44	5.6	0.58	4.13E-04
Q53FL4	Adducin 3 isoform a variant (Fragment) OS=Homo sapiens PE=2 SV=1 - [Q53FL4_HUMAN]	5.4	3	6	74.43	6.5	0.58	4.94E-04
F6KPG5	Albumin (Fragment) OS=Homo sapiens PE=2 SV=1 - [F6KPG5_HUMAN]	83.59	1	1029	66.49	6.0	0.58	4.57E-03
A0A087WYF1	Laminin subunit alpha-2 OS=Homo sapiens GN=LAMA2 PE=1 SV=1 - [A0A087WYF1_HUMAN]	3.85	10	12	343.20	6.4	0.58	3.10E-03
B2R932	cDNA, FLJ94187, highly similar to Homo sapiens CD99 antigen (CD99), mRNA OS=Homo sapiens PE=2 SV=1 - [B2R932_HUMAN]	18.38	3	10	18.94	4.8	0.58	2.77E-03
B4DEY6	LIM and cysteine-rich domains protein 1 OS=Homo sapiens GN=LMCD1 PE=1 SV=1 - [B4DEY6_HUMAN]	25.3	5	7	28.49	5.9	0.58	9.21E-05
F8W150	Ankyrin repeat domain-containing protein 13A (Fragment) OS=Homo sapiens GN=ANKRD13A PE=1 SV=1 - [F8W150_HUMAN]	14.46	1	1	9.37	6.8	0.58	2.39E-03
P16157	Ankyrin-1 OS=Homo sapiens GN=ANK1 PE=1 SV=3 - [ANK1_HUMAN]	14.14	19	44	206.14	6.0	0.58	2.37E-04
Q66K74	Microtubule-associated protein 1S OS=Homo sapiens GN=MAP1S PE=1 SV=2 - [MAP1S_HUMAN]	7.55	6	9	112.14	7.3	0.58	1.89E-03
H0YMA2	Tropomodulin-2 (Fragment) OS=Homo sapiens GN=TMOD2 PE=1 SV=1 - [H0YMA2_HUMAN]	12.73	2	7	12.38	9.4	0.59	7.44E-04
P46439	Glutathione S-transferase Mu 5 OS=Homo sapiens GN=GSTM5 PE=1 SV=3 - [GSTM5_HUMAN]	36.7	2	25	25.66	7.4	0.59	4.01E-04

Q5VU21	PAI-1 mRNA-binding protein variant OS=Homo sapiens PE=2 SV=1 - [Q5VU21_HUMAN]	9.3	3	6	42.40	8.4	0.59	1.91E-03
B4DKV4	cDNA FLJ60647, highly similar to Keratin, type II cytoskeletal 6B OS=Homo sapiens PE=2 SV=1 - [B4DKV4_HUMAN]	17.87	1	21	55.70	8.4	0.59	1.16E-02
Q93097	Protein Wnt-2b OS=Homo sapiens GN=WNT2B PE=1 SV=2 - [WNT2B_HUMAN]	9.72	3	6	43.74	9.0	0.59	4.55E-04
Q8WY78	Chromosome 20 open reading frame 161, isoform CRA_a OS=Homo sapiens GN=C20orf161 PE=2 SV=1 - [Q8WY78_HUMAN]	8.15	1	1	20.60	8.2	0.59	5.60E-03
P02766	Transthyretin OS=Homo sapiens GN=TTR PE=1 SV=1 - [TTHY_HUMAN]	48.3	4	7	15.88	5.8	0.59	3.60E-03
E7EV99	Alpha-adducin OS=Homo sapiens GN=ADD1 PE=1 SV=1 - [E7EV99_HUMAN]	12.82	6	6	70.01	6.5	0.59	9.21E-04
A0A024RB48	Tensin like C1 domain containing phosphatase (Tensin 2), isoform CRA_a OS=Homo sapiens GN=TENC1 PE=4 SV=1 - [A0A024RB48_HUMAN]	6.61	4	7	138.74	7.4	0.59	2.09E-03
E9PRU1	EGF-containing fibulin-like extracellular matrix protein 2 OS=Homo sapiens GN=EFEMP2 PE=1 SV=1 - [E9PRU1_HUMAN]	4.59	2	2	48.16	5.0	0.59	2.89E-03
A0A0A0MR85	Glutathione S-transferase Mu 4 OS=Homo sapiens GN=GSTM4 PE=1 SV=1 - [A0A0A0MR85_HUMAN]	26.61	1	14	25.55	5.9	0.59	8.21E-03
Q59FT7	Mitogen-activated protein kinase kinase kinase 7 interacting protein 1 isoform alpha variant (Fragment) OS=Homo sapiens PE=2 SV=1 - [Q59FT7_HUMAN]	4.94	2	2	70.22	5.3	0.59	6.22E-03
Q14643	Inositol 1,4,5-trisphosphate receptor type 1 OS=Homo sapiens GN=ITPR1 PE=1 SV=3 - [ITPR1_HUMAN]	12.8	23	48	313.73	6.0	0.59	6.55E-05

P12270	Nucleoprotein TPR OS=Homo sapiens GN=TPR PE=1 SV=3 - [TPR_HUMAN]	1.61	2	2	267.13	5.0	0.59	1.24E-04
Q01970	1-phosphatidylinositol 4,5-bisphosphate phosphodiesterase beta-3 OS=Homo sapiens GN=PLCB3 PE=1 SV=2 - [PLCB3_HUMAN]	4.38	4	4	138.71	5.9	0.59	1.38E-03
M0R328	Peroxisomal membrane protein 11C (Fragment) OS=Homo sapiens GN=PEX11G PE=1 SV=1 - [M0R328_HUMAN]	8.33	1	1	18.85	8.7	0.59	2.19E-04
H0YAN8	Rho guanine nucleotide exchange factor 10 (Fragment) OS=Homo sapiens GN=ARHGEF10 PE=1 SV=1 - [H0YAN8_HUMAN]	1.11	1	1	109.58	8.0	0.59	4.45E-03
Q53FP2	Transmembrane protein 35A OS=Homo sapiens GN=TMEM35A PE=2 SV=2 - [TM35A_HUMAN]	18.56	3	4	18.43	10.1	0.59	6.42E-03
C9J5H8	Neurochondrin (Fragment) OS=Homo sapiens GN=NCDN PE=1 SV=1 - [C9J5H8_HUMAN]	4.92	1	1	26.35	5.2	0.59	4.14E-03
Q7KYR7	Butyrophilin subfamily 2 member A1 OS=Homo sapiens GN=BTN2A1 PE=1 SV=3 - [BT2A1_HUMAN]	4.36	2	2	59.59	6.5	0.59	2.25E-02
P25685	DnaJ homolog subfamily B member 1 OS=Homo sapiens GN=DNAJB1 PE=1 SV=4 - [DNJB1_HUMAN]	13.82	5	5	38.02	8.6	0.59	6.88E-03
B2RCG5	Dimethylaniline monooxygenase [N-oxide-forming] OS=Homo sapiens PE=2 SV=1 - [B2RCG5_HUMAN]	4.32	1	2	60.27	7.4	0.59	1.67E-04
A0A024RDI4	Ankyrin 2, neuronal, isoform CRA_a OS=Homo sapiens GN=ANK2 PE=4 SV=1 - [A0A024RDI4_HUMAN]	6.81	6	32	203.31	6.2	0.59	5.70E-03
B4DMB1	cDNA FLJ53358, highly similar to Heterogeneous nuclear ribonucleoprotein R OS=Homo sapiens PE=2 SV=1 - [B4DMB1_HUMAN]	10.08	3	11	66.97	8.3	0.59	1.41E-02

P24468	COUP transcription factor 2 OS=Homo sapiens GN=NR2F2 PE=1 SV=1 - [COT2_HUMAN]	10.39	3	3	45.54	8.3	0.59	4.33E-04
A0A0R7FJH5	Coagulation factor XII OS=Homo sapiens GN=F12 PE=3 SV=1 - [A0A0R7FJH5_HUMAN]	2.6	2	2	67.82	7.8	0.59	7.32E-03
A0A0E3KII0	Low density lipoprotein receptor (Fragment) OS=Homo sapiens GN=LDLR PE=4 SV=1 - [A0A0E3KII0_HUMAN]	8.04	1	1	12.57	7.3	0.59	9.06E-04
P12956	X-ray repair cross-complementing protein 6 OS=Homo sapiens GN=XRCC6 PE=1 SV=2 - [XRCC6_HUMAN]	46.8	28	76	69.80	6.6	0.59	2.97E-04
A8K6S3	cDNA FLJ77570, highly similar to Homo sapiens pleckstrin homology domain containing, family C (with FERM domain) member 1 (PLEKHC1), mRNA OS=Homo sapiens PE=2 SV=1 - [A8K6S3_HUMAN]	20.44	11	17	77.83	6.7	0.60	6.08E-03
P08670	Vimentin OS=Homo sapiens GN=VIM PE=1 SV=4 - [VIME_HUMAN]	65.02	25	189	53.62	5.1	0.60	5.68E-04
Q969G5	Protein kinase C delta-binding protein OS=Homo sapiens GN=PRKCDBP PE=1 SV=3 - [PRDBP_HUMAN]	18.77	5	8	27.68	6.4	0.60	2.11E-03
Q53T40	Putative uncharacterized protein FHL2 (Fragment) OS=Homo sapiens GN=FHL2 PE=4 SV=1 - [Q53T40_HUMAN]	39.88	7	9	19.29	8.1	0.60	5.09E-03
P49454	Centromere protein F OS=Homo sapiens GN=CENPF PE=1 SV=2 - [CENPF_HUMAN]	0.56	2	2	367.54	5.1	0.60	3.20E-03
E7EW69	Septin-10 OS=Homo sapiens GN=SEPT10 PE=1 SV=1 - [E7EW69_HUMAN]	24.22	5	9	52.11	6.9	0.60	3.14E-03
E7ENN3	Nesprin-1 OS=Homo sapiens GN=SYNE1 PE=1 SV=2 - [E7ENN3_HUMAN]	0.61	4	5	964.24	5.5	0.60	1.05E-02

B4DP97	cDNA FLJ52934, highly similar to Tyrosine-protein phosphatase non-receptortype substrate 1 OS=Homo sapiens PE=2 SV=1 - [B4DP97_HUMAN]	8.26	3	5	52.87	7.5	0.60	6.42E-03
B4E3S6	cDNA FLJ58413, highly similar to Complement component C7 OS=Homo sapiens PE=2 SV=1 - [B4E3S6_HUMAN]	17.49	6	10	53.68	5.4	0.60	4.66E-03
B2RTQ2	TBC1 domain family, member 2B, isoform CRA_b OS=Homo sapiens GN=TBC1D2B PE=2 SV=1 - [B2RTQ2_HUMAN]	1.43	1	1	96.64	6.0	0.60	1.23E-03
Q86YZ3	Hornerin OS=Homo sapiens GN=HRNR PE=1 SV=2 - [HORN_HUMAN]	1.93	1	3	282.23	10.0	0.60	3.91E-02
B3KU30	cDNA FLJ39125 fis, clone NTONG2007034, highly similar to Homo sapiens collagen, type XXI, alpha 1 (COL21A1), mRNA OS=Homo sapiens PE=2 SV=1 - [B3KU30_HUMAN]	3.5	1	1	33.15	9.2	0.60	1.57E-02
P19623	Spermidine synthase OS=Homo sapiens GN=SRM PE=1 SV=1 - [SPEE_HUMAN]	6.95	2	2	33.80	5.5	0.60	8.63E-03
A0M8Q2	Phosphatase 2C motif OS=Homo sapiens PE=3 SV=1 - [A0M8Q2_HUMAN]	11.95	3	3	42.58	5.5	0.60	7.76E-03
K7EK35	Signal transducer and activator of transcription OS=Homo sapiens GN=STAT5A PE=1 SV=1 - [K7EK35_HUMAN]	4.72	1	3	87.31	6.6	0.60	1.00E-02
Q9NZJ9	Diphosphoinositol polyphosphate phosphohydrolase 2 OS=Homo sapiens GN=NUDT4 PE=1 SV=2 - [NUDT4_HUMAN]	12.78	2	3	20.29	6.4	0.60	2.59E-04
A6NLG9	cDNA FLJ36740 fis, clone UTERU2013322, highly similar to Biglycan OS=Homo sapiens PE=2 SV=1 - [A6NLG9_HUMAN]	38.11	8	27	34.85	8.9	0.60	2.97E-03
C9JES6	Calcium/calmodulin-dependent protein kinase type 1 (Fragment) OS=Homo sapiens GN=CAMK1 PE=1 SV=1 - [C9JES6_HUMAN]	22.13	2	2	14.01	4.9	0.60	2.35E-03

B4DIT7	cDNA FLJ58187, highly similar to Protein-glutamine gamma-glutamyltransferase 2(EC 2.3.2.13) OS=Homo sapiens PE=2 SV=1 - [B4DIT7_HUMAN]	16.34	9	20	68.61	5.3	0.60	1.96E-03
O43491	Band 4.1-like protein 2 OS=Homo sapiens GN=EPB41L2 PE=1 SV=1 - [E41L2_HUMAN]	30.05	20	38	112.52	5.4	0.60	3.98E-04
B7Z650	cDNA FLJ58685, highly similar to Homo sapiens echinoderm microtubule associated protein like 1 (EML1), transcript variant 1, mRNA OS=Homo sapiens PE=2 SV=1 - [B7Z650_HUMAN]	1.12	1	1	88.70	8.0	0.60	1.29E-03
Q969Z3	Mitochondrial amidoxime reducing component 2 OS=Homo sapiens GN=MARC2 PE=1 SV=1 - [MARC2_HUMAN]	23.58	7	26	38.00	9.2	0.60	1.77E-03
P13010	X-ray repair cross-complementing protein 5 OS=Homo sapiens GN=XRCC5 PE=1 SV=3 - [XRCC5_HUMAN]	44.54	26	75	82.65	5.8	0.60	2.55E-03
B2RA94	cDNA, FLJ94779 OS=Homo sapiens PE=2 SV=1 - [B2RA94_HUMAN]	7.96	3	4	45.71	8.7	0.60	5.70E-03
B4DF70	cDNA FLJ60461, highly similar to Peroxiredoxin-2 (EC 1.11.1.15) OS=Homo sapiens PE=2 SV=1 - [B4DF70_HUMAN]	43.72	7	32	20.09	8.8	0.60	1.23E-04
P23141	Liver carboxylesterase 1 OS=Homo sapiens GN=CES1 PE=1 SV=2 - [EST1_HUMAN]	28.04	13	21	62.48	6.6	0.60	1.12E-03
H7C272	Checkpoint protein HUS1 (Fragment) OS=Homo sapiens GN=HUS1 PE=1 SV=1 - [H7C272_HUMAN]	10.48	1	1	11.79	9.7	0.60	2.49E-02
Q9UBG0	C-type mannose receptor 2 OS=Homo sapiens GN=MRC2 PE=1 SV=2 - [MRC2_HUMAN]	9.06	12	16	166.57	5.8	0.61	2.42E-03
P10768	S-formylglutathione hydrolase OS=Homo sapiens GN=ESD PE=1 SV=2 - [ESTD_HUMAN]	12.06	3	3	31.44	7.0	0.61	2.00E-02

Q96KK5	Histone H2A type 1-H OS=Homo sapiens GN=HIST1H2AH PE=1 SV=3 - [H2A1H_HUMAN]	38.28	1	61	13.90	10.9	0.61	9.20E-04
P00568	Adenylate kinase isoenzyme 1 OS=Homo sapiens GN=AK1 PE=1 SV=3 - [KAD1_HUMAN]	41.24	7	11	21.62	8.6	0.61	5.33E-03
S4R3K4	Nuclear factor 1 (Fragment) OS=Homo sapiens GN=NFIA PE=1 SV=1 - [S4R3K4_HUMAN]	12.37	2	3	20.95	6.9	0.61	5.32E-04
A0A1B0GTG3	Aminoacylase-1 OS=Homo sapiens GN=ACY1 PE=4 SV=1 - [A0A1B0GTG3_HUMAN]	17.7	1	1	12.19	5.7	0.61	3.02E-03
P21333	Filamin-A OS=Homo sapiens GN=FLNA PE=1 SV=4 - [FLNA_HUMAN]	45.41	82	266	280.56	6.1	0.61	1.99E-04
Q9H1E5	Thioredoxin-related transmembrane protein 4 OS=Homo sapiens GN=TMX4 PE=1 SV=1 - [TMX4_HUMAN]	18.05	6	12	38.93	4.4	0.61	7.15E-04
G3V144	SH3 and PX domain-containing protein 2B OS=Homo sapiens GN=SH3PXD2B PE=1 SV=1 - [G3V144_HUMAN]	4.42	2	2	49.14	7.2	0.61	1.06E-03
F8W0M9	Secernin-3 (Fragment) OS=Homo sapiens GN=SCRN3 PE=1 SV=1 - [F8W0M9_HUMAN]	5.26	1	1	23.59	4.8	0.61	5.69E-04
P42773	Cyclin-dependent kinase 4 inhibitor C OS=Homo sapiens GN=CDKN2C PE=1 SV=1 - [CDN2C_HUMAN]	6.55	1	2	18.12	6.5	0.61	1.62E-02
B1AKG0	Complement factor H-related protein 1 OS=Homo sapiens GN=CFHR1 PE=1 SV=1 - [B1AKG0_HUMAN]	12.55	2	4	30.84	7.8	0.61	4.16E-03
A0A0S2Z377	Annexin (Fragment) OS=Homo sapiens GN=ANXA6 PE=2 SV=1 - [A0A0S2Z377_HUMAN]	60.27	1	153	75.23	5.7	0.61	1.20E-03

P07355	Annexin A2 OS=Homo sapiens GN=ANXA2 PE=1 SV=2 - [ANXA2_HUMAN]	61.65	21	110	38.58	7.8	0.61	8.71E-04
K9J968	GDNF family receptor alpha-3 variant OS=Homo sapiens GN=GDNFR3 PE=2 SV=1 - [K9J968_HUMAN]	5.15	1	1	40.90	7.7	0.61	2.20E-02
A0A0C4DG09	Cell adhesion molecule 3 (Fragment) OS=Homo sapiens GN=CADM3 PE=1 SV=1 - [A0A0C4DG09_HUMAN]	7.69	1	2	25.64	6.4	0.61	9.69E-03
B2RTX2	Palladin, cytoskeletal associated protein OS=Homo sapiens GN=PALLD PE=2 SV=1 - [B2RTX2_HUMAN]	2.89	3	4	121.97	6.9	0.61	3.41E-03
K7ER90	Eukaryotic translation initiation factor 3 subunit G (Fragment) OS=Homo sapiens GN=EIF3G PE=1 SV=1 - [K7ER90_HUMAN]	9.25	2	2	25.43	5.5	0.61	7.81E-04
P19075	Tetraspanin-8 OS=Homo sapiens GN=TSPAN8 PE=1 SV=1 - [TSN8_HUMAN]	10.13	2	2	26.03	5.6	0.61	9.19E-04
Q9NZN3	EH domain-containing protein 3 OS=Homo sapiens GN=EHD3 PE=1 SV=2 - [EHD3_HUMAN]	18.69	1	16	60.85	6.6	0.61	9.64E-03
Q53FP0	Pyridoxine 5'-phosphate oxidase variant (Fragment) OS=Homo sapiens PE=2 SV=1 - [Q53FP0_HUMAN]	12.64	2	3	29.90	7.4	0.61	9.70E-03
A0A0A0MSB3	cGMP-dependent protein kinase 1 OS=Homo sapiens GN=PRKG1 PE=1 SV=1 - [A0A0A0MSB3_HUMAN]	1.96	1	1	51.87	5.6	0.61	7.80E-03
G4V2I8	Anion exchange protein OS=Homo sapiens PE=2 SV=1 - [G4V2I8_HUMAN]	22.72	18	48	101.68	5.2	0.61	7.16E-04
B2R6K4	cDNA, FLJ92996, highly similar to Homo sapiens guanine nucleotide binding protein (G protein), beta polypeptide 1 (GNB1), mRNA OS=Homo sapiens PE=2 SV=1 - [B2R6K4_HUMAN]	39.71	5	30	37.28	6.2	0.61	1.82E-02

X6RCK5	Dynactin subunit 3 (Fragment) OS=Homo sapiens GN=DCTN3 PE=1 SV=1 - [X6RCK5_HUMAN]	31.13	4	8	17.29	6.6	0.61	2.42E-03
A6NFY4	Nuclear envelope integral membrane protein 2 OS=Homo sapiens GN=NEMP2 PE=2 SV=3 - [NEMP2_HUMAN]	1.92	1	2	48.63	8.3	0.61	5.26E-03
Q8N5Y3	GYG1 protein (Fragment) OS=Homo sapiens GN=GYG1 PE=2 SV=2 - [Q8N5Y3_HUMAN]	8.46	2	3	37.22	5.4	0.61	3.20E-03
B2RAH7	cDNA, FLJ94921, highly similar to Homo sapiens prolyl endopeptidase (PREP), mRNA OS=Homo sapiens PE=2 SV=1 - [B2RAH7_HUMAN]	11.41	6	9	80.68	5.9	0.61	6.99E-04
P14543	Nidogen-1 OS=Homo sapiens GN=NID1 PE=1 SV=3 - [NID1_HUMAN]	6.09	5	8	136.29	5.3	0.61	3.11E-04
P83110	Serine protease HTRA3 OS=Homo sapiens GN=HTRA3 PE=1 SV=2 - [HTRA3_HUMAN]	5.08	1	2	48.58	7.1	0.61	2.47E-03
B8ZZA2	Hyccin OS=Homo sapiens GN=FAM126A PE=1 SV=1 - [B8ZZA2_HUMAN]	9.65	1	1	13.00	4.7	0.61	3.76E-04
B3KU56	cDNA FLJ39220 fis, clone OCBBF2006859, highly similar to Fibrillin-3 OS=Homo sapiens PE=2 SV=1 - [B3KU56_HUMAN]	2.31	1	1	70.14	6.2	0.62	3.39E-03
Q16647	Prostacyclin synthase OS=Homo sapiens GN=PTGIS PE=1 SV=1 - [PTGIS_HUMAN]	18.8	7	17	57.07	7.3	0.62	4.68E-03
S4R3A2	Fatty acid-binding protein, heart OS=Homo sapiens GN=FABP3 PE=1 SV=1 - [S4R3A2_HUMAN]	39.33	3	5	10.16	9.0	0.62	8.58E-03
P23396	40S ribosomal protein S3 OS=Homo sapiens GN=RPS3 PE=1 SV=2 - [RS3_HUMAN]	65.43	15	46	26.67	9.7	0.62	5.61E-05

Q5T123	SH3 domain-binding glutamic acid-rich-like protein 3 OS=Homo sapiens GN=SH3BGRL3 PE=1 SV=1 - [Q5T123_HUMAN]	28.41	3	4	9.37	9.4	0.62	6.28E-03
P30043	Flavin reductase (NADPH) OS=Homo sapiens GN=BLVRB PE=1 SV=3 - [BLVRB_HUMAN]	34.47	6	16	22.11	7.6	0.62	7.08E-04
Q6PJF2	IGK@ protein OS=Homo sapiens GN=IGK@ PE=1 SV=1 - [Q6PJF2_HUMAN]	42.98	1	52	25.50	6.6	0.62	7.95E-04
Q8TB45	DEP domain-containing mTOR-interacting protein OS=Homo sapiens GN=DEPTOR PE=1 SV=2 - [DPTOR_HUMAN]	4.4	2	4	46.26	8.1	0.62	1.59E-03
P05546	Heparin cofactor 2 OS=Homo sapiens GN=SERPIND1 PE=1 SV=3 - [HEP2_HUMAN]	5.01	3	4	57.03	6.9	0.62	1.55E-03
Q9NRV9	Heme-binding protein 1 OS=Homo sapiens GN=HEBP1 PE=1 SV=1 - [HEBP1_HUMAN]	13.76	2	2	21.08	5.8	0.62	2.60E-04
E7EQG2	Eukaryotic initiation factor 4A-II OS=Homo sapiens GN=EIF4A2 PE=1 SV=1 - [E7EQG2_HUMAN]	44.48	6	32	41.26	5.6	0.62	1.08E-02
Q96RF0	Sorting nexin-18 OS=Homo sapiens GN=SNX18 PE=1 SV=2 - [SNX18_HUMAN]	11.46	6	9	68.85	5.7	0.62	2.52E-03
Q5W0G0	Kin of IRRE-like protein 1 OS=Homo sapiens GN=KIRREL PE=1 SV=1 - [Q5W0G0_HUMAN]	5.95	2	2	63.06	5.9	0.62	3.14E-03
K7EKG4	Mitochondrial import receptor subunit TOM40 homolog (Fragment) OS=Homo sapiens GN=TOMM40 PE=1 SV=1 - [K7EKG4_HUMAN]	40.48	1	30	18.27	8.7	0.62	3.46E-03
Q96FJ2	Dynein light chain 2, cytoplasmic OS=Homo sapiens GN=DYNLL2 PE=1 SV=1 - [DYL2_HUMAN]	7.87	1	3	10.34	7.4	0.62	1.56E-03

B4DTA2	cDNA FLJ60148, highly similar to Homo sapiens heterogeneous nuclear ribonucleoprotein D-like (HNRPDL), transcript variant 2, mRNA OS=Homo sapiens PE=2 SV=1 - [B4DTA2_HUMAN]	16.61	3	18	30.20	8.7	0.62	1.32E-03
P02765	Alpha-2-HS-glycoprotein OS=Homo sapiens GN=AHSG PE=1 SV=1 - [FETUA_HUMAN]	11.17	5	10	39.30	5.7	0.62	1.53E-03
B4DVV1	cDNA FLJ51526, highly similar to Homo sapiens aldehyde dehydrogenase 16 family, member A1 (ALDH16A1), mRNA OS=Homo sapiens PE=2 SV=1 - [B4DVV1_HUMAN]	7.54	3	4	66.85	7.1	0.62	8.89E-03
Q53F27	Transmembrane protein 14C variant (Fragment) OS=Homo sapiens PE=2 SV=1 - [Q53F27_HUMAN]	24.11	2	4	11.57	9.9	0.62	1.17E-03
A6NFS0	Zinc finger protein 275 OS=Homo sapiens GN=ZNF275 PE=1 SV=1 - [A6NFS0_HUMAN]	2.43	1	1	37.02	8.4	0.62	2.14E-02
O95865	N(G),N(G)-dimethylarginine dimethylaminohydrolase 2 OS=Homo sapiens GN=DDAH2 PE=1 SV=1 - [DDAH2_HUMAN]	36.14	7	16	29.63	6.0	0.62	2.41E-03
F6RFD5	Destrin OS=Homo sapiens GN=DSTN PE=1 SV=1 - [F6RFD5_HUMAN]	44.44	6	20	15.39	8.6	0.62	4.52E-04
I3L3A7	Mapk-regulated corepressor-interacting protein 1 OS=Homo sapiens GN=MCRIP1 PE=1 SV=1 - [I3L3A7_HUMAN]	28.57	1	1	7.11	9.9	0.62	3.69E-03
Q10588	ADP-ribosyl cyclase/cyclic ADP-ribose hydrolase 2 OS=Homo sapiens GN=BST1 PE=1 SV=2 - [BST1_HUMAN]	25.16	6	7	35.70	7.8	0.62	1.28E-03
A0A0S2Z5I6	Optineurin isoform 3 OS=Homo sapiens GN=OPTN PE=2 SV=1 - [A0A0S2Z5I6_HUMAN]	7.94	2	2	28.30	5.3	0.62	1.01E-02
Q59GS8	Complement component 5 variant (Fragment) OS=Homo sapiens PE=2 SV=1 - [Q59GS8_HUMAN]	6.51	6	8	123.27	8.2	0.62	5.41E-04

P49756	RNA-binding protein 25 OS=Homo sapiens GN=RBM25 PE=1 SV=3 - [RBM25_HUMAN]	1.78	1	1	100.12	6.3	0.62	3.54E-02
B4E2Z6	Eukaryotic translation initiation factor 3 subunit C OS=Homo sapiens GN=EIF3C PE=2 SV=1 - [B4E2Z6_HUMAN]	19.52	11	20	90.48	5.7	0.62	6.44E-04
B4DES6	cDNA FLJ52441, highly similar to Zinc finger protein 512 OS=Homo sapiens PE=2 SV=1 - [B4DES6_HUMAN]	5.19	2	2	53.09	9.6	0.62	4.75E-03
A8K941	cDNA FLJ77618 OS=Homo sapiens PE=2 SV=1 - [A8K941_HUMAN]	1.55	1	1	79.36	7.8	0.63	1.83E-03
E9PGZ1	Caldesmon OS=Homo sapiens GN=CALD1 PE=1 SV=1 - [E9PGZ1_HUMAN]	16.98	9	14	61.67	6.1	0.63	6.10E-04
Q5T6V5	UPF0553 protein C9orf64 OS=Homo sapiens GN=C9orf64 PE=1 SV=1 - [CI064_HUMAN]	15.54	5	7	39.00	5.9	0.63	6.81E-04
P24593	Insulin-like growth factor-binding protein 5 OS=Homo sapiens GN=IGFBP5 PE=1 SV=1 - [IBP5_HUMAN]	2.94	1	3	30.55	8.2	0.63	2.08E-04
B4DMJ3	cDNA FLJ52994, highly similar to Guanidinoacetate N-methyltransferase (EC2.1.1.2) OS=Homo sapiens PE=2 SV=1 - [B4DMJ3_HUMAN]	17.36	1	1	12.96	6.5	0.63	1.14E-02
O60262	Guanine nucleotide-binding protein G(I)/G(S)/G(O) subunit gamma-7 OS=Homo sapiens GN=GNG7 PE=1 SV=1 - [GBG7_HUMAN]	44.12	2	3	7.52	8.5	0.63	3.91E-03
P43034	Platelet-activating factor acetylhydrolase IB subunit alpha OS=Homo sapiens GN=PAFAH1B1 PE=1 SV=2 - [LIS1_HUMAN]	16.59	5	9	46.61	7.4	0.63	6.20E-03
B7ZLW0	LPP protein OS=Homo sapiens GN=LPP PE=2 SV=1 - [B7ZLW0_HUMAN]	10.62	4	7	65.73	7.4	0.63	1.85E-03

B4DSV9	cDNA FLJ56632, moderately similar to Target of Nesh-SH3 OS=Homo sapiens PE=2 SV=1 - [B4DSV9_HUMAN]	1.26	1	1	113.36	9.6	0.63	1.24E-02
Q9H2D6	TRIO and F-actin-binding protein OS=Homo sapiens GN=TRIOBP PE=1 SV=3 - [TARA_HUMAN]	1.35	3	3	261.22	8.5	0.63	3.27E-02
A0A024R8V0	Septin 9, isoform CRA_a OS=Homo sapiens GN=SEPT9 PE=3 SV=1 - [A0A024R8V0_HUMAN]	20.77	10	17	63.59	8.4	0.63	2.99E-04
B4DIH5	COP9 signalosome complex subunit 2 OS=Homo sapiens GN=COPS2 PE=1 SV=1 - [B4DIH5_HUMAN]	20.32	7	9	44.20	5.9	0.63	7.70E-04
P19338	Nucleolin OS=Homo sapiens GN=NCL PE=1 SV=3 - [NUCL_HUMAN]	35.63	27	63	76.57	4.7	0.63	1.18E-04
B4DQF6	Eukaryotic translation initiation factor 3 subunit L OS=Homo sapiens GN=EIF3L PE=2 SV=1 - [B4DQF6_HUMAN]	6.97	4	7	62.65	6.2	0.63	1.71E-03
Q6KCM7	Calcium-binding mitochondrial carrier protein SCaMC-2 OS=Homo sapiens GN=SLC25A25 PE=1 SV=1 - [SCMC2_HUMAN]	18.55	5	15	52.63	8.4	0.63	8.83E-05
B7Z3I9	Delta-aminolevulinic acid dehydratase OS=Homo sapiens PE=2 SV=1 - [B7Z3I9_HUMAN]	13.74	3	3	34.50	7.2	0.63	3.27E-03
P04004	Vitronectin OS=Homo sapiens GN=VTN PE=1 SV=1 - [VTNC_HUMAN]	21.34	9	28	54.27	5.8	0.63	5.26E-04
A8KA05	Protein argonaute-3 OS=Homo sapiens GN=EIF2C3 PE=2 SV=1 - [A8KA05_HUMAN]	4.19	2	3	97.27	9.1	0.63	7.05E-03
Q15555	Microtubule-associated protein RP/EB family member 2 OS=Homo sapiens GN=MAPRE2 PE=1 SV=1 - [MARE2_HUMAN]	5.5	1	3	37.01	5.6	0.63	1.18E-02

Q66K79	Carboxypeptidase Z OS=Homo sapiens GN=CPZ PE=1 SV=2 - [CBPZ_HUMAN]	6.13	3	7	73.61	8.0	0.63	1.86E-02
Q8WVC2	40S ribosomal protein S21 OS=Homo sapiens GN=RPS21 PE=1 SV=1 - [Q8WVC2_HUMAN]	12.35	1	3	8.84	8.5	0.63	3.09E-03
B2R9T9	cDNA, FLJ94551 OS=Homo sapiens PE=2 SV=1 - [B2R9T9_HUMAN]	9.05	3	13	26.18	10.7	0.63	3.82E-03
Q13563	Polycystin-2 OS=Homo sapiens GN=PKD2 PE=1 SV=3 - [PKD2_HUMAN]	4.03	3	5	109.62	5.7	0.63	1.96E-03
Q14764	Major vault protein OS=Homo sapiens GN=MVP PE=1 SV=4 - [MVP_HUMAN]	31.13	22	48	99.27	5.5	0.63	1.49E-03
Q5QJ74	Tubulin-specific chaperone cofactor E-like protein OS=Homo sapiens GN=TBCEL PE=1 SV=2 - [TBCEL_HUMAN]	5.9	1	2	48.16	5.4	0.63	2.32E-02
B4DM44	cDNA FLJ57324, highly similar to Segment polarity protein dishevelled homolog DVL-2 OS=Homo sapiens PE=2 SV=1 - [B4DM44_HUMAN]	5.91	1	1	22.76	9.3	0.63	2.46E-02
Q86UD1	Out at first protein homolog OS=Homo sapiens GN=OAF PE=2 SV=1 - [OAF_HUMAN]	3.3	1	1	30.67	6.8	0.63	5.10E-03
Q16186	Proteasomal ubiquitin receptor ADRM1 OS=Homo sapiens GN=ADRM1 PE=1 SV=2 - [ADRM1_HUMAN]	6.14	2	4	42.13	5.1	0.63	2.19E-03
P20700	Lamin-B1 OS=Homo sapiens GN=LMNB1 PE=1 SV=2 - [LMNB1_HUMAN]	12.12	6	17	66.37	5.2	0.63	7.14E-03
H0YB18	Putative hydroxypyruvate isomerase (Fragment) OS=Homo sapiens GN=HYI PE=1 SV=1 - [H0YB18_HUMAN]	23.6	2	3	17.69	5.5	0.63	3.10E-02

Q86T13	C-type lectin domain family 14 member A OS=Homo sapiens GN=CLEC14A PE=1 SV=1 - [CLC14_HUMAN]	4.9	1	2	51.60	6.4	0.63	7.04E-04
X6RDA4	Paraspeckle component 1 (Fragment) OS=Homo sapiens GN=PSPC1 PE=1 SV=1 - [X6RDA4_HUMAN]	4.84	1	1	27.26	5.5	0.63	1.36E-02
Q9Y680	Peptidyl-prolyl cis-trans isomerase FKBP7 OS=Homo sapiens GN=FKBP7 PE=1 SV=1 - [FKBP7_HUMAN]	20.85	5	8	29.99	6.5	0.63	2.12E-03
A0A087X142	Septin-8 OS=Homo sapiens GN=SEPT8 PE=1 SV=1 - [A0A087X142_HUMAN]	18.31	3	16	49.33	6.2	0.63	5.87E-03
O75891	Cytosolic 10-formyltetrahydrofolate dehydrogenase OS=Homo sapiens GN=ALDH1L1 PE=1 SV=2 - [AL1L1_HUMAN]	4.88	3	7	98.77	5.9	0.64	2.95E-03
Q96P70	Importin-9 OS=Homo sapiens GN=IPO9 PE=1 SV=3 - [IPO9_HUMAN]	1.34	1	1	115.89	4.8	0.64	4.22E-03
A0A142CHG9	GO2-q chimeric G-protein OS=Homo sapiens PE=2 SV=1 - [A0A142CHG9_HUMAN]	26.84	2	44	40.21	6.0	0.64	9.34E-04
B7Z8Y6	cDNA FLJ58394, highly similar to Platelet endothelial cell adhesion molecule OS=Homo sapiens PE=2 SV=1 - [B7Z8Y6_HUMAN]	8.73	5	5	81.73	6.7	0.64	1.24E-03
Q8TD57	Dynein heavy chain 3, axonemal OS=Homo sapiens GN=DNAH3 PE=2 SV=1 - [DYH3_HUMAN]	0.15	1	1	470.47	6.4	0.64	1.65E-03
B4DGA3	cDNA FLJ55898, highly similar to Receptor-type tyrosine-protein phosphatase mu (EC 3.1.3.48) OS=Homo sapiens PE=2 SV=1 - [B4DGA3_HUMAN]	2.74	2	2	140.02	6.7	0.64	8.85E-03
P32969	60S ribosomal protein L9 OS=Homo sapiens GN=RPL9 PE=1 SV=1 - [RL9_HUMAN]	39.58	7	20	21.85	9.9	0.64	2.02E-03

I6L969	FBF1 protein (Fragment) OS=Homo sapiens GN=FBF1 PE=2 SV=1 - [I6L969_HUMAN]	0.85	1	1	91.90	6.9	0.64	1.10E-02
E9PMB9	Malic enzyme (Fragment) OS=Homo sapiens GN=ME3 PE=1 SV=8 - [E9PMB9_HUMAN]	15.86	6	11	64.24	8.3	0.64	6.14E-03
B4DEK9	Semaphorin-3B OS=Homo sapiens GN=SEMA3B PE=1 SV=1 - [B4DEK9_HUMAN]	3.45	1	1	45.01	9.7	0.64	4.15E-02
J3KTF8	Rho GDP-dissociation inhibitor 1 (Fragment) OS=Homo sapiens GN=ARHGDIA PE=1 SV=8 - [J3KTF8_HUMAN]	25.39	4	7	21.50	5.5	0.64	6.21E-03
V9HW68	Epididymis luminal protein 214 OS=Homo sapiens GN=HEL-214 PE=2 SV=1 - [V9HW68_HUMAN]	38.09	1	157	51.68	7.8	0.64	4.78E-03
B3KTE8	cDNA FLJ38146 fis, clone D9OST2003594, highly similar to Homo sapiens solute carrier family 25, member 30 (SLC25A30), mRNA OS=Homo sapiens PE=2 SV=1 - [B3KTE8_HUMAN]	4.17	1	1	24.19	9.2	0.64	3.27E-04
Q86W50	Methyltransferase-like protein 16 OS=Homo sapiens GN=METTL16 PE=1 SV=2 - [MET16_HUMAN]	4.63	1	1	63.58	7.9	0.64	1.64E-02
O15371	Eukaryotic translation initiation factor 3 subunit D OS=Homo sapiens GN=EIF3D PE=1 SV=1 - [EIF3D_HUMAN]	12.77	5	10	63.93	6.0	0.64	3.21E-03
P35237	Serpin B6 OS=Homo sapiens GN=SERPINB6 PE=1 SV=3 - [SPB6_HUMAN]	25.27	7	12	42.59	5.3	0.64	8.99E-04
Q86SR1	Polypeptide N-acetylgalactosaminyltransferase 10 OS=Homo sapiens GN=GALNT10 PE=1 SV=2 - [GLT10_HUMAN]	12.44	5	7	68.95	8.6	0.64	4.51E-04
B4DJI2	cDNA FLJ53342, highly similar to Granulins OS=Homo sapiens PE=2 SV=1 - [B4DJI2_HUMAN]	6.79	3	6	56.81	6.9	0.64	7.61E-03

Q9NR12	PDZ and LIM domain protein 7 OS=Homo sapiens GN=PDLIM7 PE=1 SV=1 - [PDLI7_HUMAN]	10.28	4	7	49.81	8.4	0.64	1.19E-03
H3BRS3	Telomere length regulation protein TEL2 homolog OS=Homo sapiens GN=TELO2 PE=1 SV=1 - [H3BRS3_HUMAN]	4.46	1	1	47.35	8.4	0.64	3.05E-04
E5RGT6	F-BAR and double SH3 domains protein 1 OS=Homo sapiens GN=FCHSD1 PE=1 SV=1 - [E5RGT6_HUMAN]	3.18	2	3	66.57	5.1	0.64	6.48E-04
Q6MZU1	Putative uncharacterized protein DKFZp686A1195 OS=Homo sapiens GN=DKFZp686A1195 PE=2 SV=1 - [Q6MZU1_HUMAN]	9.34	11	17	189.50	6.8	0.64	5.80E-04
Q86UX7	Fermitin family homolog 3 OS=Homo sapiens GN=FERMT3 PE=1 SV=1 - [URP2_HUMAN]	4.35	2	2	75.91	7.0	0.64	8.30E-03
B4DMT5	Eukaryotic translation initiation factor 3 subunit F OS=Homo sapiens GN=EIF3F PE=2 SV=1 - [B4DMT5_HUMAN]	31.27	8	14	33.22	5.6	0.64	1.04E-03
C9IZX9	GTPase-activating protein and VPS9 domain-containing protein 1 (Fragment) OS=Homo sapiens GN=GAPVD1 PE=1 SV=1 - [C9IZX9_HUMAN]	15.38	1	1	13.41	8.5	0.64	1.21E-02
B7Z1S8	cDNA FLJ56059, highly similar to Engulfment and cell motility protein 2 OS=Homo sapiens PE=2 SV=1 - [B7Z1S8_HUMAN]	3.54	1	2	62.08	6.3	0.64	8.80E-03
K7ERI9	Apolipoprotein C-I (Fragment) OS=Homo sapiens GN=APOC1 PE=1 SV=1 - [K7ERI9_HUMAN]	35.06	4	12	8.64	6.7	0.64	6.57E-04
K7ES11	(E3-independent) E2 ubiquitin-conjugating enzyme (Fragment) OS=Homo sapiens GN=UBE2O PE=1 SV=1 - [K7ES11_HUMAN]	2.6	1	1	88.81	5.0	0.64	2.56E-04
Q99536	Synaptic vesicle membrane protein VAT-1 homolog OS=Homo sapiens GN=VAT1 PE=1 SV=2 - [VAT1_HUMAN]	36.64	10	21	41.89	6.3	0.64	1.90E-02

P42025	Beta-centractin OS=Homo sapiens GN=ACTR1B PE=1 SV=1 - [ACTY_HUMAN]	21.81	3	13	42.27	6.4	0.64	1.12E-03
P10301	Ras-related protein R-Ras OS=Homo sapiens GN=RRAS PE=1 SV=1 - [RRAS_HUMAN]	29.36	4	11	23.47	6.9	0.64	1.88E-02
P60866	40S ribosomal protein S20 OS=Homo sapiens GN=RPS20 PE=1 SV=1 - [RS20_HUMAN]	25.21	3	10	13.36	9.9	0.64	5.19E-05
Q86WG5	Myotubularin-related protein 13 OS=Homo sapiens GN=SBF2 PE=1 SV=1 - [MTMRD_HUMAN]	2.87	4	4	208.33	7.1	0.64	9.28E-03
B4DPR2	cDNA FLJ50830, highly similar to Serum albumin OS=Homo sapiens PE=2 SV=1 - [B4DPR2_HUMAN]	75.33	1	869	59.54	7.2	0.64	3.65E-03
Q6ZQQ6	WD repeat-containing protein 87 OS=Homo sapiens GN=WDR87 PE=1 SV=3 - [WDR87_HUMAN]	0.28	1	1	332.97	7.3	0.64	3.85E-05
A0A024R8B1	TBC1 domain family, member 13, isoform CRA_b OS=Homo sapiens GN=TBC1D13 PE=4 SV=1 - [A0A024R8B1_HUMAN]	6.18	1	1	32.15	5.6	0.65	1.36E-02
B3KM48	cDNA FLJ10286 fis, clone HEMBB1001384, highly similar to COP9 signalosome complex subunit 4 OS=Homo sapiens PE=2 SV=1 - [B3KM48_HUMAN]	15.02	5	5	46.23	5.8	0.65	2.82E-03
Q13228	Selenium-binding protein 1 OS=Homo sapiens GN=SELENBP1 PE=1 SV=2 - [SBP1_HUMAN]	35.38	13	20	52.36	6.4	0.65	4.45E-04
Q8WXI4	Acyl-coenzyme A thioesterase 11 OS=Homo sapiens GN=ACOT11 PE=1 SV=1 - [ACO11_HUMAN]	3.13	1	1	68.45	8.4	0.65	9.20E-03
Q13347	Eukaryotic translation initiation factor 3 subunit I OS=Homo sapiens GN=EIF3I PE=1 SV=1 - [EIF3I_HUMAN]	17.23	6	9	36.48	5.6	0.65	1.56E-03

P04275	von Willebrand factor OS=Homo sapiens GN=VWF PE=1 SV=4 - [VWF_HUMAN]	10.59	1	36	309.06	5.5	0.65	6.81E-03
B2R5U1	cDNA, FLJ92620, highly similar to Homo sapiens staphylococcal nuclease domain containing 1 (SND1),mRNA OS=Homo sapiens PE=2 SV=1 - [B2R5U1_HUMAN]	29.38	20	33	99.61	7.0	0.65	4.43E-03
P25398	40S ribosomal protein S12 OS=Homo sapiens GN=RPS12 PE=1 SV=3 - [RS12_HUMAN]	53.79	7	21	14.51	7.2	0.65	1.54E-03
B4DW31	cDNA FLJ54186, highly similar to Nuclear receptor-binding protein OS=Homo sapiens PE=2 SV=1 - [B4DW31_HUMAN]	5.05	2	5	57.92	5.1	0.65	6.30E-04
A0A0A0MSK5	Torsin-1A-interacting protein 1 OS=Homo sapiens GN=TOR1AIP1 PE=1 SV=1 - [A0A0A0MSK5_HUMAN]	14.07	6	10	52.37	7.1	0.65	1.73E-03
Q8WUW1	Protein BRICK1 OS=Homo sapiens GN=BRK1 PE=1 SV=1 - [BRK1_HUMAN]	14.67	1	2	8.74	5.5	0.65	9.62E-03
B3KPK3	cDNA FLJ31895 fis, clone NT2RP7003680, highly similar to Kelch-like protein 4 OS=Homo sapiens PE=2 SV=1 - [B3KPK3_HUMAN]	2.37	1	1	80.22	6.9	0.65	2.58E-02
H0YN65	Carbohydrate sulfotransferase 14 OS=Homo sapiens GN=CHST14 PE=1 SV=1 - [H0YN65_HUMAN]	9.4	3	4	39.88	9.3	0.65	1.87E-03
J3QR95	E3 ubiquitin-protein ligase MIB2 (Fragment) OS=Homo sapiens GN=MIB2 PE=1 SV=1 - [J3QR95_HUMAN]	5.72	1	1	34.75	7.6	0.65	3.58E-02
P62750	60S ribosomal protein L23a OS=Homo sapiens GN=RPL23A PE=1 SV=1 - [RL23A_HUMAN]	31.41	5	13	17.68	10.4	0.65	7.46E-03
P60228	Eukaryotic translation initiation factor 3 subunit E OS=Homo sapiens GN=EIF3E PE=1 SV=1 - [EIF3E_HUMAN]	26.29	11	18	52.19	6.0	0.65	1.78E-04

Q96F85	CB1 cannabinoid receptor-interacting protein 1 OS=Homo sapiens GN=CNRIP1 PE=1 SV=1 - [CNRP1_HUMAN]	25	3	3	18.64	7.9	0.65	3.61E-04
A0A0B4J203	Uncharacterized protein OS=Homo sapiens PE=3 SV=1 - [A0A0B4J203_HUMAN]	3.18	2	2	94.64	5.0	0.65	4.90E-02
A0A024R5L7	Phosphatidylinositol binding clathrin assembly protein, isoform CRA_c OS=Homo sapiens GN=PICALM PE=4 SV=1 - [A0A024R5L7_HUMAN]	6.07	3	4	66.35	8.8	0.65	5.68E-03
B4DPX8	cDNA FLJ58776, highly similar to Nidogen-2 OS=Homo sapiens PE=2 SV=1 - [B4DPX8_HUMAN]	17.89	7	11	62.14	5.8	0.65	2.89E-04
O75531	Barrier-to-autointegration factor OS=Homo sapiens GN=BANF1 PE=1 SV=1 - [BAF_HUMAN]	57.3	4	17	10.05	6.1	0.65	7.50E-04
Q9BTV4	Transmembrane protein 43 OS=Homo sapiens GN=TMEM43 PE=1 SV=1 - [TMM43_HUMAN]	49.5	4	62	44.85	8.1	0.65	1.34E-02
B4DTY8	cDNA FLJ61587, highly similar to Integrin alpha-1 (Fragment) OS=Homo sapiens PE=2 SV=1 - [B4DTY8_HUMAN]	6.39	8	9	129.92	6.2	0.65	2.62E-02
P21399	Cytoplasmic aconitate hydratase OS=Homo sapiens GN=ACO1 PE=1 SV=3 - [ACOC_HUMAN]	13.27	10	13	98.34	6.7	0.65	6.04E-03
Q15435	Protein phosphatase 1 regulatory subunit 7 OS=Homo sapiens GN=PPP1R7 PE=1 SV=1 - [PP1R7_HUMAN]	10.83	3	3	41.54	4.9	0.65	8.83E-03
G3V1N2	HCG1745306, isoform CRA_a OS=Homo sapiens GN=HBA2 PE=1 SV=1 - [G3V1N2_HUMAN]	67.27	1	105	11.94	8.8	0.65	4.52E-04
B7Z4C3	cDNA FLJ50805, highly similar to Erythrocyte membrane protein band 4.2 OS=Homo sapiens PE=2 SV=1 - [B7Z4C3_HUMAN]	18.09	10	19	76.99	8.2	0.65	1.07E-03

B4DVZ0	cDNA FLJ50253, highly similar to Heat shock 70 kDa protein 12B OS=Homo sapiens PE=2 SV=1 - [B4DVZ0_HUMAN]	6.67	2	5	66.77	9.0	0.65	9.92E-04
A0A024RBH7	Thymopoietin, isoform CRA_a OS=Homo sapiens GN=TMPO PE=4 SV=1 - [A0A024RBH7_HUMAN]	16.23	2	7	38.71	9.2	0.65	3.29E-04
K7ES31	Eukaryotic translation initiation factor 3 subunit K OS=Homo sapiens GN=EIF3K PE=1 SV=1 - [K7ES31_HUMAN]	20.44	2	4	15.86	6.7	0.65	5.63E-03
B4DPP8	cDNA FLJ53075, highly similar to Kininogen-1 OS=Homo sapiens PE=2 SV=1 - [B4DPP8_HUMAN]	22.17	8	11	46.47	6.4	0.65	3.84E-03
A8K9D8	cDNA FLJ78129, highly similar to Homo sapiens reversion-inducing-cysteine-rich protein with kazal motifs (RECK), mRNA OS=Homo sapiens PE=2 SV=1 - [A8K9D8_HUMAN]	6.9	5	5	106.39	6.7	0.66	5.74E-03
B4DJ12	cDNA FLJ58355, highly similar to Tyrosine-protein phosphatase non-receptor type 23 (EC 3.1.3.48) OS=Homo sapiens PE=2 SV=1 - [B4DJ12_HUMAN]	7.02	5	7	139.24	6.6	0.66	6.16E-03
Q9H6T3	RNA polymerase II-associated protein 3 OS=Homo sapiens GN=RPAP3 PE=1 SV=2 - [RPAP3_HUMAN]	1.8	1	1	75.67	6.8	0.66	1.30E-02
P35241	Radixin OS=Homo sapiens GN=RDX PE=1 SV=1 - [RADI_HUMAN]	29.67	8	43	68.52	6.4	0.66	5.94E-04
B4DX05	cDNA FLJ55948, highly similar to HECT domain and RCC1-like domain-containing protein 5 OS=Homo sapiens PE=2 SV=1 - [B4DX05_HUMAN]	2.1	1	1	50.57	5.5	0.66	3.53E-03
P49675	Steroidogenic acute regulatory protein, mitochondrial OS=Homo sapiens GN=STAR PE=1 SV=2 - [STAR_HUMAN]	37.19	8	20	31.89	9.0	0.66	2.54E-04
Q9Y2V2	Calcium-regulated heat-stable protein 1 OS=Homo sapiens GN=CARHSP1 PE=1 SV=2 - [CHSP1_HUMAN]	10.88	1	10	15.88	8.2	0.66	7.64E-03

P04632	Calpain small subunit 1 OS=Homo sapiens GN=CAPNS1 PE=1 SV=1 - [CPNS1_HUMAN]	32.09	7	16	28.30	5.2	0.66	1.22E-03
Q99733	Nucleosome assembly protein 1-like 4 OS=Homo sapiens GN=NAP1L4 PE=1 SV=1 - [NP1L4_HUMAN]	22.93	7	11	42.80	4.7	0.66	5.38E-03
D6RDN9	[Pyruvate dehydrogenase (acetyl-transferring)] kinase isozyme 2, mitochondrial (Fragment) OS=Homo sapiens GN=PDK2 PE=1 SV=1 - [D6RDN9_HUMAN]	23.55	3	8	29.37	5.8	0.66	4.87E-02
Q9UN75	Protocadherin alpha-12 OS=Homo sapiens GN=PCDHA12 PE=2 SV=1 - [PCDAC_HUMAN]	1.49	1	1	101.59	5.3	0.66	3.71E-03
Q49A62	AMT protein OS=Homo sapiens GN=AMT PE=2 SV=1 - [Q49A62_HUMAN]	14.81	3	4	28.83	8.7	0.66	1.26E-02
Q99969	Retinoic acid receptor responder protein 2 OS=Homo sapiens GN=RARRES2 PE=1 SV=1 - [RARR2_HUMAN]	14.11	2	4	18.61	9.1	0.66	5.04E-03
A4FVA8	DFNA5 protein (Fragment) OS=Homo sapiens GN=DFNA5 PE=2 SV=1 - [A4FVA8_HUMAN]	5.26	1	2	47.70	5.2	0.66	1.07E-02
G3V5L7	Protein NDRG2 (Fragment) OS=Homo sapiens GN=NDRG2 PE=1 SV=1 - [G3V5L7_HUMAN]	35.32	4	5	24.12	4.8	0.66	1.34E-03
B3KWQ6	cDNA FLJ43599 fis, clone SMINT2017781, highly similar to PERIPHERIN OS=Homo sapiens PE=2 SV=1 - [B3KWQ6_HUMAN]	10.43	2	16	53.65	5.5	0.66	1.56E-02
F8W8H5	Ras-related protein Rab-24 OS=Homo sapiens GN=RAB24 PE=1 SV=1 - [F8W8H5_HUMAN]	5.17	1	1	19.66	5.4	0.66	7.77E-03
Q96CA8	CDC2L2 protein (Fragment) OS=Homo sapiens GN=CDC2L2 PE=2 SV=1 - [Q96CA8_HUMAN]	2.16	1	1	52.37	5.1	0.66	3.34E-02

B4E2V5	cDNA FLJ52062, highly similar to Erythrocyte band 7 integral membrane protein OS=Homo sapiens PE=2 SV=1 - [B4E2V5_HUMAN]	33.33	7	14	25.95	7.4	0.66	7.03E-03
P36871	Phosphoglucomutase-1 OS=Homo sapiens GN=PGM1 PE=1 SV=3 - [PGM1_HUMAN]	32.74	15	32	61.41	6.8	0.66	1.52E-04
A0A087WTT6	40S ribosomal protein S29 OS=Homo sapiens GN=RPS29 PE=1 SV=1 - [A0A087WTT6_HUMAN]	13.21	1	2	6.11	9.1	0.66	3.27E-03
P62070	Ras-related protein R-Ras2 OS=Homo sapiens GN=RRAS2 PE=1 SV=1 - [RRAS2_HUMAN]	18.63	2	7	23.38	6.0	0.66	6.54E-03
Q86TY5	Galectin OS=Homo sapiens PE=2 SV=1 - [Q86TY5_HUMAN]	52.89	6	12	13.89	9.2	0.66	2.45E-03
O14828	Secretory carrier-associated membrane protein 3 OS=Homo sapiens GN=SCAMP3 PE=1 SV=3 - [SCAM3_HUMAN]	5.76	2	4	38.26	7.6	0.66	3.27E-02
O75369	Filamin-B OS=Homo sapiens GN=FLNB PE=1 SV=2 - [FLNB_HUMAN]	26.59	44	95	277.99	5.7	0.66	5.36E-03
A8K2Q6	Peptidyl-prolyl cis-trans isomerase OS=Homo sapiens PE=2 SV=1 - [A8K2Q6_HUMAN]	9.91	2	3	22.70	8.4	0.66	4.29E-02
B8ZZZ7	DNA polymerase-transactivated protein 6, isoform CRA_b OS=Homo sapiens GN=SPATS2L PE=1 SV=1 - [B8ZZZ7_HUMAN]	4.22	2	2	54.91	9.7	0.66	2.30E-03
Q04721	Neurogenic locus notch homolog protein 2 OS=Homo sapiens GN=NOTCH2 PE=1 SV=3 - [NOTC2_HUMAN]	0.89	2	2	265.23	5.1	0.66	6.98E-03
P37837	Transaldolase OS=Homo sapiens GN=TALDO1 PE=1 SV=2 - [TALDO_HUMAN]	36.5	13	29	37.52	6.8	0.66	3.52E-03

Q9P000	COMM domain-containing protein 9 OS=Homo sapiens GN=COMMD9 PE=1 SV=2 - [COMD9_HUMAN]	11.62	1	1	21.81	5.9	0.67	4.56E-02
P43652	Afamin OS=Homo sapiens GN=AFM PE=1 SV=1 - [AFAM_HUMAN]	5.34	4	4	69.02	5.9	0.67	8.48E-04
B3KWX2	cDNA FLJ44075 fis, clone TESTI4038339, highly similar to Disco-interacting protein 2 homolog C OS=Homo sapiens PE=2 SV=1 - [B3KWX2_HUMAN]	1.37	1	1	71.31	7.6	0.67	3.44E-03
S6AWF4	IgG L chain OS=Homo sapiens PE=2 SV=1 - [S6AWF4_HUMAN]	23.89	1	16	19.83	7.1	0.67	8.55E-04
A0A0D9SG72	Syntaxin-binding protein 1 OS=Homo sapiens GN=STXBP1 PE=1 SV=2 - [A0A0D9SG72_HUMAN]	21.72	7	11	66.12	6.8	0.67	1.79E-03
A0A0K0Q2Z1	Serpin peptidase inhibitor clade C member 1 OS=Homo sapiens GN=SERPINC1 PE=2 SV=1 - [A0A0K0Q2Z1_HUMAN]	34.48	14	28	52.51	6.7	0.67	1.59E-03
K7EMP1	Coiled-coil and C2 domain-containing protein 1A (Fragment) OS=Homo sapiens GN=CC2D1A PE=1 SV=1 - [K7EMP1_HUMAN]	3.95	1	1	45.93	9.7	0.67	2.05E-02
P53618	Coatomer subunit beta OS=Homo sapiens GN=COPB1 PE=1 SV=3 - [COPB_HUMAN]	32.21	23	35	107.07	6.0	1.50	7.25E-04
Q9BW30	Tubulin polymerization-promoting protein family member 3 OS=Homo sapiens GN=TPPP3 PE=1 SV=1 - [TPPP3_HUMAN]	9.09	1	1	18.97	9.1	1.50	2.89E-03
Q96DP5	Methionyl-tRNA formyltransferase, mitochondrial OS=Homo sapiens GN=MTFMT PE=1 SV=2 - [FMT_HUMAN]	5.4	2	2	43.80	9.7	1.50	1.15E-04
O76031	ATP-dependent Clp protease ATP-binding subunit clpX-like, mitochondrial OS=Homo sapiens GN=CLPX PE=1 SV=2 - [CLPX_HUMAN]	35.23	20	42	69.18	7.6	1.50	6.06E-03

Q9BZQ6	ER degradation-enhancing alpha-mannosidase-like protein 3 OS=Homo sapiens GN=EDEM3 PE=1 SV=2 - [EDEM3_HUMAN]	9.12	7	11	104.60	4.9	1.50	1.66E-02
A0A0S2Z5K6	SIL1-like protein endoplasmic reticulum chaperone isoform 2 (Fragment) OS=Homo sapiens GN=SIL1 PE=2 SV=1 - [A0A0S2Z5K6_HUMAN]	9.82	3	6	44.49	5.4	1.50	7.09E-03
B3KUJ7	cDNA FLJ40064 fis, clone TESOP2000312, highly similar to LAS1-like protein OS=Homo sapiens PE=2 SV=1 - [B3KUJ7_HUMAN]	3.05	1	1	34.18	5.2	1.50	2.08E-02
P82914	28S ribosomal protein S15, mitochondrial OS=Homo sapiens GN=MRPS15 PE=1 SV=1 - [RT15_HUMAN]	33.07	10	20	29.82	10.5	1.50	7.14E-03
Q5EBM0	UMP-CMP kinase 2, mitochondrial OS=Homo sapiens GN=CMPK2 PE=1 SV=3 - [CMPK2_HUMAN]	21.83	8	10	49.42	7.0	1.50	1.37E-02
Q9P0I2	ER membrane protein complex subunit 3 OS=Homo sapiens GN=EMC3 PE=1 SV=3 - [EMC3_HUMAN]	20.31	5	8	29.93	6.8	1.50	5.27E-03
A0A024RD07	Trinucleotide repeat containing 5, isoform CRA_c OS=Homo sapiens GN=TNRC5 PE=4 SV=1 - [A0A024RD07_HUMAN]	19.78	6	12	30.75	5.5	1.51	1.36E-02
Q9BPW8	Protein NipSnap homolog 1 OS=Homo sapiens GN=NIPSNAP1 PE=1 SV=1 - [NIPS1_HUMAN]	44.72	10	33	33.29	9.3	1.51	1.65E-02
Q8TBT6	Putative uncharacterized protein (Fragment) OS=Homo sapiens PE=2 SV=1 - [Q8TBT6_HUMAN]	27.3	6	69	34.47	8.9	1.51	1.75E-02
P11177	Pyruvate dehydrogenase E1 component subunit beta, mitochondrial OS=Homo sapiens GN=PDHB PE=1 SV=3 - [ODPB_HUMAN]	52.92	14	79	39.21	6.7	1.51	3.25E-03
O94919	Endonuclease domain-containing 1 protein OS=Homo sapiens GN=ENDOD1 PE=1 SV=2 - [ENDD1_HUMAN]	10.2	4	13	54.98	5.7	1.51	3.89E-03

Q9Y617	Phosphoserine aminotransferase OS=Homo sapiens GN=PSAT1 PE=1 SV=2 - [SERC_HUMAN]	30.27	10	15	40.40	7.7	1.51	1.15E-02
A8KA41	cDNA FLJ75060, highly similar to Homo sapiens 2'-5'-oligoadenylate synthetase 2, 69/71kDa, mRNA OS=Homo sapiens PE=2 SV=1 - [A8KA41_HUMAN]	17.47	13	26	78.77	7.9	1.51	4.42E-03
E9PKG6	Nucleobindin-2 OS=Homo sapiens GN=NUCB2 PE=1 SV=2 - [E9PKG6_HUMAN]	41.42	11	26	40.34	5.2	1.51	1.45E-04
H0YG77	Transmembrane protein 120B (Fragment) OS=Homo sapiens GN=TMEM120B PE=1 SV=1 - [H0YG77_HUMAN]	5.67	1	2	29.02	9.3	1.51	1.41E-02
J3KSH0	StAR-related lipid transfer protein 3 (Fragment) OS=Homo sapiens GN=STARD3 PE=1 SV=1 - [J3KSH0_HUMAN]	10.81	1	1	12.81	9.5	1.51	1.25E-02
E7D7X9	Pyrroline-5-carboxylate reductase OS=Homo sapiens PE=2 SV=1 - [E7D7X9_HUMAN]	42.95	8	23	33.37	7.6	1.51	2.95E-03
Q9UES0	SNARE protein Ykt6 (Fragment) OS=Homo sapiens PE=2 SV=1 - [Q9UES0_HUMAN]	20.94	5	7	21.59	6.6	1.51	2.75E-03
Q14554	Protein disulfide-isomerase A5 OS=Homo sapiens GN=PDIA5 PE=1 SV=1 - [PDIA5_HUMAN]	26.59	12	25	59.56	7.9	1.51	1.21E-02
E9PGN6	rRNA methyltransferase 2, mitochondrial OS=Homo sapiens GN=MRM2 PE=1 SV=1 - [E9PGN6_HUMAN]	11.18	2	2	16.92	8.3	1.51	6.41E-05
O00116	Alkyldihydroxyacetonephosphate synthase, peroxisomal OS=Homo sapiens GN=AGPS PE=1 SV=1 - [ADAS_HUMAN]	34.04	15	36	72.87	7.3	1.51	5.68E-03
P12532	Creatine kinase U-type, mitochondrial OS=Homo sapiens GN=CKMT1A PE=1 SV=1 - [KCRU_HUMAN]	23.5	5	23	47.01	8.3	1.51	5.20E-03

Q5GFL6	von Willebrand factor A domain-containing protein 2 OS=Homo sapiens GN=VWA2 PE=1 SV=1 - [VWA2_HUMAN]	2.52	1	1	81.96	8.0	1.51	1.04E-02
Q15813	Tubulin-specific chaperone E OS=Homo sapiens GN=TBCE PE=1 SV=1 - [TBCE_HUMAN]	1.71	1	1	59.31	6.8	1.52	8.08E-03
B4DL56	Polypeptide N-acetylgalactosaminyltransferase OS=Homo sapiens PE=2 SV=1 - [B4DL56_HUMAN]	10.28	4	4	68.83	8.3	1.52	2.14E-02
Q6AW98	Putative uncharacterized protein DKFZp686P13170 OS=Homo sapiens GN=DKFZp686P13170 PE=2 SV=1 - [Q6AW98_HUMAN]	1.74	1	1	46.00	9.9	1.52	9.82E-03
Q9H490	Phosphatidylinositol glycan anchor biosynthesis class U protein OS=Homo sapiens GN=PIGU PE=1 SV=3 - [PIGU_HUMAN]	7.13	3	4	50.02	7.7	1.52	4.91E-03
P13073	Cytochrome c oxidase subunit 4 isoform 1, mitochondrial OS=Homo sapiens GN=COX4I1 PE=1 SV=1 - [COX41_HUMAN]	36.09	7	48	19.56	9.5	1.52	4.37E-03
P49411	Elongation factor Tu, mitochondrial OS=Homo sapiens GN=TUFM PE=1 SV=2 - [EFTU_HUMAN]	63.27	29	251	49.51	7.6	1.52	2.10E-03
Q96CU9	FAD-dependent oxidoreductase domain-containing protein 1 OS=Homo sapiens GN=FOXRED1 PE=1 SV=2 - [FXRD1_HUMAN]	21.19	7	12	53.78	7.8	1.52	3.87E-03
O00411	DNA-directed RNA polymerase, mitochondrial OS=Homo sapiens GN=POLRMT PE=1 SV=2 - [RPOM_HUMAN]	11.79	11	17	138.53	9.0	1.52	3.34E-04
Q6QN92	Mitochondrial glycine cleavage system H-protein (Fragment) OS=Homo sapiens PE=2 SV=1 - [Q6QN92_HUMAN]	24	2	13	13.80	4.3	1.52	2.88E-03
H7BXL1	Transmembrane protein 41A OS=Homo sapiens GN=TMEM41A PE=1 SV=1 - [H7BXL1_HUMAN]	23.91	2	2	10.43	9.0	1.52	1.31E-03

B4DWN1	cDNA FLJ52285, highly similar to Vesicular integral-membrane protein VIP36 OS=Homo sapiens PE=2 SV=1 - [B4DWN1_HUMAN]	61.4	14	46	32.56	6.5	1.52	1.37E-02
P30084	Enoyl-CoA hydratase, mitochondrial OS=Homo sapiens GN=ECHS1 PE=1 SV=4 - [ECHM_HUMAN]	75.86	19	156	31.37	8.1	1.52	3.56E-03
Q99757	Thioredoxin, mitochondrial OS=Homo sapiens GN=TXN2 PE=1 SV=2 - [THIOM_HUMAN]	36.75	3	18	18.37	8.3	1.52	5.11E-03
Q9BZF1	Oxysterol-binding protein-related protein 8 OS=Homo sapiens GN=OSBPL8 PE=1 SV=3 - [OSBL8_HUMAN]	9.45	8	10	101.13	7.0	1.52	1.31E-03
Q8TEM1	Nuclear pore membrane glycoprotein 210 OS=Homo sapiens GN=NUP210 PE=1 SV=3 - [PO210_HUMAN]	13.14	19	30	204.98	6.8	1.53	1.39E-02
B3KPG6	cDNA FLJ31769 fis, clone NT2RI2007956, highly similar to Chondroitin sulfate glucuronyltransferase (EC 2.4.1.226) OS=Homo sapiens PE=2 SV=1 - [B3KPG6_HUMAN]	4.19	2	2	85.11	7.9	1.53	1.18E-02
B4DHG3	cDNA FLJ55329, highly similar to Procollagen-lysine,2-oxoglutarate5-dioxygenase 2 (EC 1.14.11.4) OS=Homo sapiens PE=2 SV=1 - [B4DHG3_HUMAN]	13.37	8	8	81.09	6.7	1.53	1.14E-02
A0A024R6B4	Ectonucleoside triphosphate diphosphohydrolase 5, isoform CRA_c OS=Homo sapiens GN=ENTPD5 PE=3 SV=1 - [A0A024R6B4_HUMAN]	12.59	4	5	44.97	6.1	1.53	5.86E-04
A0A024R8L7	Acyl-coenzyme A oxidase OS=Homo sapiens GN=ACOX1 PE=3 SV=1 - [A0A024R8L7_HUMAN]	29.85	14	23	74.64	7.6	1.53	3.40E-02
Q8IXM3	39S ribosomal protein L41, mitochondrial OS=Homo sapiens GN=MRPL41 PE=1 SV=1 - [RM41_HUMAN]	38.69	5	9	15.37	9.6	1.53	3.78E-02
A8KAK1	cDNA FLJ77398, highly similar to Homo sapiens UDP-glucose ceramide glucosyltransferase-like 1, transcript variant 2, mRNA OS=Homo sapiens PE=2 SV=1 - [A8KAK1_HUMAN]	30.24	34	77	174.90	5.5	1.53	7.27E-03

B7ZLX4	PPP1R9A protein OS=Homo sapiens GN=PPP1R9A PE=2 SV=1 - [B7ZLX4_HUMAN]	0.92	1	1	122.35	5.1	1.53	6.74E-03
Q96AA3	Protein RFT1 homolog OS=Homo sapiens GN=RFT1 PE=1 SV=1 - [RFT1_HUMAN]	6.28	4	4	60.30	8.9	1.53	2.66E-02
Q9NX00	Transmembrane protein 160 OS=Homo sapiens GN=TMEM160 PE=1 SV=1 - [TM160_HUMAN]	10.64	2	3	19.65	8.0	1.53	2.98E-03
Q659F8	Putative uncharacterized protein DKFZp434F152 (Fragment) OS=Homo sapiens GN=DKFZp434F152 PE=2 SV=1 - [Q659F8_HUMAN]	4.89	1	1	23.86	9.0	1.53	2.00E-02
K7ERA3	Synaptonemal complex protein SC65 (Fragment) OS=Homo sapiens GN=P3H4 PE=1 SV=1 - [K7ERA3_HUMAN]	9.05	2	2	25.97	5.5	1.53	1.22E-02
E9PIE4	Mitochondrial carrier homolog 2 (Fragment) OS=Homo sapiens GN=MTCH2 PE=1 SV=8 - [E9PIE4_HUMAN]	34.35	1	37	28.53	7.6	1.53	4.97E-03
Q6ZNC8	Lysophospholipid acyltransferase 1 OS=Homo sapiens GN=MBOAT1 PE=1 SV=1 - [MBOA1_HUMAN]	2.83	1	1	56.52	9.2	1.53	4.32E-02
Q9H4N9	Clone CDABP0042 mRNA sequence OS=Homo sapiens PE=2 SV=1 - [Q9H4N9_HUMAN]	9.69	3	4	39.22	7.7	1.53	5.27E-03
F5GYW6	Exportin-T (Fragment) OS=Homo sapiens GN=XPOT PE=1 SV=1 - [F5GYW6_HUMAN]	15	1	2	6.92	4.7	1.53	1.07E-02
Q04837	Single-stranded DNA-binding protein, mitochondrial OS=Homo sapiens GN=SSBP1 PE=1 SV=1 - [SSBP_HUMAN]	55.41	7	78	17.25	9.6	1.53	1.88E-03
Q9H2W6	39S ribosomal protein L46, mitochondrial OS=Homo sapiens GN=MRPL46 PE=1 SV=1 - [RM46_HUMAN]	36.92	8	13	31.69	7.0	1.53	7.89E-03

B4DYF9	cDNA FLJ57669, highly similar to SWI/SNF-related matrix-associatedactin-dependent regulator of chromatin subfamily C member 1 OS=Homo sapiens PE=2 SV=1 - [B4DYF9_HUMAN]	1.8	1	1	66.97	5.3	1.53	2.15E-02
H3BMA1	Mesothelin (Fragment) OS=Homo sapiens GN=MSLN PE=1 SV=2 - [H3BMA1_HUMAN]	13.47	4	8	37.65	6.0	1.53	2.68E-02
O95202	LETM1 and EF-hand domain-containing protein 1, mitochondrial OS=Homo sapiens GN=LETM1 PE=1 SV=1 - [LETM1_HUMAN]	44.25	30	104	83.30	6.7	1.54	3.60E-03
A0A087WVM4	Monofunctional C1-tetrahydrofolate synthase, mitochondrial OS=Homo sapiens GN=MTHFD1L PE=1 SV=1 - [A0A087WVM4_HUMAN]	25.52	19	33	99.25	7.3	1.54	1.06E-03
A0A024R210	Interferon induced transmembrane protein 1 (9-27), isoform CRA_a OS=Homo sapiens GN=IFITM1 PE=4 SV=1 - [A0A024R210_HUMAN]	27.2	2	9	13.93	7.9	1.54	3.77E-02
Q8N5M1	ATP synthase mitochondrial F1 complex assembly factor 2 OS=Homo sapiens GN=ATPAF2 PE=1 SV=1 - [ATPF2_HUMAN]	21.45	5	10	32.75	7.1	1.54	1.57E-04
G3V2V6	V-type proton ATPase subunit D OS=Homo sapiens GN=ATP6V1D PE=1 SV=1 - [G3V2V6_HUMAN]	7.43	1	1	17.41	9.5	1.54	8.22E-04
Q14249	Endonuclease G, mitochondrial OS=Homo sapiens GN=ENDOG PE=1 SV=4 - [NUCG_HUMAN]	7.74	2	2	32.60	9.5	1.54	3.16E-03
Q9BXT2	Voltage-dependent calcium channel gamma-6 subunit OS=Homo sapiens GN=CACNG6 PE=2 SV=1 - [CCG6_HUMAN]	2.69	1	1	28.11	9.2	1.54	3.35E-03
O15270	Serine palmitoyltransferase 2 OS=Homo sapiens GN=SPTLC2 PE=1 SV=1 - [SPTC2_HUMAN]	19.4	8	12	62.88	7.8	1.54	1.68E-02
A6PVJ3	ERGIC and golgi 3 (Fragment) OS=Homo sapiens GN=ERGIC3 PE=4 SV=1 - [A6PVJ3_HUMAN]	28	7	15	36.83	5.3	1.54	2.56E-02

P35270	Sepiapterin reductase OS=Homo sapiens GN=SPR PE=1 SV=1 - [SPRE_HUMAN]	23.37	6	12	28.03	8.0	1.54	1.46E-04
P09669	Cytochrome c oxidase subunit 6C OS=Homo sapiens GN=COX6C PE=1 SV=2 - [COX6C_HUMAN]	52	7	30	8.78	10.4	1.54	5.97E-03
H7C0R6	THUMP domain-containing protein 3 (Fragment) OS=Homo sapiens GN=THUMPD3 PE=1 SV=1 - [H7C0R6_HUMAN]	4.74	1	2	25.92	5.4	1.54	1.55E-03
P49789	Bis(5'-adenosyl)-triphosphatase OS=Homo sapiens GN=FHIT PE=1 SV=3 - [FHIT_HUMAN]	27.89	3	5	16.85	7.1	1.54	6.88E-04
B4DQ79	Tyrosine-protein kinase OS=Homo sapiens PE=2 SV=1 - [B4DQ79_HUMAN]	20.33	5	12	48.53	7.4	1.54	3.74E-02
B4DGC1	cDNA FLJ59877, highly similar to LAG1 longevity assurance homolog 5 OS=Homo sapiens PE=2 SV=1 - [B4DGC1_HUMAN]	3.59	1	1	22.23	9.2	1.54	4.61E-03
Q02338	D-beta-hydroxybutyrate dehydrogenase, mitochondrial OS=Homo sapiens GN=BDH1 PE=1 SV=3 - [BDH_HUMAN]	41.98	11	23	38.13	9.0	1.54	2.99E-03
Q96B77	Transmembrane protein 186 OS=Homo sapiens GN=TMEM186 PE=2 SV=1 - [TM186_HUMAN]	13.62	2	4	24.88	9.9	1.54	6.05E-03
P21796	Voltage-dependent anion-selective channel protein 1 OS=Homo sapiens GN=VDAC1 PE=1 SV=2 - [VDAC1_HUMAN]	81.27	17	431	30.75	8.5	1.55	4.97E-03
Q9Y5M8	Signal recognition particle receptor subunit beta OS=Homo sapiens GN=SRPRB PE=1 SV=3 - [SRPRB_HUMAN]	44.28	11	37	29.68	9.0	1.55	1.88E-02
O43752	Syntaxin-6 OS=Homo sapiens GN=STX6 PE=1 SV=1 - [STX6_HUMAN]	9.8	1	1	29.16	4.9	1.55	1.96E-02

B4DZ53	cDNA FLJ59643, highly similar to Neutral alpha-glucosidase AB OS=Homo sapiens PE=2 SV=1 - [B4DZ53_HUMAN]	38.03	1	94	96.47	6.0	1.55	7.98E-03
O43402	ER membrane protein complex subunit 8 OS=Homo sapiens GN=EMC8 PE=1 SV=1 - [EMC8_HUMAN]	25.24	4	10	23.76	6.4	1.55	1.65E-02
Q9GZT3	SRA stem-loop-interacting RNA-binding protein, mitochondrial OS=Homo sapiens GN=SLIRP PE=1 SV=1 - [SLIRP_HUMAN]	59.63	7	18	12.34	10.2	1.55	4.41E-03
A8MXV4	Nucleoside diphosphate-linked moiety X motif 19 OS=Homo sapiens GN=NUDT19 PE=1 SV=1 - [NUD19_HUMAN]	17.87	5	11	42.21	7.6	1.55	6.40E-03
C9JBY7	28S ribosomal protein S33, mitochondrial OS=Homo sapiens GN=MRPS33 PE=1 SV=1 - [C9JBY7_HUMAN]	21.88	2	3	11.45	10.2	1.55	6.00E-03
P53701	Cytochrome c-type heme lyase OS=Homo sapiens GN=HCCS PE=1 SV=1 - [CCHL_HUMAN]	23.13	5	9	30.58	6.7	1.55	3.06E-02
Q8NCW5	NAD(P)H-hydrate epimerase OS=Homo sapiens GN=NAXE PE=1 SV=2 - [NNRE_HUMAN]	29.86	7	16	31.65	7.7	1.55	6.34E-03
C9JE12	Transmembrane and ubiquitin-like domain-containing protein 1 (Fragment) OS=Homo sapiens GN=TMUB1 PE=1 SV=1 - [C9JE12_HUMAN]	14.47	2	2	16.11	4.9	1.55	8.97E-04
P14406	Cytochrome c oxidase subunit 7A2, mitochondrial OS=Homo sapiens GN=COX7A2 PE=1 SV=1 - [CX7A2_HUMAN]	27.71	2	15	9.39	9.8	1.55	3.89E-02
Q4KWH8	1-phosphatidylinositol 4,5-bisphosphate phosphodiesterase eta-1 OS=Homo sapiens GN=PLCH1 PE=1 SV=1 - [PLCH1_HUMAN]	0.47	1	11	189.10	7.7	1.55	1.87E-02
Q9ULD0	2-oxoglutarate dehydrogenase-like, mitochondrial OS=Homo sapiens GN=OGDHL PE=1 SV=3 - [OGDHL_HUMAN]	26.83	13	58	114.41	6.7	1.55	1.25E-03

B4DQ27	cDNA FLJ59949, highly similar to Homo sapiens sulfatase modifying factor 2 (SUMF2), transcript variant 4, mRNA OS=Homo sapiens PE=2 SV=1 - [B4DQ27_HUMAN]	37.5	1	12	25.83	6.8	1.55	6.70E-03
Q5CAQ5	Tumor rejection antigen (Gp96) 1 OS=Homo sapiens GN=TRA1 PE=2 SV=1 - [Q5CAQ5_HUMAN]	58.35	44	375	92.28	4.9	1.55	4.72E-03
P00966	Argininosuccinate synthase OS=Homo sapiens GN=ASS1 PE=1 SV=2 - [ASSY_HUMAN]	19.42	7	9	46.50	8.0	1.55	9.17E-04
O15228	Dihydroxyacetone phosphate acyltransferase OS=Homo sapiens GN=GNPAT PE=1 SV=1 - [GNPAT_HUMAN]	21.32	13	23	77.14	6.6	1.55	3.51E-03
Q9H9P2	Chondrolectin OS=Homo sapiens GN=CHODL PE=2 SV=2 - [CHODL_HUMAN]	2.56	1	1	30.41	6.8	1.55	9.42E-03
Q9NVV4	Poly(A) RNA polymerase, mitochondrial OS=Homo sapiens GN=MTPAP PE=1 SV=1 - [PAPD1_HUMAN]	23.2	11	19	66.13	9.0	1.55	6.41E-03
Q502X7	Elongation of very long chain fatty acids protein OS=Homo sapiens GN=ELOVL1 PE=2 SV=1 - [Q502X7_HUMAN]	9.72	1	1	16.87	8.1	1.55	1.19E-02
P46199	Translation initiation factor IF-2, mitochondrial OS=Homo sapiens GN=MTIF2 PE=1 SV=2 - [IF2M_HUMAN]	26.27	14	33	81.27	7.2	1.55	4.69E-03
Q96EL3	39S ribosomal protein L53, mitochondrial OS=Homo sapiens GN=MRPL53 PE=1 SV=1 - [RM53_HUMAN]	43.75	4	9	12.10	8.8	1.55	5.88E-04
A8K4L6	Vang-like protein OS=Homo sapiens PE=2 SV=1 - [A8K4L6_HUMAN]	7.1	4	9	59.72	9.2	1.56	8.10E-03
B3KNK5	cDNA FLJ14795 fis, clone NT2RP4001219, highly similar to Homo sapiens DnaJ (Hsp40) homolog, subfamily C, member 10 (DNAJC10), mRNA OS=Homo sapiens PE=2 SV=1 - [B3KNK5_HUMAN]	23.88	1	23	68.92	7.8	1.56	5.28E-03

Q9NR09	Baculoviral IAP repeat-containing protein 6 OS=Homo sapiens GN=BIRC6 PE=1 SV=2 - [BIRC6_HUMAN]	0.45	1	10	529.92	6.0	1.56	7.73E-03
O15143	Actin-related protein 2/3 complex subunit 1B OS=Homo sapiens GN=ARPC1B PE=1 SV=3 - [ARC1B_HUMAN]	21.51	7	13	40.92	8.4	1.56	3.13E-02
P07237	Protein disulfide-isomerase OS=Homo sapiens GN=P4HB PE=1 SV=3 - [PDIA1_HUMAN]	70.87	26	257	57.08	4.9	1.56	7.64E-03
B3KVY9	cDNA FLJ41755 fis, clone HSYRA2009102, highly similar to Adenosine 3'-phospho 5'-phosphosulfate transporter 1 OS=Homo sapiens PE=2 SV=1 - [B3KVY9_HUMAN]	12.38	5	8	45.04	9.0	1.56	4.66E-03
Q6FIF5	Peroxisomal membrane protein 4 OS=Homo sapiens GN=PXMP4 PE=2 SV=1 - [Q6FIF5_HUMAN]	9.91	2	2	24.26	10.1	1.56	7.10E-04
Q13405	39S ribosomal protein L49, mitochondrial OS=Homo sapiens GN=MRPL49 PE=1 SV=1 - [RM49_HUMAN]	30.12	5	6	19.19	9.5	1.56	2.91E-03
H0YF65	Fibrocystin-L (Fragment) OS=Homo sapiens GN=PKHD1L1 PE=1 SV=1 - [H0YF65_HUMAN]	0.6	1	1	129.06	6.7	1.56	7.68E-03
Q9BSK2	Solute carrier family 25 member 33 OS=Homo sapiens GN=SLC25A33 PE=1 SV=1 - [S2533_HUMAN]	6.85	1	3	35.35	9.6	1.56	1.40E-02
Q9Y6N5	Sulfide:quinone oxidoreductase, mitochondrial OS=Homo sapiens GN=SQRDL PE=1 SV=1 - [SQRD_HUMAN]	62	27	95	49.93	9.1	1.56	4.04E-03
P04350	Tubulin beta-4A chain OS=Homo sapiens GN=TUBB4A PE=1 SV=2 - [TBB4A_HUMAN]	60.14	1	157	49.55	4.9	1.56	2.70E-03
Q9Y6C9	Mitochondrial carrier homolog 2 OS=Homo sapiens GN=MTCH2 PE=1 SV=1 - [MTCH2_HUMAN]	37.29	4	44	33.31	8.0	1.56	2.18E-03

A0A087X208	Agrin OS=Homo sapiens GN=AGRN PE=1 SV=1 - [A0A087X208_HUMAN]	12.95	17	18	202.16	6.4	1.56	5.59E-03
Q8WWC4	m-AAA protease-interacting protein 1, mitochondrial OS=Homo sapiens GN=C2orf47 PE=1 SV=1 - [MAIP1_HUMAN]	17.18	5	20	32.52	9.2	1.56	8.40E-03
Q14126	Desmoglein-2 OS=Homo sapiens GN=DSG2 PE=1 SV=2 - [DSG2_HUMAN]	9.84	6	7	122.22	5.2	1.56	8.67E-03
A2VDJ4	GNPTG protein (Fragment) OS=Homo sapiens GN=GNPTG PE=2 SV=1 - [A2VDJ4_HUMAN]	3.42	1	1	32.94	6.8	1.57	1.81E-02
Q9BY49	Peroxisomal trans-2-enoyl-CoA reductase OS=Homo sapiens GN=PECR PE=1 SV=2 - [PECR_HUMAN]	26.4	7	10	32.52	8.8	1.57	4.14E-04
B2R8A2	cDNA, FLJ93804, highly similar to Homo sapiens gp25L2 protein (HSGP25L2G), mRNA OS=Homo sapiens PE=2 SV=1 - [B2R8A2_HUMAN]	43.93	10	22	25.06	7.2	1.57	1.04E-02
B2R7I6	cDNA, FLJ93460, highly similar to Homo sapiens peroxisomal membrane protein 3, 35kDa (Zellweger syndrome) (PXMP3), mRNA OS=Homo sapiens PE=2 SV=1 - [B2R7I6_HUMAN]	6.56	2	4	34.87	8.7	1.57	7.15E-04
Q4U2R6	39S ribosomal protein L51, mitochondrial OS=Homo sapiens GN=MRPL51 PE=1 SV=1 - [RM51_HUMAN]	5.47	1	1	15.09	11.3	1.57	8.25E-03
J3QRN3	Zinc transporter ZIP11 OS=Homo sapiens GN=SLC39A11 PE=1 SV=1 - [J3QRN3_HUMAN]	23.46	1	2	8.54	5.3	1.57	1.03E-02
Q13907	Isopentenyl-diphosphate Delta-isomerase 1 OS=Homo sapiens GN=IDI1 PE=1 SV=2 - [IDI1_HUMAN]	8.37	2	6	26.30	6.3	1.57	5.11E-03
O75792	Ribonuclease H2 subunit A OS=Homo sapiens GN=RNASEH2A PE=1 SV=2 - [RNH2A_HUMAN]	2.68	1	7	33.37	5.2	1.57	1.08E-03

O14672	Disintegrin and metalloproteinase domain-containing protein 10 OS=Homo sapiens GN=ADAM10 PE=1 SV=1 - [ADA10_HUMAN]	10.83	7	9	84.09	7.8	1.57	3.42E-04
B3KWR9	Toll-like receptor OS=Homo sapiens PE=2 SV=1 - [B3KWR9_HUMAN]	2.93	2	3	89.77	6.6	1.57	8.61E-03
H0Y8F6	3-hydroxy-3-methylglutaryl-coenzyme A reductase (Fragment) OS=Homo sapiens GN=HMGCR PE=1 SV=1 - [H0Y8F6_HUMAN]	6.96	1	1	12.30	8.8	1.57	3.76E-03
U3KQU8	Oxidoreductase NAD-binding domain-containing protein 1 OS=Homo sapiens GN=OXNAD1 PE=1 SV=1 - [U3KQU8_HUMAN]	8.04	3	3	34.70	8.5	1.57	3.74E-03
A0A024R2H7	tRNA nucleotidyl transferase, CCA-adding, 1, isoform CRA_b OS=Homo sapiens GN=TRNT1 PE=3 SV=1 - [A0A024R2H7_HUMAN]	28.8	9	11	50.11	8.1	1.58	2.66E-03
I3L0Y5	ATP synthase F(0) complex subunit C1, mitochondrial (Fragment) OS=Homo sapiens GN=ATP5G1 PE=3 SV=1 - [I3L0Y5_HUMAN]	7.14	1	6	10.03	10.0	1.58	9.78E-03
O43464	Serine protease HTRA2, mitochondrial OS=Homo sapiens GN=HTRA2 PE=1 SV=2 - [HTRA2_HUMAN]	13.1	4	7	48.81	10.1	1.58	8.22E-03
Q7Z3K9	Putative uncharacterized protein DKFZp781L0540 (Fragment) OS=Homo sapiens GN=DKFZp781L0540 PE=2 SV=1 - [Q7Z3K9_HUMAN]	2.63	2	4	89.97	5.6	1.58	6.85E-04
S6AP35	MHC class II antigen (Fragment) OS=Homo sapiens GN=HLA-DQB1 PE=3 SV=1 - [S6AP35_HUMAN]	45.08	1	21	41.16	6.6	1.58	4.23E-02
Q8WXX5	DnaJ homolog subfamily C member 9 OS=Homo sapiens GN=DNAJC9 PE=1 SV=1 - [DNJC9_HUMAN]	4.23	1	1	29.89	5.7	1.58	1.30E-02
Q9H4A6	Golgi phosphoprotein 3 OS=Homo sapiens GN=GOLPH3 PE=1 SV=1 - [GOLP3_HUMAN]	9.73	2	4	33.79	6.4	1.58	2.79E-04

Q6NVY1	3-hydroxyisobutyryl-CoA hydrolase, mitochondrial OS=Homo sapiens GN=HIBCH PE=1 SV=2 - [HIBCH_HUMAN]	34.46	14	29	43.45	8.2	1.58	2.50E-03
Q9H617	Transmembrane protein 164 OS=Homo sapiens GN=RP13-360B22.2 PE=1 SV=1 - [Q9H617_HUMAN]	6.98	1	1	29.21	7.5	1.58	4.18E-02
O00519	Fatty-acid amide hydrolase 1 OS=Homo sapiens GN=FAAH PE=1 SV=2 - [FAAH1_HUMAN]	11.05	5	6	63.03	7.7	1.58	2.60E-03
Q9UH62	Armadillo repeat-containing X-linked protein 3 OS=Homo sapiens GN=ARMCX3 PE=1 SV=1 - [ARMX3_HUMAN]	18.47	6	7	42.47	8.4	1.58	1.13E-02
B2RA30	cDNA, FLJ94673, highly similar to Homo sapiens potassium channel, subfamily K, member 1 (KCNK1), mRNA OS=Homo sapiens PE=2 SV=1 - [B2RA30_HUMAN]	5.65	1	1	38.14	6.4	1.58	1.34E-02
Q49A71	5-demethoxyubiquinone hydroxylase, mitochondrial OS=Homo sapiens GN=COQ7 PE=2 SV=1 - [Q49A71_HUMAN]	49.48	8	14	21.89	6.9	1.59	1.26E-02
B3KSE0	cDNA FLJ36069 fis, clone TESTI2019406, highly similar to HEME OXYGENASE 2 (EC 1.14.99.3) OS=Homo sapiens PE=2 SV=1 - [B3KSE0_HUMAN]	15.82	5	14	35.94	5.5	1.59	2.47E-03
B2R6T2	cDNA, FLJ93100, highly similar to Homo sapiens calpain 6 (CAPN6), mRNA OS=Homo sapiens PE=2 SV=1 - [B2R6T2_HUMAN]	8.42	4	5	74.54	7.0	1.59	7.70E-03
E9PF19	Transducin beta-like protein 2 OS=Homo sapiens GN=TBL2 PE=1 SV=1 - [E9PF19_HUMAN]	48.91	16	42	45.91	9.5	1.59	2.19E-02
A8K4K9	cDNA FLJ76169 OS=Homo sapiens PE=2 SV=1 - [A8K4K9_HUMAN]	24.24	6	11	34.84	6.8	1.59	1.71E-02
A0A087WVA1	Selenoprotein T OS=Homo sapiens GN=SELT PE=1 SV=1 - [A0A087WVA1_HUMAN]	10.26	2	2	22.33	8.8	1.59	1.39E-02

O95831	Apoptosis-inducing factor 1, mitochondrial OS=Homo sapiens GN=AIFM1 PE=1 SV=1 - [AIFM1_HUMAN]	46.66	24	158	66.86	9.0	1.59	2.76E-03
B4DJV2	Citrate synthase OS=Homo sapiens GN=CS PE=1 SV=1 - [B4DJV2_HUMAN]	26.93	13	73	50.40	7.9	1.59	4.65E-03
I3L0U2	Testisin (Fragment) OS=Homo sapiens GN=PRSS21 PE=1 SV=1 - [I3L0U2_HUMAN]	7.34	1	1	24.80	8.3	1.59	2.30E-03
Q5JT02	Protein O-mannosyl-transferase 1 (Fragment) OS=Homo sapiens GN=POMT1 PE=1 SV=1 - [Q5JT02_HUMAN]	5.49	1	2	39.41	9.0	1.59	1.13E-02
P07919	Cytochrome b-c1 complex subunit 6, mitochondrial OS=Homo sapiens GN=UQCRH PE=1 SV=2 - [QCR6_HUMAN]	51.65	5	18	10.73	4.4	1.59	1.63E-02
B4DKB9	cDNA FLJ54085, highly similar to Homo sapiens calcium binding and coiled-coil domain 1 (CALCOCO1), mRNA OS=Homo sapiens PE=2 SV=1 - [B4DKB9_HUMAN]	5.96	1	1	25.08	4.9	1.59	4.13E-03
A8K4Q9	cDNA FLJ77931 OS=Homo sapiens PE=2 SV=1 - [A8K4Q9_HUMAN]	9.92	7	13	102.45	7.4	1.59	3.12E-03
P61019	Ras-related protein Rab-2A OS=Homo sapiens GN=RAB2A PE=1 SV=1 - [RAB2A_HUMAN]	57.08	5	34	23.53	6.5	1.59	1.37E-02
P43490	Nicotinamide phosphoribosyltransferase OS=Homo sapiens GN=NAMPT PE=1 SV=1 - [NAMPT_HUMAN]	34.01	13	26	55.49	7.2	1.59	1.06E-02
Q99797	Mitochondrial intermediate peptidase OS=Homo sapiens GN=MIPEP PE=1 SV=2 - [MIPEP_HUMAN]	20.76	12	19	80.59	7.0	1.59	4.68E-03
Q96C36	Pyrroline-5-carboxylate reductase 2 OS=Homo sapiens GN=PYCR2 PE=1 SV=1 - [P5CR2_HUMAN]	45	11	34	33.62	7.8	1.59	9.38E-03

A8K337	cDNA FLJ77862, highly similar to Homo sapiens catechol-O-methyltransferase domain containing 1 (COMTD1), mRNA OS=Homo sapiens PE=2 SV=1 - [A8K337_HUMAN]	19.47	5	6	28.82	8.4	1.59	2.79E-02
O43708	Maleylacetoacetate isomerase OS=Homo sapiens GN=GSTZ1 PE=1 SV=3 - [MAAI_HUMAN]	26.85	1	7	24.20	8.5	1.59	3.61E-03
A8K6K4	cDNA FLJ77565, highly similar to Homo sapiens interleukin 1 receptor accessory protein (IL1RAP), transcript variant 1, mRNA OS=Homo sapiens PE=2 SV=1 - [A8K6K4_HUMAN]	6.49	4	6	65.39	8.2	1.60	3.03E-04
P50213	Isocitrate dehydrogenase [NAD] subunit alpha, mitochondrial OS=Homo sapiens GN=IDH3A PE=1 SV=1 - [IDH3A_HUMAN]	47.81	18	53	39.57	6.9	1.60	2.27E-02
B4DP77	cDNA FLJ57413, highly similar to Mitochondrial 28S ribosomal protein S10 OS=Homo sapiens PE=2 SV=1 - [B4DP77_HUMAN]	35	4	11	18.72	6.4	1.60	1.12E-02
Q13753	Laminin subunit gamma-2 OS=Homo sapiens GN=LAMC2 PE=1 SV=2 - [LAMC2_HUMAN]	4.02	4	4	130.89	6.2	1.60	4.91E-03
Q7Z406	Myosin-14 OS=Homo sapiens GN=MYH14 PE=1 SV=2 - [MYH14_HUMAN]	40.35	34	170	227.73	5.6	1.60	4.32E-04
O95716	Ras-related protein Rab-3D OS=Homo sapiens GN=RAB3D PE=1 SV=1 - [RAB3D_HUMAN]	30.59	4	16	24.25	4.9	1.60	3.87E-03
I3RW89	MHC class Ib antigen (Fragment) OS=Homo sapiens GN=HLA-E PE=3 SV=1 - [I3RW89_HUMAN]	25.42	3	11	33.78	5.4	1.60	3.08E-02
B3KM26	cDNA FLJ10050 fis, clone HEMBA1001257, highly similar to Alpha-methylacyl-CoA racemase (EC 5.1.99.4) OS=Homo sapiens PE=2 SV=1 - [B3KM26_HUMAN]	1.83	1	1	42.36	6.7	1.60	3.30E-03
Q96AB3	Isochorismatase domain-containing protein 2 OS=Homo sapiens GN=ISOC2 PE=1 SV=1 - [ISOC2_HUMAN]	59.02	7	23	22.32	7.8	1.60	9.79E-03

C9JFL0	Protein FAM185A (Fragment) OS=Homo sapiens GN=FAM185A PE=1 SV=8 - [C9JFL0_HUMAN]	17.53	3	3	21.12	7.2	1.60	3.81E-03
X5CMH5	Antigen peptide transporter 2 OS=Homo sapiens GN=TAP2 PE=1 SV=1 - [X5CMH5_HUMAN]	28.31	17	36	77.65	7.9	1.60	1.20E-03
D3DUJ0	AFG3 ATPase family gene 3-like 2 (Yeast), isoform CRA_a (Fragment) OS=Homo sapiens GN=AFG3L2 PE=3 SV=1 - [D3DUJ0_HUMAN]	46.64	32	122	84.39	8.4	1.60	1.88E-04
Q9HB66	Alternative protein MKKS OS=Homo sapiens GN=MKKS PE=1 SV=1 - [Q9HB66_HUMAN]	41.27	2	5	7.26	9.6	1.60	1.47E-02
K7EJJ1	AP-1 complex subunit mu-2 (Fragment) OS=Homo sapiens GN=AP1M2 PE=1 SV=1 - [K7EJJ1_HUMAN]	6.94	1	1	27.87	5.6	1.60	3.30E-03
Q8TCT9	Minor histocompatibility antigen H13 OS=Homo sapiens GN=HM13 PE=1 SV=1 - [HM13_HUMAN]	23.08	10	31	41.46	6.4	1.60	3.25E-03
P01833	Polymeric immunoglobulin receptor OS=Homo sapiens GN=PIGR PE=1 SV=4 - [PIGR_HUMAN]	14.14	9	18	83.23	5.7	1.61	1.96E-02
A8K454	cDNA FLJ78187, highly similar to Homo sapiens calmegin, mRNA OS=Homo sapiens PE=2 SV=1 - [A8K454_HUMAN]	11.48	5	9	69.99	4.7	1.61	2.43E-04
A0A1B0GTB0	Renin receptor (Fragment) OS=Homo sapiens GN=ATP6AP2 PE=1 SV=1 - [A0A1B0GTB0_HUMAN]	20.41	1	8	33.00	6.1	1.61	3.61E-03
B4DZ87	cDNA FLJ57240, highly similar to Mitochondrial proteins import receptor OS=Homo sapiens PE=2 SV=1 - [B4DZ87_HUMAN]	47.7	24	70	56.89	5.9	1.61	2.84E-03
P21757	Macrophage scavenger receptor types I and II OS=Homo sapiens GN=MSR1 PE=1 SV=1 - [MSRE_HUMAN]	1.55	1	1	49.73	5.9	1.61	8.10E-04

Q92968	Peroxisomal membrane protein PEX13 OS=Homo sapiens GN=PEX13 PE=1 SV=2 - [PEX13_HUMAN]	2.48	1	2	44.10	8.0	1.61	3.58E-03
Q3MIX3	Uncharacterized aarF domain-containing protein kinase 5 OS=Homo sapiens GN=ADCK5 PE=1 SV=2 - [ADCK5_HUMAN]	12.24	6	10	65.85	9.0	1.61	2.49E-04
X6RIW1	39S ribosomal protein L55, mitochondrial (Fragment) OS=Homo sapiens GN=MRPL55 PE=1 SV=1 - [X6RIW1_HUMAN]	10.53	1	2	8.61	11.9	1.61	9.50E-03
P07954	Fumarate hydratase, mitochondrial OS=Homo sapiens GN=FH PE=1 SV=3 - [FUMH_HUMAN]	52.16	20	137	54.60	8.8	1.61	8.84E-03
P08311	Cathepsin G OS=Homo sapiens GN=CTSG PE=1 SV=2 - [CATG_HUMAN]	35.69	7	13	28.82	11.2	1.62	2.97E-02
Q8N490	Probable hydrolase PNKD OS=Homo sapiens GN=PNKD PE=1 SV=2 - [PNKD_HUMAN]	9.61	2	5	42.85	9.1	1.62	1.13E-02
Q9NPJ3	Acyl-coenzyme A thioesterase 13 OS=Homo sapiens GN=ACOT13 PE=1 SV=1 - [ACO13_HUMAN]	25	3	8	14.95	9.1	1.62	4.47E-03
P15144	Aminopeptidase N OS=Homo sapiens GN=ANPEP PE=1 SV=4 - [AMPN_HUMAN]	13.75	10	14	109.47	5.5	1.62	1.75E-02
P0DMU7	Cancer/testis antigen family 45 member A6 OS=Homo sapiens GN=CT45A6 PE=2 SV=1 - [CT456_HUMAN]	3.7	1	1	21.22	9.7	1.62	1.53E-02
B2MUD5	Neutrophil elastase (Fragment) OS=Homo sapiens GN=ELA2 PE=4 SV=1 - [B2MUD5_HUMAN]	13.02	3	4	20.65	8.9	1.62	9.32E-04
P43897	Elongation factor Ts, mitochondrial OS=Homo sapiens GN=TSFM PE=1 SV=2 - [EFTS_HUMAN]	43.69	12	32	35.37	8.4	1.62	2.12E-03

B3KQF7	cDNA FLJ90383 fis, clone NT2RP2005163 OS=Homo sapiens PE=2 SV=1 - [B3KQF7_HUMAN]	10.49	3	5	53.52	6.4	1.62	2.30E-03
Q08426	Peroxisomal bifunctional enzyme OS=Homo sapiens GN=EHHADH PE=1 SV=3 - [ECHP_HUMAN]	33.47	22	34	79.44	9.1	1.62	2.25E-03
Q13217	DnaJ homolog subfamily C member 3 OS=Homo sapiens GN=DNAJC3 PE=1 SV=1 - [DNJC3_HUMAN]	26.39	11	18	57.54	6.2	1.62	2.70E-02
Q9Y6H1	Coiled-coil-helix-coiled-coil-helix domain-containing protein 2 OS=Homo sapiens GN=CHCHD2 PE=1 SV=1 - [CHCH2_HUMAN]	15.23	2	9	15.50	9.2	1.62	2.42E-02
A0A140VJK2	Glycerol-3-phosphate dehydrogenase OS=Homo sapiens PE=2 SV=1 - [A0A140VJK2_HUMAN]	63.82	44	162	80.78	7.5	1.62	4.27E-03
A0A087WWV7	Uncharacterized protein KIAA1755 (Fragment) OS=Homo sapiens GN=KIAA1755 PE=1 SV=1 - [A0A087WWV7_HUMAN]	18.42	1	1	3.87	10.7	1.62	1.61E-02
Q8NBT6	cDNA FLJ90758 fis, clone SKNMC1000082, highly similar to Mitochondrial deoxynucleotide carrier OS=Homo sapiens PE=2 SV=1 - [Q8NBT6_HUMAN]	18.75	7	7	35.45	9.5	1.62	4.01E-03
A6NNL5	Uncharacterized protein C15orf61 OS=Homo sapiens GN=C15orf61 PE=2 SV=2 - [CO061_HUMAN]	3.82	1	1	18.08	9.9	1.62	5.95E-03
Q6UXH1	Cysteine-rich with EGF-like domain protein 2 OS=Homo sapiens GN=CRELD2 PE=1 SV=1 - [CREL2_HUMAN]	16.71	5	8	38.17	4.6	1.63	3.62E-03
F8VUJ3	Polypeptide N-acetylgalactosaminyltransferase OS=Homo sapiens GN=POC1B-GALNT4 PE=3 SV=1 - [F8VUJ3_HUMAN]	20.52	11	17	66.20	7.5	1.63	1.64E-02
P82933	28S ribosomal protein S9, mitochondrial OS=Homo sapiens GN=MRPS9 PE=1 SV=2 - [RT09_HUMAN]	39.14	13	29	45.81	9.5	1.63	1.98E-02

H0Y2W2	ATPase family AAA domain-containing protein 3A (Fragment) OS=Homo sapiens GN=ATAD3A PE=1 SV=1 - [H0Y2W2_HUMAN]	43.01	2	90	64.31	9.4	1.63	9.40E-03
P27449	V-type proton ATPase 16 kDa proteolipid subunit OS=Homo sapiens GN=ATP6V0C PE=1 SV=1 - [VATL_HUMAN]	11.61	1	18	15.73	8.4	1.63	4.24E-04
Q96DB5	Regulator of microtubule dynamics protein 1 OS=Homo sapiens GN=RMDN1 PE=1 SV=1 - [RMD1_HUMAN]	27.39	7	11	35.79	8.5	1.63	3.53E-02
C9JAX1	Frataxin, mitochondrial OS=Homo sapiens GN=FXN PE=1 SV=1 - [C9JAX1_HUMAN]	38.52	4	10	14.90	5.1	1.63	1.45E-02
C9J3L8	Translocon-associated protein subunit alpha OS=Homo sapiens GN=SSR1 PE=1 SV=1 - [C9J3L8_HUMAN]	12.83	3	45	29.55	4.3	1.63	3.61E-03
Q9NUN5	Probable lysosomal cobalamin transporter OS=Homo sapiens GN=LMBRD1 PE=1 SV=1 - [LMBD1_HUMAN]	3.33	1	1	61.35	7.8	1.63	2.12E-02
O95721	Synaptosomal-associated protein 29 OS=Homo sapiens GN=SNAP29 PE=1 SV=1 - [SNP29_HUMAN]	8.14	2	2	28.95	5.8	1.63	1.96E-02
Q8N118	Cytochrome P450 4X1 OS=Homo sapiens GN=CYP4X1 PE=2 SV=1 - [CP4X1_HUMAN]	10.02	5	5	58.84	8.5	1.63	1.25E-02
B2R6Q2	Alkaline phosphatase OS=Homo sapiens PE=2 SV=1 - [B2R6Q2_HUMAN]	7.25	4	5	57.24	6.6	1.64	2.66E-03
Q13162	Peroxiredoxin-4 OS=Homo sapiens GN=PRDX4 PE=1 SV=1 - [PRDX4_HUMAN]	62.36	10	55	30.52	6.3	1.64	4.37E-02
P12318	Low affinity immunoglobulin gamma Fc region receptor II-a OS=Homo sapiens GN=FCGR2A PE=1 SV=4 - [FCG2A_HUMAN]	3.15	1	1	34.98	6.7	1.64	5.95E-03

O60220	Mitochondrial import inner membrane translocase subunit Tim8 A OS=Homo sapiens GN=TIMM8A PE=1 SV=1 - [TIM8A_HUMAN]	38.14	3	7	10.99	5.2	1.64	1.82E-02
P42126	Enoyl-CoA delta isomerase 1, mitochondrial OS=Homo sapiens GN=ECI1 PE=1 SV=1 - [ECI1_HUMAN]	34.44	8	50	32.80	8.5	1.64	1.99E-03
Q86SF2	N-acetylgalactosaminyltransferase 7 OS=Homo sapiens GN=GALNT7 PE=1 SV=1 - [GALT7_HUMAN]	17.05	10	15	75.34	7.1	1.64	4.07E-03
Q53HR4	Chromosome 15 open reading frame 22 variant (Fragment) OS=Homo sapiens PE=2 SV=1 - [Q53HR4_HUMAN]	22.12	3	6	24.75	5.8	1.64	1.10E-02
K7EMM8	Putative oxidoreductase GLYR1 (Fragment) OS=Homo sapiens GN=GLYR1 PE=1 SV=2 - [K7EMM8_HUMAN]	3.24	1	2	57.29	9.2	1.64	2.35E-03
Q9BV61	TRAP1 protein (Fragment) OS=Homo sapiens GN=TRAP1 PE=2 SV=2 - [Q9BV61_HUMAN]	42.78	4	135	79.31	7.8	1.64	8.92E-03
P11117	Lysosomal acid phosphatase OS=Homo sapiens GN=ACP2 PE=1 SV=3 - [PPAL_HUMAN]	14.66	6	12	48.31	6.7	1.64	6.07E-03
B3KPI5	cDNA FLJ31840 fis, clone NT2RP7000109, highly similar to Tudor and KH domain-containing protein OS=Homo sapiens PE=2 SV=1 - [B3KPI5_HUMAN]	1.08	1	1	61.45	5.0	1.64	1.03E-03
Q5JRX3	Presequence protease, mitochondrial OS=Homo sapiens GN=PITRM1 PE=1 SV=3 - [PREP_HUMAN]	46.58	39	112	117.34	6.9	1.64	1.19E-02
P53794	Sodium/myo-inositol cotransporter OS=Homo sapiens GN=SLC5A3 PE=1 SV=2 - [SC5A3_HUMAN]	5.71	3	5	79.64	7.3	1.65	1.98E-03
Q9BW75	THOP1 protein (Fragment) OS=Homo sapiens GN=THOP1 PE=2 SV=2 - [Q9BW75_HUMAN]	3.72	2	2	67.84	6.5	1.65	8.50E-04

Q5R3K3	Protein FAM26F OS=Homo sapiens GN=FAM26F PE=2 SV=1 - [FA26F_HUMAN]	2.86	1	1	34.44	8.6	1.65	2.93E-04
B4DWH7	cDNA FLJ57632, highly similar to Vacuolar ATP synthase subunit B, kidney isoform (EC 3.6.3.14) OS=Homo sapiens PE=2 SV=1 - [B4DWH7_HUMAN]	16.39	3	7	54.26	5.7	1.65	6.18E-03
Q9H857	5'-nucleotidase domain-containing protein 2 OS=Homo sapiens GN=NT5DC2 PE=1 SV=1 - [NT5D2_HUMAN]	31.54	14	27	60.68	6.8	1.65	1.37E-03
Q9Y3A6	Transmembrane emp24 domain-containing protein 5 OS=Homo sapiens GN=TMED5 PE=1 SV=1 - [TMED5_HUMAN]	17.03	4	14	25.99	4.8	1.65	1.30E-02
Q15293	Reticulocalbin-1 OS=Homo sapiens GN=RCN1 PE=1 SV=1 - [RCN1_HUMAN]	31.72	9	30	38.87	5.0	1.65	7.56E-04
Q96HE7	ERO1-like protein alpha OS=Homo sapiens GN=ERO1A PE=1 SV=2 - [ERO1A_HUMAN]	52.14	18	47	54.36	5.7	1.65	1.48E-02
P61956	Small ubiquitin-related modifier 2 OS=Homo sapiens GN=SUMO2 PE=1 SV=3 - [SUMO2_HUMAN]	12.63	1	1	10.86	5.5	1.65	1.25E-02
G8JLB3	tRNA pseudouridine synthase (Fragment) OS=Homo sapiens GN=PUS1 PE=1 SV=1 - [G8JLB3_HUMAN]	37.24	11	17	42.89	8.5	1.65	6.41E-04
Q99467	CD180 antigen OS=Homo sapiens GN=CD180 PE=1 SV=2 - [CD180_HUMAN]	3.78	1	1	74.13	6.4	1.66	3.38E-02
A8K610	cDNA FLJ76619 OS=Homo sapiens PE=2 SV=1 - [A8K610_HUMAN]	9.62	2	2	23.08	9.0	1.66	3.74E-02
Q96SL4	Glutathione peroxidase 7 OS=Homo sapiens GN=GPX7 PE=1 SV=1 - [GPX7_HUMAN]	33.16	5	9	20.98	8.3	1.66	2.77E-04

Q6IN84	rRNA methyltransferase 1, mitochondrial OS=Homo sapiens GN=MRM1 PE=1 SV=1 - [MRM1_HUMAN]	3.12	1	1	38.61	7.9	1.67	6.11E-05
P50583	Bis(5'-nucleosyl)-tetraphosphatase [asymmetrical] OS=Homo sapiens GN=NUDT2 PE=1 SV=3 - [AP4A_HUMAN]	9.52	1	1	16.82	5.4	1.67	2.05E-03
A0A142L067	MHC class II antigen (Fragment) OS=Homo sapiens GN=HLA-DQB1 PE=3 SV=1 - [A0A142L067_HUMAN]	24.11	3	5	25.87	7.2	1.67	1.55E-02
Q53F67	Kringle-containing transmembrane protein 2 isoform b variant (Fragment) OS=Homo sapiens PE=2 SV=1 - [Q53F67_HUMAN]	5.71	1	1	44.42	6.2	1.67	2.04E-03
H7C5A7	Acyl-coenzyme A thioesterase 8 (Fragment) OS=Homo sapiens GN=ACOT8 PE=1 SV=1 - [H7C5A7_HUMAN]	18.66	2	7	23.43	9.6	1.67	4.07E-03
A0A0A0MRM8	Unconventional myosin-VI OS=Homo sapiens GN=MYO6 PE=1 SV=1 - [A0A0A0MRM8_HUMAN]	22.59	22	36	144.92	8.6	1.67	4.34E-03
A8DU55	Connexin 26 (Fragment) OS=Homo sapiens GN=GJB2 PE=4 SV=1 - [A8DU55_HUMAN]	16.8	2	2	14.44	9.1	1.67	8.96E-03
I3L3P5	Protein disulfide-isomerase (Fragment) OS=Homo sapiens GN=P4HB PE=1 SV=1 - [I3L3P5_HUMAN]	57.69	1	34	16.87	4.8	1.67	1.36E-03
P11215	Integrin alpha-M OS=Homo sapiens GN=ITGAM PE=1 SV=2 - [ITAM_HUMAN]	9.9	10	14	127.10	7.2	1.67	2.05E-03
P29762	Cellular retinoic acid-binding protein 1 OS=Homo sapiens GN=CRABP1 PE=1 SV=2 - [RABP1_HUMAN]	14.6	2	3	15.56	5.4	1.67	1.14E-02
P54792	Putative segment polarity protein dishevelled homolog DVL1P1 OS=Homo sapiens GN=DVL1P1 PE=5 SV=1 - [DVLP1_HUMAN]	1.19	1	1	73.21	6.5	1.67	4.88E-04

Q6PIU2	Neutral cholesterol ester hydrolase 1 OS=Homo sapiens GN=NCEH1 PE=1 SV=3 - [NCEH1_HUMAN]	12.99	5	11	45.78	7.2	1.67	3.07E-03
B2R761	cDNA, FLJ93299, highly similar to Homo sapiens sterol carrier protein 2 (SCP2), mRNA OS=Homo sapiens PE=2 SV=1 - [B2R761_HUMAN]	44.42	20	75	58.98	7.0	1.68	8.63E-04
O75844	CAAX prenyl protease 1 homolog OS=Homo sapiens GN=ZMPSTE24 PE=1 SV=2 - [FACE1_HUMAN]	20.63	9	27	54.78	7.5	1.68	2.16E-03
B1Q2B0	URCC5 OS=Homo sapiens GN=URCC5 PE=2 SV=1 - [B1Q2B0_HUMAN]	7.68	7	11	101.24	8.4	1.68	5.31E-03
B2R6A3	Na(+)/H(+) exchange regulatory cofactor NHE-RF OS=Homo sapiens PE=2 SV=1 - [B2R6A3_HUMAN]	6.7	2	2	38.87	5.8	1.68	2.71E-04
F6VGH2	Kinesin-like protein KIF6 OS=Homo sapiens GN=KIF6 PE=4 SV=2 - [F6VGH2_HUMAN]	4.1	1	1	33.12	7.2	1.68	5.35E-03
Q8TDB6	E3 ubiquitin-protein ligase DTX3L OS=Homo sapiens GN=DTX3L PE=1 SV=1 - [DTX3L_HUMAN]	9.86	6	7	83.50	8.1	1.68	8.05E-03
B4DVE1	cDNA FLJ53478, highly similar to Galectin-3-binding protein OS=Homo sapiens PE=2 SV=1 - [B4DVE1_HUMAN]	28.45	14	42	64.06	5.5	1.68	5.58E-03
Q14657	EKC/KEOPS complex subunit LAGE3 OS=Homo sapiens GN=LAGE3 PE=1 SV=2 - [LAGE3_HUMAN]	11.19	1	1	14.79	8.6	1.68	7.15E-03
C5HTZ1	Amiloride-sensitive sodium channel subunit alpha OS=Homo sapiens GN=SCNN1A PE=1 SV=1 - [C5HTZ1_HUMAN]	4.49	1	1	28.38	6.7	1.68	5.20E-03
Q14696	LDLR chaperone MESD OS=Homo sapiens GN=MESDC2 PE=1 SV=2 - [MESD_HUMAN]	34.62	8	13	26.06	7.8	1.68	8.45E-03

A0A097Q0T5	Cytochrome c oxidase subunit 2 OS=Homo sapiens GN=COX2 PE=3 SV=1 - [A0A097Q0T5_HUMAN]	26.43	1	64	25.55	4.8	1.68	4.38E-02
A0A0S2Z3D2	BCL2-like 1 isoform 3 OS=Homo sapiens GN=BCL2L1 PE=2 SV=1 - [A0A0S2Z3D2_HUMAN]	5.96	1	2	16.69	5.0	1.68	8.14E-03
Q9NPA0	ER membrane protein complex subunit 7 OS=Homo sapiens GN=EMC7 PE=1 SV=1 - [EMC7_HUMAN]	29.75	5	9	26.45	9.2	1.69	1.50E-02
Q9NTX5	Ethylmalonyl-CoA decarboxylase OS=Homo sapiens GN=ECHDC1 PE=1 SV=2 - [ECHD1_HUMAN]	9.45	3	10	33.68	8.2	1.69	3.67E-03
B4DGN8	cDNA FLJ53377, highly similar to Procollagen-lysine, 2-oxoglutarate 5-dioxygenase 1 (EC 1.14.11.4) OS=Homo sapiens PE=2 SV=1 - [B4DGN8_HUMAN]	27.83	15	29	79.35	7.0	1.69	1.56E-02
O75396	Vesicle-trafficking protein SEC22b OS=Homo sapiens GN=SEC22B PE=1 SV=4 - [SC22B_HUMAN]	29.3	6	19	24.58	6.9	1.69	1.32E-03
P08727	Keratin, type I cytoskeletal 19 OS=Homo sapiens GN=KRT19 PE=1 SV=4 - [K1C19_HUMAN]	27.75	5	13	44.08	5.1	1.69	3.01E-03
P18065	Insulin-like growth factor-binding protein 2 OS=Homo sapiens GN=IGFBP2 PE=1 SV=2 - [IBP2_HUMAN]	12.31	3	3	34.79	7.5	1.69	1.32E-03
B2R659	cDNA, FLJ92803, highly similar to Homo sapiens hydroxysteroid (17-beta) dehydrogenase 4 (HSD17B4), mRNA OS=Homo sapiens PE=2 SV=1 - [B2R659_HUMAN]	48.1	3	80	79.56	8.9	1.69	6.47E-04
P20591	Interferon-induced GTP-binding protein Mx1 OS=Homo sapiens GN=MX1 PE=1 SV=4 - [MX1_HUMAN]	33.99	15	35	75.47	5.8	1.69	2.87E-03
Q7L8L6	FAST kinase domain-containing protein 5, mitochondrial OS=Homo sapiens GN=FASTKD5 PE=1 SV=1 - [FAKD5_HUMAN]	20.42	14	17	86.52	8.1	1.70	6.11E-03

J7RE54	HLA-A protein (Fragment) OS=Homo sapiens GN=HLA-A PE=3 SV=1 - [J7RE54_HUMAN]	51.28	1	19	31.60	5.7	1.70	5.24E-03
P02792	Ferritin light chain OS=Homo sapiens GN=FTL PE=1 SV=2 - [FRIL_HUMAN]	36.57	6	12	20.01	5.8	1.70	8.98E-03
P78536	Disintegrin and metalloproteinase domain-containing protein 17 OS=Homo sapiens GN=ADAM17 PE=1 SV=1 - [ADA17_HUMAN]	5.95	4	4	92.96	5.8	1.70	7.42E-03
B7Z4Y5	cDNA FLJ51553, highly similar to HLA class II histocompatibility antigen, DMalpha chain OS=Homo sapiens PE=2 SV=1 - [B7Z4Y5_HUMAN]	27.64	2	3	14.11	4.4	1.70	8.19E-03
C9J8T4	E3 ubiquitin-protein ligase RNF13 (Fragment) OS=Homo sapiens GN=RNF13 PE=1 SV=1 - [C9J8T4_HUMAN]	19.02	2	2	17.92	4.8	1.70	1.66E-03
P42704	Leucine-rich PPR motif-containing protein, mitochondrial OS=Homo sapiens GN=LRPPRC PE=1 SV=3 - [LPPRC_HUMAN]	62.41	80	306	157.81	6.1	1.70	4.79E-03
B4DYB6	Receptor expression-enhancing protein OS=Homo sapiens PE=2 SV=1 - [B4DYB6_HUMAN]	4.44	1	1	25.17	9.5	1.71	1.14E-02
Q96I99	Succinate--CoA ligase [GDP-forming] subunit beta, mitochondrial OS=Homo sapiens GN=SUCLG2 PE=1 SV=2 - [SUCB2_HUMAN]	44.91	19	115	46.48	6.4	1.71	8.17E-04
Q75MT9	Malate dehydrogenase (Fragment) OS=Homo sapiens GN=MDH2 PE=3 SV=1 - [Q75MT9_HUMAN]	74.37	21	262	33.21	8.3	1.71	5.51E-03
F2Z3N7	Transmembrane protein 106B OS=Homo sapiens GN=TMEM106B PE=1 SV=1 - [F2Z3N7_HUMAN]	12.5	1	1	11.64	6.3	1.71	3.61E-03
B4DJQ5	cDNA FLJ59211, highly similar to Glucosidase 2 subunit beta OS=Homo sapiens PE=2 SV=1 - [B4DJQ5_HUMAN]	35.89	16	88	60.10	4.4	1.71	2.85E-03

W8QEY1	Lactoferrin OS=Homo sapiens PE=2 SV=1 - [W8QEY1_HUMAN]	44.59	25	38	78.33	8.2	1.71	1.78E-04
P30101	Protein disulfide-isomerase A3 OS=Homo sapiens GN=PDIA3 PE=1 SV=4 - [PDIA3_HUMAN]	70.89	27	324	56.75	6.4	1.71	4.04E-03
A0A024RB99	Serine hydroxymethyltransferase OS=Homo sapiens GN=SHMT2 PE=3 SV=1 - [A0A024RB99_HUMAN]	55.07	23	78	53.42	8.1	1.71	9.29E-03
D6RCD9	Endosomal/lysomomal potassium channel TMEM175 OS=Homo sapiens GN=TMEM175 PE=1 SV=1 - [D6RCD9_HUMAN]	18.06	1	1	7.90	4.8	1.71	1.40E-03
A0A0A0QN99	Cytochrome b OS=Homo sapiens GN=CYTB PE=3 SV=1 - [A0A0A0QN99_HUMAN]	4.21	1	4	42.71	8.0	1.71	7.60E-03
A7LNE7	Cyclin-dependent kinase inhibitor 2A (Fragment) OS=Homo sapiens GN=CDKN2A PE=4 SV=1 - [A7LNE7_HUMAN]	11.76	1	1	10.88	6.7	1.72	4.65E-03
Q8IUS5	Epoxide hydrolase 4 OS=Homo sapiens GN=EPHX4 PE=2 SV=2 - [EPHX4_HUMAN]	12.98	4	4	42.30	8.4	1.72	5.01E-03
P35232	Prohibitin OS=Homo sapiens GN=PHB PE=1 SV=1 - [PHB_HUMAN]	76.47	1	171	29.79	5.8	1.72	7.36E-03
A0A172Q3A0	Fibroblast activation protein (Fragment) OS=Homo sapiens PE=2 SV=1 - [A0A172Q3A0_HUMAN]	4.77	3	3	84.82	6.6	1.72	8.96E-04
E5RIK9	Transcription termination factor 3, mitochondrial OS=Homo sapiens GN=MTERF3 PE=1 SV=1 - [E5RIK9_HUMAN]	12.47	5	5	41.54	9.1	1.72	1.71E-02
Q9HD23	Magnesium transporter MRS2 homolog, mitochondrial OS=Homo sapiens GN=MRS2 PE=1 SV=1 - [MRS2_HUMAN]	14	5	7	50.29	6.9	1.72	2.01E-03

Q09LL4	CD40 (Fragment) OS=Homo sapiens PE=4 SV=1 - [Q09LL4_HUMAN]	7.69	1	1	13.15	5.2	1.72	2.05E-03
B3KTD5	cDNA FLJ38083 fis, clone CTONG2016408, highly similar to UBX domain-containing protein 2 OS=Homo sapiens PE=2 SV=1 - [B3KTD5_HUMAN]	10.95	3	5	31.26	9.5	1.72	1.58E-03
Q13433	Zinc transporter ZIP6 OS=Homo sapiens GN=SLC39A6 PE=1 SV=3 - [S39A6_HUMAN]	1.19	1	1	84.99	6.9	1.72	1.83E-03
B2R921	cDNA, FLJ94171, highly similar to Homo sapiens solute carrier family 25 (mitochondrial carrier; ornithine transporter) member 15 (SLC25A15), nuclear gene encoding mitochondrial protein, mRNA OS=Homo sapiens PE=2	29.24	9	22	32.77	9.3	1.72	6.18E-03
Q9NRK6	ATP-binding cassette sub-family B member 10, mitochondrial OS=Homo sapiens GN=ABCB10 PE=1 SV=2 - [ABCBA_HUMAN]	24.53	16	31	79.10	9.8	1.72	2.88E-03
I3L0K6	A-kinase anchor protein 1, mitochondrial (Fragment) OS=Homo sapiens GN=AKAP1 PE=1 SV=1 - [I3L0K6_HUMAN]	24.09	2	2	15.08	7.4	1.72	6.76E-03
A0A087WUQ6	Glutathione peroxidase OS=Homo sapiens GN=GPX1 PE=1 SV=1 - [A0A087WUQ6_HUMAN]	61.88	10	38	21.92	6.6	1.73	6.75E-03
P28288	ATP-binding cassette sub-family D member 3 OS=Homo sapiens GN=ABCD3 PE=1 SV=1 - [ABCD3_HUMAN]	19.88	12	41	75.43	9.4	1.73	3.97E-03
Q9Y4L1	Hypoxia up-regulated protein 1 OS=Homo sapiens GN=HYOU1 PE=1 SV=1 - [HYOU1_HUMAN]	50.05	39	137	111.27	5.2	1.73	7.07E-04
Q15797	Mothers against decapentaplegic homolog 1 OS=Homo sapiens GN=SMAD1 PE=1 SV=1 - [SMAD1_HUMAN]	3.01	1	1	52.23	7.3	1.73	3.47E-02
B4E2Z3	cDNA FLJ54090, highly similar to 4F2 cell-surface antigen heavy chain OS=Homo sapiens PE=2 SV=1 - [B4E2Z3_HUMAN]	25.24	11	26	55.90	5.2	1.73	1.00E-02

H0YBQ8	Disintegrin and metalloproteinase domain-containing protein 28 (Fragment) OS=Homo sapiens GN=ADAM28 PE=1 SV=1 - [H0YBQ8_HUMAN]	4.6	1	1	19.33	8.0	1.74	2.67E-02
Q9Y3D2	Methionine-R-sulfoxide reductase B2, mitochondrial OS=Homo sapiens GN=MSRB2 PE=1 SV=2 - [MSRB2_HUMAN]	12.64	2	2	19.52	8.6	1.74	1.08E-03
A0A087X1K9	Acyl-protein thioesterase 1 OS=Homo sapiens GN=LYPLA1 PE=1 SV=1 - [A0A087X1K9_HUMAN]	34.94	4	7	17.97	5.1	1.74	3.40E-03
Q96BR5	Cytochrome c oxidase assembly factor 7 OS=Homo sapiens GN=COA7 PE=1 SV=2 - [COA7_HUMAN]	22.08	5	8	25.69	6.0	1.74	3.01E-03
P54886	Delta-1-pyrroline-5-carboxylate synthase OS=Homo sapiens GN=ALDH18A1 PE=1 SV=2 - [P5CS_HUMAN]	51.07	33	72	87.25	7.1	1.74	3.42E-03
Q9NSE4	Isoleucine--tRNA ligase, mitochondrial OS=Homo sapiens GN=IARS2 PE=1 SV=2 - [SYIM_HUMAN]	42.39	37	149	113.72	7.2	1.74	8.90E-03
Q53GR7	Solute carrier family 25, member 13 (Citrin) variant (Fragment) OS=Homo sapiens PE=2 SV=1 - [Q53GR7_HUMAN]	50.37	20	93	74.07	8.4	1.74	3.20E-03
O43837	Isocitrate dehydrogenase [NAD] subunit beta, mitochondrial OS=Homo sapiens GN=IDH3B PE=1 SV=2 - [IDH3B_HUMAN]	41.56	13	43	42.16	8.5	1.75	8.69E-03
K7EIE9	Tyrosine-protein phosphatase non-receptor type 2 (Fragment) OS=Homo sapiens GN=PTPN2 PE=1 SV=1 - [K7EIE9_HUMAN]	7.28	1	1	17.74	9.7	1.75	4.56E-02
H7C285	Tetraspanin-15 (Fragment) OS=Homo sapiens GN=TSPAN15 PE=1 SV=1 - [H7C285_HUMAN]	12.5	2	5	18.21	5.1	1.75	1.94E-03
Q7L0Y3	Mitochondrial ribonuclease P protein 1 OS=Homo sapiens GN=TRMT10C PE=1 SV=2 - [MRRP1_HUMAN]	55.58	23	67	47.32	9.4	1.75	2.37E-03

Q5T0Y8	Acid sphingomyelinase-like phosphodiesterase 3b (Fragment) OS=Homo sapiens GN=SMPDL3B PE=1 SV=1 - [Q5T0Y8_HUMAN]	8.55	1	1	29.51	4.9	1.76	3.09E-03
P27701	CD82 antigen OS=Homo sapiens GN=CD82 PE=1 SV=1 - [CD82_HUMAN]	8.99	2	3	29.61	5.2	1.76	9.40E-03
Q53GL1	Nicotinamide-nucleotide adenylyltransferase (Fragment) OS=Homo sapiens PE=2 SV=1 - [Q53GL1_HUMAN]	21.86	5	9	31.90	8.9	1.76	3.90E-03
A0A0S2Z5Q3	Sulfatase modifying factor 2 isoform 2 OS=Homo sapiens GN=SUMF2 PE=2 SV=1 - [A0A0S2Z5Q3_HUMAN]	37.87	3	15	30.81	6.9	1.76	1.55E-03
F8VV56	CD63 antigen OS=Homo sapiens GN=CD63 PE=1 SV=1 - [F8VV56_HUMAN]	20.69	3	10	16.01	7.9	1.76	7.95E-03
O95470	Sphingosine-1-phosphate lyase 1 OS=Homo sapiens GN=SGPL1 PE=1 SV=3 - [SGPL1_HUMAN]	33.98	17	31	63.48	9.2	1.76	8.30E-03
B7Z7P4	cDNA FLJ53627, highly similar to Antigen peptide transporter 1 OS=Homo sapiens PE=2 SV=1 - [B7Z7P4_HUMAN]	30.71	10	15	59.90	5.6	1.76	5.54E-03
Q9BQP7	Mitochondrial genome maintenance exonuclease 1 OS=Homo sapiens GN=MGME1 PE=1 SV=1 - [MGME1_HUMAN]	30.23	9	17	39.40	7.7	1.76	1.07E-02
Q9UGM3	Deleted in malignant brain tumors 1 protein OS=Homo sapiens GN=DMBT1 PE=1 SV=2 - [DMBT1_HUMAN]	6.96	1	1	260.57	5.4	1.77	7.63E-03
Q8WY22	BRI3-binding protein OS=Homo sapiens GN=BRI3BP PE=1 SV=1 - [BRI3B_HUMAN]	6.77	1	2	27.82	9.4	1.77	1.11E-02
B4DLN1	Uncharacterized protein OS=Homo sapiens PE=2 SV=1 - [B4DLN1_HUMAN]	24.43	3	30	48.07	9.5	1.77	1.22E-02

P26447	Protein S100-A4 OS=Homo sapiens GN=S100A4 PE=1 SV=1 - [S10A4_HUMAN]	35.64	4	8	11.72	6.1	1.77	1.44E-02
O15427	Monocarboxylate transporter 4 OS=Homo sapiens GN=SLC16A3 PE=1 SV=1 - [MOT4_HUMAN]	9.89	4	7	49.44	8.0	1.77	7.41E-03
K7EM09	Transmembrane protein 205 (Fragment) OS=Homo sapiens GN=TMEM205 PE=1 SV=1 - [K7EM09_HUMAN]	25	2	10	13.38	8.1	1.77	7.04E-03
P11021	78 kDa glucose-regulated protein OS=Homo sapiens GN=HSPA5 PE=1 SV=2 - [GRP78_HUMAN]	56.73	35	376	72.29	5.2	1.77	1.87E-04
H0YDQ1	Probable dolichyl pyrophosphate Glc1Man9GlcNAc2 alpha-1,3-glucosyltransferase (Fragment) OS=Homo sapiens GN=ALG8 PE=1 SV=1 - [H0YDQ1_HUMAN]	10.34	1	2	9.78	10.1	1.77	4.84E-03
P27144	Adenylate kinase 4, mitochondrial OS=Homo sapiens GN=AK4 PE=1 SV=1 - [KAD4_HUMAN]	57.4	11	36	25.25	8.4	1.78	5.67E-03
E5RH34	CMP-N-acetylneuraminate-beta-galactosamide-alpha-2,3-sialyltransferase 1 (Fragment) OS=Homo sapiens GN=ST3GAL1 PE=1 SV=1 - [E5RH34_HUMAN]	15.28	1	1	8.50	10.4	1.78	7.72E-03
Q9Y3D9	28S ribosomal protein S23, mitochondrial OS=Homo sapiens GN=MRPS23 PE=1 SV=2 - [RT23_HUMAN]	40.53	7	24	21.76	8.9	1.78	1.06E-03
H9LP39	Cytochrome c oxidase subunit 1 OS=Homo sapiens GN=COX1 PE=3 SV=1 - [H9LP39_HUMAN]	6.04	1	1	57.01	6.7	1.79	2.44E-03
O43295	SLIT-ROBO Rho GTPase-activating protein 3 OS=Homo sapiens GN=SRGAP3 PE=1 SV=3 - [SRGP3_HUMAN]	0.82	1	1	124.43	6.7	1.79	1.32E-02
Q6P161	39S ribosomal protein L54, mitochondrial OS=Homo sapiens GN=MRPL54 PE=1 SV=1 - [RM54_HUMAN]	48.55	3	6	15.81	9.6	1.79	8.11E-03

A0A059QB80	ATP synthase subunit a OS=Homo sapiens GN=ATP6 PE=4 SV=1 - [A0A059QB80_HUMAN]	4.42	1	17	24.74	10.1	1.79	6.63E-03
Q8N4H5	Mitochondrial import receptor subunit TOM5 homolog OS=Homo sapiens GN=TOMM5 PE=1 SV=1 - [TOM5_HUMAN]	27.45	2	4	6.03	9.7	1.79	6.95E-03
Q9NX62	Inositol monophosphatase 3 OS=Homo sapiens GN=IMPAD1 PE=1 SV=1 - [IMPA3_HUMAN]	8.36	4	4	38.66	6.9	1.80	2.36E-02
P28838	Cytosol aminopeptidase OS=Homo sapiens GN=LAP3 PE=1 SV=3 - [AMPL_HUMAN]	63.97	27	84	56.13	7.9	1.80	2.08E-03
A8K651	cDNA FLJ75700, highly similar to Homo sapiens complement component 1, q subcomponent binding protein (C1QBP), nuclear gene encoding mitochondrial protein, mRNA OS=Homo sapiens PE=2 SV=1 - [A8K651_HUMAN]	31.56	6	125	31.36	4.8	1.80	4.10E-03
P68402	Platelet-activating factor acetylhydrolase IB subunit beta OS=Homo sapiens GN=PAFAH1B2 PE=1 SV=1 - [PA1B2_HUMAN]	12.23	2	3	25.55	5.9	1.80	3.25E-02
H0YHH1	Uncharacterized protein (Fragment) OS=Homo sapiens PE=4 SV=1 - [H0YHH1_HUMAN]	9.01	1	1	12.17	7.3	1.80	5.35E-03
E9PE17	28S ribosomal protein S17, mitochondrial (Fragment) OS=Homo sapiens GN=MRPS17 PE=1 SV=1 - [E9PE17_HUMAN]	51.94	4	10	14.36	9.8	1.81	1.52E-03
Q6DN12	Multiple C2 and transmembrane domain-containing protein 2 OS=Homo sapiens GN=MCTP2 PE=1 SV=3 - [MCTP2_HUMAN]	1.37	1	1	99.53	7.6	1.81	1.78E-04
P13667	Protein disulfide-isomerase A4 OS=Homo sapiens GN=PDIA4 PE=1 SV=2 - [PDIA4_HUMAN]	61.71	42	268	72.89	5.1	1.81	4.52E-03
P30040	Endoplasmic reticulum resident protein 29 OS=Homo sapiens GN=ERP29 PE=1 SV=4 - [ERP29_HUMAN]	47.89	13	62	28.98	7.3	1.81	5.45E-03

K7ER12	UPF0449 protein C19orf25 (Fragment) OS=Homo sapiens GN=C19orf25 PE=1 SV=2 - [K7ER12_HUMAN]	47.62	1	1	4.51	6.6	1.82	8.72E-03
B4DEK8	Tetraspanin OS=Homo sapiens PE=2 SV=1 - [B4DEK8_HUMAN]	6.51	2	3	23.97	7.2	1.82	3.56E-03
A0A0G2JMH6	HLA class II histocompatibility antigen, DR alpha chain OS=Homo sapiens GN=HLA-DRA PE=1 SV=1 - [A0A0G2JMH6_HUMAN]	32.68	2	47	28.60	5.0	1.82	8.26E-04
L0R8F8	Alternative protein SMCR7L OS=Homo sapiens GN=SMCR7L PE=3 SV=1 - [L0R8F8_HUMAN]	11.43	1	1	8.44	10.6	1.82	3.23E-03
Q8TD06	Anterior gradient protein 3 OS=Homo sapiens GN=AGR3 PE=1 SV=1 - [AGR3_HUMAN]	45.18	6	11	19.16	8.0	1.82	6.74E-03
Q8NF37	Lysophosphatidylcholine acyltransferase 1 OS=Homo sapiens GN=LPCAT1 PE=1 SV=2 - [PCAT1_HUMAN]	12.36	6	7	59.11	6.0	1.82	2.36E-03
B4DT82	cDNA FLJ50662, highly similar to Solute carrier family 2, facilitated glucosetransporter member 5 OS=Homo sapiens PE=2 SV=1 - [B4DT82_HUMAN]	3.11	1	1	42.26	6.4	1.82	7.40E-03
O43715	TP53-regulated inhibitor of apoptosis 1 OS=Homo sapiens GN=TRIAP1 PE=1 SV=1 - [TRIA1_HUMAN]	17.11	1	1	8.78	5.5	1.82	4.70E-04
A0A0K0KSD4	MHC class I antigen (Fragment) OS=Homo sapiens GN=HLA-C PE=3 SV=1 - [A0A0K0KSD4_HUMAN]	48.62	1	13	21.22	7.9	1.82	5.58E-03
Q96RQ9	L-amino-acid oxidase OS=Homo sapiens GN=IL4I1 PE=1 SV=1 - [OXLA_HUMAN]	13.76	6	13	62.84	8.7	1.82	8.40E-03
V9HW35	Epididymis secretory protein Li 55 OS=Homo sapiens GN=HEL-S-55 PE=2 SV=1 - [V9HW35_HUMAN]	59.26	8	58	17.02	7.2	1.83	4.19E-03

A0A024R755	Calumenin, isoform CRA_a OS=Homo sapiens GN=CALU PE=4 SV=1 - [A0A024R755_HUMAN]	43.17	3	35	37.11	4.6	1.83	3.50E-03
Q9Y6X5	Bis(5'-adenosyl)-triphosphatase ENPP4 OS=Homo sapiens GN=ENPP4 PE=1 SV=3 - [ENPP4_HUMAN]	4.64	2	2	51.61	6.2	1.83	2.89E-03
P49448	Glutamate dehydrogenase 2, mitochondrial OS=Homo sapiens GN=GLUD2 PE=1 SV=2 - [DHE4_HUMAN]	38.89	3	81	61.40	8.5	1.83	1.38E-03
Q9P1F3	Costars family protein ABRACL OS=Homo sapiens GN=ABRACL PE=1 SV=1 - [ABRAL_HUMAN]	16.05	1	1	9.05	6.3	1.83	4.65E-03
P05141	ADP/ATP translocase 2 OS=Homo sapiens GN=SLC25A5 PE=1 SV=7 - [ADT2_HUMAN]	49.66	6	304	32.83	9.7	1.84	1.21E-03
N1NSB5	MHC class II antigen (Fragment) OS=Homo sapiens GN=HLA-DRB1 PE=4 SV=1 - [N1NSB5_HUMAN]	52.81	2	10	10.89	6.5	1.84	1.55E-03
G5E9P5	39S ribosomal protein L52, mitochondrial OS=Homo sapiens GN=MRPL52 PE=1 SV=1 - [G5E9P5_HUMAN]	30	1	1	11.67	9.5	1.84	2.19E-02
A8K4A8	cDNA FLJ76156, highly similar to Homo sapiens aspartyl-tRNA synthetase 2 (DARS2), mRNA OS=Homo sapiens PE=2 SV=1 - [A8K4A8_HUMAN]	33.33	16	47	73.53	7.8	1.84	6.40E-03
M0QXC5	Napsin-A (Fragment) OS=Homo sapiens GN=NAPSA PE=1 SV=1 - [M0QXC5_HUMAN]	2.61	1	1	29.09	6.4	1.84	8.08E-04
Q96ES6	Major facilitator superfamily domain-containing protein 3 OS=Homo sapiens GN=MFSD3 PE=2 SV=1 - [MFSD3_HUMAN]	2.18	1	1	42.67	9.2	1.84	4.98E-03
B4DPP0	cDNA FLJ51032, highly similar to CD9 antigen OS=Homo sapiens PE=2 SV=1 - [B4DPP0_HUMAN]	17.02	3	11	15.90	6.7	1.84	1.92E-04

E5RJK7	LYR motif-containing protein 2 OS=Homo sapiens GN=LYRM2 PE=1 SV=1 - [E5RJK7_HUMAN]	11.11	1	1	8.64	11.2	1.85	3.85E-03
B4DLK5	cDNA FLJ51600, highly similar to Cytochrome P450 1B1 (EC 1.14.14.1) OS=Homo sapiens PE=2 SV=1 - [B4DLK5_HUMAN]	28.4	11	17	46.68	7.1	1.85	3.24E-04
Q9BUP3	Oxidoreductase HTATIP2 OS=Homo sapiens GN=HTATIP2 PE=1 SV=2 - [HTAI2_HUMAN]	30.58	6	9	27.03	8.4	1.85	3.72E-04
F8VZY9	Keratin, type I cytoskeletal 18 OS=Homo sapiens GN=KRT18 PE=1 SV=1 - [F8VZY9_HUMAN]	37.6	13	23	43.75	5.4	1.86	7.05E-03
O15320	cTAGE family member 5 OS=Homo sapiens GN=CTAGE5 PE=1 SV=4 - [CTGE5_HUMAN]	21.14	13	22	90.94	5.2	1.86	1.35E-02
Q6UX53	Methyltransferase-like protein 7B OS=Homo sapiens GN=METTL7B PE=1 SV=2 - [MET7B_HUMAN]	18.03	4	5	27.76	8.4	1.86	7.92E-03
Q9BS26	Endoplasmic reticulum resident protein 44 OS=Homo sapiens GN=ERP44 PE=1 SV=1 - [ERP44_HUMAN]	30.05	11	25	46.94	5.3	1.86	3.62E-04
P61604	10 kDa heat shock protein, mitochondrial OS=Homo sapiens GN=HSPE1 PE=1 SV=2 - [CH10_HUMAN]	83.33	10	140	10.92	8.9	1.86	3.10E-03
Q9BQ13	BTB/POZ domain-containing protein KCTD14 OS=Homo sapiens GN=KCTD14 PE=1 SV=2 - [KCD14_HUMAN]	9.41	2	3	29.57	8.6	1.87	1.11E-02
B2R7I0	cDNA, FLJ93451, highly similar to Homo sapiens thioredoxin domain containing 14 (TXNDC14), mRNA OS=Homo sapiens PE=2 SV=1 - [B2R7I0_HUMAN]	22.97	7	9	34.00	8.7	1.87	1.55E-05
H7BZJ3	Protein disulfide-isomerase A3 (Fragment) OS=Homo sapiens GN=PDIA3 PE=1 SV=1 - [H7BZJ3_HUMAN]	74.8	1	58	13.51	7.3	1.87	1.35E-03

O14548	Cytochrome c oxidase subunit 7A-related protein, mitochondrial OS=Homo sapiens GN=COX7A2L PE=1 SV=2 - [COX7R_HUMAN]	54.39	4	8	12.61	9.4	1.88	5.33E-03
Q14257	Reticulocalbin-2 OS=Homo sapiens GN=RCN2 PE=1 SV=1 - [RCN2_HUMAN]	35.33	7	23	36.85	4.4	1.88	2.53E-04
B2R7D7	cDNA, FLJ93394, highly similar to Homo sapiens anterior gradient 2 homolog (Xenopus laevis) (AGR2), mRNA OS=Homo sapiens PE=2 SV=1 - [B2R7D7_HUMAN]	33.71	5	12	19.99	9.0	1.88	4.31E-04
Q59F54	Solute carrier family 2 (Facilitated glucose transporter), member 3 variant (Fragment) OS=Homo sapiens PE=2 SV=1 - [Q59F54_HUMAN]	3.11	1	1	31.85	5.9	1.88	7.41E-04
Q96RP9	Elongation factor G, mitochondrial OS=Homo sapiens GN=GFM1 PE=1 SV=2 - [EFGM_HUMAN]	36.22	25	56	83.42	7.0	1.88	9.21E-04
Q9Y5U9	Immediate early response 3-interacting protein 1 OS=Homo sapiens GN=IER3IP1 PE=1 SV=1 - [IR3IP_HUMAN]	24.39	1	1	8.96	8.2	1.88	5.31E-04
H9E7B8	Cytochrome c oxidase subunit 2 (Fragment) OS=Homo sapiens GN=COX2 PE=3 SV=1 - [H9E7B8_HUMAN]	27.27	1	65	24.84	4.7	1.88	1.97E-02
B4DKE6	cDNA FLJ60629, highly similar to Replication factor C subunit 3 OS=Homo sapiens PE=2 SV=1 - [B4DKE6_HUMAN]	5.9	1	1	34.81	8.6	1.89	1.26E-03
U6FVB0	Tyrosine-protein kinase receptor OS=Homo sapiens GN=CD74-Ntrk1 fusion gene PE=2 SV=1 - [U6FVB0_HUMAN]	13.88	9	21	70.29	8.1	1.89	3.00E-03
P50443	Sulfate transporter OS=Homo sapiens GN=SLC26A2 PE=1 SV=2 - [S26A2_HUMAN]	2.57	2	2	81.61	8.4	1.89	4.48E-02
B2R6X6	Peptidyl-prolyl cis-trans isomerase OS=Homo sapiens PE=2 SV=1 - [B2R6X6_HUMAN]	39.13	6	28	22.03	9.5	1.89	7.39E-05

P78310	Coxsackievirus and adenovirus receptor OS=Homo sapiens GN=CXADR PE=1 SV=1 - [CXAR_HUMAN]	11.78	4	4	40.00	7.6	1.90	6.11E-03
Q5TC12	ATP synthase mitochondrial F1 complex assembly factor 1 OS=Homo sapiens GN=ATPAF1 PE=1 SV=1 - [ATPF1_HUMAN]	29.57	8	17	36.41	8.0	1.90	9.90E-03
D6REQ6	Ribonuclease T2 OS=Homo sapiens GN=RNASET2 PE=1 SV=1 - [D6REQ6_HUMAN]	18.81	3	5	25.29	6.1	1.90	1.28E-02
Q92685	Dol-P-Man:Man(5)GlcNAc(2)-PP-Dol alpha-1,3-mannosyltransferase OS=Homo sapiens GN=ALG3 PE=1 SV=1 - [ALG3_HUMAN]	9.36	4	6	50.09	9.4	1.91	3.91E-03
P59665	Neutrophil defensin 1 OS=Homo sapiens GN=DEFA1 PE=1 SV=1 - [DEF1_HUMAN]	19.15	2	14	10.19	7.0	1.91	4.30E-02
Q4G148	Glucoside xylosyltransferase 1 OS=Homo sapiens GN=GXYLT1 PE=1 SV=2 - [GXLT1_HUMAN]	8.18	3	5	50.53	8.6	1.92	4.44E-04
Q86VC1	XPO4 protein (Fragment) OS=Homo sapiens GN=XPO4 PE=2 SV=1 - [Q86VC1_HUMAN]	2.9	1	2	93.77	4.8	1.92	1.42E-02
P54687	Branched-chain-amino-acid aminotransferase, cytosolic OS=Homo sapiens GN=BCAT1 PE=1 SV=3 - [BCAT1_HUMAN]	5.44	2	2	42.94	5.3	1.92	1.96E-03
P58107	Epiplakin OS=Homo sapiens GN=EPPK1 PE=1 SV=2 - [EPIPL_HUMAN]	8.39	2	13	555.28	5.6	1.93	1.62E-03
O60613	15 kDa selenoprotein OS=Homo sapiens GN=SEP15 PE=1 SV=3 - [SEP15_HUMAN]	19.75	3	8	17.78	5.0	1.93	1.12E-03
O95881	Thioredoxin domain-containing protein 12 OS=Homo sapiens GN=TXNDC12 PE=1 SV=1 - [TXD12_HUMAN]	31.4	5	24	19.19	5.4	1.93	6.49E-04

A0A172Q3A8	Folate receptor (Fragment) OS=Homo sapiens PE=2 SV=1 - [A0A172Q3A8_HUMAN]	17.87	4	15	24.27	7.8	1.93	3.23E-03
A0PJ86	ACPP protein (Fragment) OS=Homo sapiens GN=ACPP PE=2 SV=1 - [A0PJ86_HUMAN]	16.92	4	6	30.34	9.2	1.94	1.02E-02
P06702	Protein S100-A9 OS=Homo sapiens GN=S100A9 PE=1 SV=1 - [S10A9_HUMAN]	74.56	7	24	13.23	6.1	1.94	2.77E-03
Q8WUY1	Protein THEM6 OS=Homo sapiens GN=THEM6 PE=1 SV=2 - [THEM6_HUMAN]	41.83	8	15	23.85	9.6	1.94	6.92E-03
P15924	Desmoplakin OS=Homo sapiens GN=DSP PE=1 SV=3 - [DESP_HUMAN]	29.36	75	124	331.57	6.8	1.94	7.87E-04
Q9H488	GDP-fucose protein O-fucosyltransferase 1 OS=Homo sapiens GN=POFUT1 PE=1 SV=1 - [OFUT1_HUMAN]	26.55	7	16	43.93	8.5	1.94	3.20E-05
Q8NBK3	Sulfatase-modifying factor 1 OS=Homo sapiens GN=SUMF1 PE=1 SV=3 - [SUMF1_HUMAN]	12.3	4	5	40.53	6.7	1.94	2.60E-03
B1ALH6	Phytanoyl-CoA dioxygenase, peroxisomal OS=Homo sapiens GN=PHYH PE=1 SV=1 - [B1ALH6_HUMAN]	22.74	8	12	36.94	8.2	1.94	7.71E-03
P36222	Chitinase-3-like protein 1 OS=Homo sapiens GN=CHI3L1 PE=1 SV=2 - [CH3L1_HUMAN]	20.63	7	9	42.60	8.5	1.94	7.51E-04
Q05CP8	CCDC6 protein (Fragment) OS=Homo sapiens GN=CCDC6 PE=2 SV=1 - [Q05CP8_HUMAN]	14.67	4	4	38.14	9.4	1.95	5.92E-04
Q9UBH6	Xenotropic and polytropic retrovirus receptor 1 OS=Homo sapiens GN=XPR1 PE=1 SV=1 - [XPR1_HUMAN]	9.91	7	11	81.48	8.4	1.95	5.51E-03

A0A0S2Z580	Ethylmalonic encephalopathy 1 isoform 2 (Fragment) OS=Homo sapiens GN=ETHE1 PE=2 SV=1 - [A0A0S2Z580_HUMAN]	27.98	5	15	26.63	6.8	1.95	3.34E-03
Q15084	Protein disulfide-isomerase A6 OS=Homo sapiens GN=PDIA6 PE=1 SV=1 - [PDIA6_HUMAN]	58.18	18	91	48.09	5.1	1.96	7.42E-03
A8K335	cDNA FLJ76254, highly similar to Homo sapiens gamma-glutamyl hydrolase (GGH), mRNA OS=Homo sapiens PE=2 SV=1 - [A8K335_HUMAN]	25.79	7	12	36.02	7.4	1.97	1.43E-02
Q53G71	Calreticulin variant (Fragment) OS=Homo sapiens PE=2 SV=1 - [Q53G71_HUMAN]	62.32	21	156	46.89	4.4	1.98	1.19E-02
Q9NY97	N-acetyllactosaminide beta-1,3-N-acetylglucosaminyltransferase 2 OS=Homo sapiens GN=B3GNT2 PE=1 SV=2 - [B3GN2_HUMAN]	2.27	1	1	45.99	8.5	1.98	2.76E-02
O75381	Peroxisomal membrane protein PEX14 OS=Homo sapiens GN=PEX14 PE=1 SV=1 - [PEX14_HUMAN]	14.85	5	7	41.21	4.9	1.99	6.51E-04
P17096	High mobility group protein HMG-I/HMG-Y OS=Homo sapiens GN=HMGA1 PE=1 SV=3 - [HMGA1_HUMAN]	23.36	2	3	11.67	10.3	1.99	3.92E-03
Q6PI41	AURKAIP1 protein (Fragment) OS=Homo sapiens GN=AURKAIP1 PE=2 SV=1 - [Q6PI41_HUMAN]	6.17	1	3	18.65	10.5	1.99	8.96E-04
Q8TAA5	GrpE protein homolog 2, mitochondrial OS=Homo sapiens GN=GRPEL2 PE=1 SV=1 - [GRPE2_HUMAN]	7.56	2	3	25.42	7.7	1.99	1.89E-03
P10696	Alkaline phosphatase, placental-like OS=Homo sapiens GN=ALPPL2 PE=2 SV=4 - [PPBN_HUMAN]	30.08	2	20	57.34	6.3	2.01	5.16E-03
B6ECB2	MUC1 isoform J23 OS=Homo sapiens GN=MUC1 PE=2 SV=1 - [B6ECB2_HUMAN]	21.67	3	10	19.63	6.4	2.01	5.30E-05

A0A0S2C388	MHC class II antigen (Fragment) OS=Homo sapiens GN=HLA-DPB1 PE=3 SV=1 - [A0A0S2C388_HUMAN]	25.4	5	7	28.68	7.4	2.01	1.13E-02
P05109	Protein S100-A8 OS=Homo sapiens GN=S100A8 PE=1 SV=1 - [S10A8_HUMAN]	39.78	5	9	10.83	7.0	2.01	1.21E-02
P48735	Isocitrate dehydrogenase [NADP], mitochondrial OS=Homo sapiens GN=IDH2 PE=1 SV=2 - [IDHP_HUMAN]	56.64	27	355	50.88	8.7	2.02	2.07E-03
A0A075B746	28S ribosomal protein S21, mitochondrial OS=Homo sapiens GN=MRPS21 PE=1 SV=1 - [A0A075B746_HUMAN]	39.08	3	6	10.68	9.9	2.02	3.18E-03
C7DJS2	Glutathione S-transferase pi (Fragment) OS=Homo sapiens GN=GSTP1 PE=2 SV=1 - [C7DJS2_HUMAN]	42.38	1	14	16.66	5.1	2.02	3.78E-02
Q8TCD1	UPF0729 protein C18orf32 OS=Homo sapiens GN=C18orf32 PE=3 SV=1 - [CR032_HUMAN]	23.68	2	2	8.66	9.1	2.02	2.49E-02
O19697	Hla-dcalpha partial alpha 2 domain (extra cellular domain) (Fragment) OS=Homo sapiens PE=4 SV=1 - [O19697_HUMAN]	90.63	1	11	3.84	5.1	2.03	2.45E-03
Q86YS6	Ras-related protein Rab-43 OS=Homo sapiens GN=RAB43 PE=1 SV=1 - [RAB43_HUMAN]	24.06	3	7	23.32	5.6	2.03	2.07E-03
K7ELD9	Synaptogyrin-2 OS=Homo sapiens GN=SYNGR2 PE=1 SV=1 - [K7ELD9_HUMAN]	12.31	1	1	6.74	9.4	2.04	1.02E-04
I3L3M4	Uncharacterized protein OS=Homo sapiens PE=4 SV=1 - [I3L3M4_HUMAN]	20.48	1	1	8.83	4.8	2.05	2.84E-03
Q8IZV5	Retinol dehydrogenase 10 OS=Homo sapiens GN=RDH10 PE=1 SV=1 - [RDH10_HUMAN]	12.32	4	7	38.06	7.4	2.05	2.12E-03

A0A087WYU6	Lysophosphatidic acid phosphatase type 6 (Fragment) OS=Homo sapiens GN=ACP6 PE=1 SV=1 - [A0A087WYU6_HUMAN]	28.92	3	4	18.94	7.3	2.05	1.77E-03
P23786	Carnitine O-palmitoyltransferase 2, mitochondrial OS=Homo sapiens GN=CPT2 PE=1 SV=2 - [CPT2_HUMAN]	42.4	5	77	73.73	8.2	2.05	1.99E-02
Q05BS6	UNC93B1 protein (Fragment) OS=Homo sapiens GN=UNC93B1 PE=2 SV=1 - [Q05BS6_HUMAN]	6.53	3	5	56.51	7.8	2.05	2.07E-03
S4R329	ER membrane protein complex subunit 4 OS=Homo sapiens GN=EMC4 PE=1 SV=1 - [S4R329_HUMAN]	40.28	2	3	7.80	9.8	2.06	8.58E-04
G3V5T0	Maleylacetoacetate isomerase OS=Homo sapiens GN=GSTZ1 PE=1 SV=1 - [G3V5T0_HUMAN]	28.71	1	7	22.60	7.7	2.07	8.20E-04
Q5VT66	Mitochondrial amidoxime-reducing component 1 OS=Homo sapiens GN=MARC1 PE=1 SV=1 - [MARC1_HUMAN]	23.44	5	10	37.48	8.9	2.07	3.49E-04
D3DX03	Amine oxidase OS=Homo sapiens GN=ABP1 PE=3 SV=1 - [D3DX03_HUMAN]	18.16	9	11	82.68	7.2	2.08	1.44E-03
B4DH65	cDNA FLJ53323, highly similar to 2-oxoglutarate dehydrogenase E1 component, mitochondrial (EC 1.2.4.2) OS=Homo sapiens PE=2 SV=1 - [B4DH65_HUMAN]	45.09	1	124	97.63	6.8	2.08	2.19E-03
Q8WYJ5	Protein kinase C inhibitor-2 OS=Homo sapiens PE=2 SV=1 - [Q8WYJ5_HUMAN]	46.88	4	16	13.91	7.0	2.09	2.49E-04
P51589	Cytochrome P450 2J2 OS=Homo sapiens GN=CYP2J2 PE=1 SV=2 - [CP2J2_HUMAN]	12.35	5	6	57.57	8.6	2.09	6.55E-03
P13498	Cytochrome b-245 light chain OS=Homo sapiens GN=CYBA PE=1 SV=3 - [CY24A_HUMAN]	11.28	2	3	21.00	9.5	2.10	9.04E-04

P60468	Protein transport protein Sec61 subunit beta OS=Homo sapiens GN=SEC61B PE=1 SV=2 - [SC61B_HUMAN]	44.79	4	8	9.97	11.6	2.11	6.78E-04
A0A0C4DGJ8	Electron transfer flavoprotein regulatory factor 1 OS=Homo sapiens GN=ETFRF1 PE=1 SV=1 - [A0A0C4DGJ8_HUMAN]	30.68	3	5	10.60	9.9	2.12	2.01E-03
Q56VL3	OCIA domain-containing protein 2 OS=Homo sapiens GN=OCIAD2 PE=1 SV=1 - [OCAD2_HUMAN]	48.7	8	20	16.94	9.0	2.12	1.22E-04
B4DV41	cDNA FLJ50879, highly similar to Cytochrome P450 4B1 (EC 1.14.14.1) OS=Homo sapiens PE=2 SV=1 - [B4DV41_HUMAN]	13.76	2	4	25.75	6.2	2.13	2.44E-03
Q9Y2Q3	Glutathione S-transferase kappa 1 OS=Homo sapiens GN=GSTK1 PE=1 SV=3 - [GSTK1_HUMAN]	66.37	13	129	25.48	8.4	2.13	4.24E-07
Q8IZF6	Adhesion G-protein coupled receptor G4 OS=Homo sapiens GN=ADGRG4 PE=2 SV=2 - [AGRG4_HUMAN]	0.23	1	1	333.16	6.2	2.13	1.40E-03
F8W1I4	La-related protein 4 (Fragment) OS=Homo sapiens GN=LARP4 PE=1 SV=1 - [F8W1I4_HUMAN]	8	1	1	14.13	4.4	2.15	2.19E-02
B4DHJ7	cDNA FLJ60537, highly similar to BCL2/adenovirus E1B 19 kDa protein-interacting protein 3 OS=Homo sapiens PE=2 SV=1 - [B4DHJ7_HUMAN]	12.22	1	2	19.93	6.3	2.15	2.03E-03
Q96AG4	Leucine-rich repeat-containing protein 59 OS=Homo sapiens GN=LRRC59 PE=1 SV=1 - [LRC59_HUMAN]	46.58	15	47	34.91	9.6	2.16	3.51E-03
H3BSA6	dCTP pyrophosphatase 1 OS=Homo sapiens GN=DCTPP1 PE=1 SV=1 - [H3BSA6_HUMAN]	14.08	1	2	7.65	8.0	2.16	2.53E-03
Q9NRP4	Succinate dehydrogenase assembly factor 3, mitochondrial OS=Homo sapiens GN=SDHAF3 PE=1 SV=1 - [SDHF3_HUMAN]	16	2	2	14.64	9.1	2.17	8.93E-04

P54868	Hydroxymethylglutaryl-CoA synthase, mitochondrial OS=Homo sapiens GN=HMGCS2 PE=1 SV=1 - [HMCS2_HUMAN]	12.8	7	11	56.60	8.2	2.17	2.10E-03
B3KVA7	cDNA FLJ16309 fis, clone SKMUS2007816, highly similar to Creatine kinase, sarcomeric mitochondrial (EC 2.7.3.2) OS=Homo sapiens PE=2 SV=1 - [B3KVA7_HUMAN]	35.67	8	24	40.45	7.4	2.17	7.81E-03
T2D2V0	MHC class II antigen (Fragment) OS=Homo sapiens GN=HLA-DQB PE=4 SV=1 - [T2D2V0_HUMAN]	18.89	2	4	10.71	8.0	2.18	1.02E-03
Q16822	Phosphoenolpyruvate carboxykinase [GTP], mitochondrial OS=Homo sapiens GN=PCK2 PE=1 SV=3 - [PCKGM_HUMAN]	35	1	31	70.68	7.6	2.18	4.48E-03
D4IH33	MHC class I antigen (Fragment) OS=Homo sapiens GN=HLA-Cw PE=3 SV=1 - [D4IH33_HUMAN]	43.09	1	17	21.06	7.3	2.19	2.72E-03
Q99470	Stromal cell-derived factor 2 OS=Homo sapiens GN=SDF2 PE=1 SV=2 - [SDF2_HUMAN]	9.95	2	3	23.01	7.3	2.19	5.71E-04
P23219	Prostaglandin G/H synthase 1 OS=Homo sapiens GN=PTGS1 PE=1 SV=2 - [PGH1_HUMAN]	22.87	12	22	68.64	7.2	2.20	2.92E-04
Q8NFV4	Protein ABHD11 OS=Homo sapiens GN=ABHD11 PE=1 SV=1 - [ABHDB_HUMAN]	48.89	11	45	34.67	9.5	2.20	4.29E-04
E3SWK8	MHC class I antigen (Fragment) OS=Homo sapiens GN=HLA-C PE=3 SV=1 - [E3SWK8_HUMAN]	53.04	1	15	21.12	7.4	2.21	2.77E-02
Q9HC07	Transmembrane protein 165 OS=Homo sapiens GN=TMEM165 PE=1 SV=1 - [TM165_HUMAN]	21.3	3	5	34.88	7.0	2.22	1.23E-02
P05787	Keratin, type II cytoskeletal 8 OS=Homo sapiens GN=KRT8 PE=1 SV=7 - [K2C8_HUMAN]	48.86	21	73	53.67	5.6	2.23	2.73E-03

P23378	Glycine dehydrogenase (decarboxylating), mitochondrial OS=Homo sapiens GN=GLDC PE=1 SV=2 - [GCSP_HUMAN]	30	21	44	112.66	7.1	2.24	2.09E-03
Q96SQ9	Cytochrome P450 2S1 OS=Homo sapiens GN=CYP2S1 PE=1 SV=2 - [CP2S1_HUMAN]	17.26	6	9	55.78	8.6	2.24	1.56E-02
A0A0U1RR89	BAG family molecular chaperone regulator 1 (Fragment) OS=Homo sapiens GN=BAG1 PE=1 SV=1 - [A0A0U1RR89_HUMAN]	15.87	1	1	7.23	8.2	2.25	8.42E-03
P16422	Epithelial cell adhesion molecule OS=Homo sapiens GN=EPCAM PE=1 SV=2 - [EPCAM_HUMAN]	24.84	5	7	34.91	7.5	2.25	7.53E-03
A0A087WZU5	Tetraspanin-6 OS=Homo sapiens GN=TSPAN6 PE=1 SV=1 - [A0A087WZU5_HUMAN]	24.82	3	4	15.60	6.3	2.25	1.33E-02
Q9P270	SLAIN motif-containing protein 2 OS=Homo sapiens GN=SLAIN2 PE=1 SV=2 - [SLAI2_HUMAN]	1.03	1	1	62.51	9.5	2.27	7.50E-04
O15235	28S ribosomal protein S12, mitochondrial OS=Homo sapiens GN=MRPS12 PE=1 SV=1 - [RT12_HUMAN]	5.8	1	1	15.16	10.3	2.27	1.21E-03
Q9Y446	Plakophilin-3 OS=Homo sapiens GN=PKP3 PE=1 SV=1 - [PKP3_HUMAN]	3.76	2	2	87.03	9.3	2.27	4.28E-03
H0YBX3	Protein RIDA (Fragment) OS=Homo sapiens GN=RIDA PE=1 SV=1 - [H0YBX3_HUMAN]	31.5	3	4	13.82	9.3	2.27	2.36E-03
Q9NYJ1	Cytochrome c oxidase assembly factor 4 homolog, mitochondrial OS=Homo sapiens GN=COA4 PE=1 SV=2 - [COA4_HUMAN]	27.59	2	2	10.13	6.0	2.28	8.18E-03
Q9HCB6	Spondin-1 OS=Homo sapiens GN=SPON1 PE=1 SV=2 - [SPON1_HUMAN]	34.08	21	38	90.91	6.1	2.28	5.63E-04

A0A0B5E3E9	Sodium-dependent phosphate transport protein 2B (Fragment) OS=Homo sapiens GN=SLC34A2 PE=2 SV=1 - [A0A0B5E3E9_HUMAN]	6.24	4	6	75.55	8.2	2.29	2.90E-04
Q96C01	Protein FAM136A OS=Homo sapiens GN=FAM136A PE=1 SV=1 - [F136A_HUMAN]	31.16	4	10	15.63	7.6	2.30	3.06E-04
R4GNF9	Thiosulfate sulfurtransferase/rhodanese-like domain-containing protein 1 OS=Homo sapiens GN=TSTD1 PE=1 SV=1 - [R4GNF9_HUMAN]	50	3	5	7.05	7.2	2.31	4.38E-04
P08729	Keratin, type II cytoskeletal 7 OS=Homo sapiens GN=KRT7 PE=1 SV=5 - [K2C7_HUMAN]	27.72	11	34	51.35	5.5	2.32	2.84E-04
H0YMJ4	Kinesin-like protein KIF23 OS=Homo sapiens GN=KIF23 PE=1 SV=2 - [H0YMJ4_HUMAN]	16.51	1	2	12.36	9.3	2.34	3.26E-03
Q92876	Kallikrein-6 OS=Homo sapiens GN=KLK6 PE=1 SV=1 - [KLK6_HUMAN]	4.51	1	1	26.84	7.4	2.36	1.13E-02
P56277	Cx9C motif-containing protein 4 OS=Homo sapiens GN=CMC4 PE=1 SV=1 - [CMC4_HUMAN]	25	1	1	7.74	8.2	2.36	7.17E-03
Q9NXQ7	cDNA FLJ20106 fis, clone COL04830 OS=Homo sapiens PE=2 SV=1 - [Q9NXQ7_HUMAN]	3.66	1	1	31.76	9.3	2.37	4.32E-03
P50591	Tumor necrosis factor ligand superfamily member 10 OS=Homo sapiens GN=TNFSF10 PE=1 SV=1 - [TNF10_HUMAN]	4.27	1	1	32.49	7.4	2.38	9.73E-03
Q16880	2-hydroxyacylsphingosine 1-beta-galactosyltransferase OS=Homo sapiens GN=UGT8 PE=2 SV=2 - [CGT_HUMAN]	5.91	3	3	61.40	9.5	2.38	1.06E-02
Q9NXW2	DnaJ homolog subfamily B member 12 OS=Homo sapiens GN=DNAJB12 PE=1 SV=4 - [DJB12_HUMAN]	7.47	3	3	41.79	8.5	2.40	3.07E-03

P09758	Tumor-associated calcium signal transducer 2 OS=Homo sapiens GN=TACSTD2 PE=1 SV=3 - [TACD2_HUMAN]	26.63	7	10	35.69	8.9	2.41	1.12E-03
C9JQ41	Coiled-coil domain-containing protein 58 OS=Homo sapiens GN=CCDC58 PE=1 SV=1 - [C9JQ41_HUMAN]	27.69	3	7	15.29	8.9	2.42	1.40E-03
C9JFR7	Cytochrome c (Fragment) OS=Homo sapiens GN=CYCS PE=1 SV=1 - [C9JFR7_HUMAN]	57.43	6	42	11.33	9.7	2.43	1.11E-03
P29373	Cellular retinoic acid-binding protein 2 OS=Homo sapiens GN=CRABP2 PE=1 SV=2 - [RABP2_HUMAN]	23.91	3	5	15.68	5.4	2.46	6.13E-04
P55145	Mesencephalic astrocyte-derived neurotrophic factor OS=Homo sapiens GN=MANF PE=1 SV=3 - [MANF_HUMAN]	42.31	8	23	20.69	8.7	2.48	2.87E-03
Q6P1M0	Long-chain fatty acid transport protein 4 OS=Homo sapiens GN=SLC27A4 PE=1 SV=1 - [S27A4_HUMAN]	11.66	5	19	72.02	8.5	2.51	8.46E-03
A5Y5A3	PC1/MRPS28 fusion protein OS=Homo sapiens PE=2 SV=1 - [A5Y5A3_HUMAN]	12.95	2	6	31.16	6.2	2.52	7.25E-03
Q9UBX3	Mitochondrial dicarboxylate carrier OS=Homo sapiens GN=SLC25A10 PE=1 SV=2 - [DIC_HUMAN]	34.49	1	12	31.26	9.5	2.54	1.06E-02
Q96CP7	Calfacilitin OS=Homo sapiens GN=TLCD1 PE=2 SV=1 - [TLCD1_HUMAN]	3.24	1	1	28.53	9.5	2.57	6.85E-03
Q5JTJ3	Cytochrome c oxidase assembly factor 6 homolog OS=Homo sapiens GN=COA6 PE=1 SV=1 - [COA6_HUMAN]	32	3	6	14.11	8.3	2.59	4.68E-03
P82932	28S ribosomal protein S6, mitochondrial OS=Homo sapiens GN=MRPS6 PE=1 SV=3 - [RT06_HUMAN]	39.2	5	11	14.22	9.3	2.59	1.33E-03

H3BTQ8	Kunitz-type protease inhibitor 1 (Fragment) OS=Homo sapiens GN=SPINT1 PE=1 SV=8 - [H3BTQ8_HUMAN]	2.66	1	1	33.15	6.2	2.63	2.42E-03
F8WAX7	Selenoprotein K OS=Homo sapiens GN=SELK PE=1 SV=1 - [F8WAX7_HUMAN]	12.5	1	1	8.29	9.8	2.64	4.78E-02
B3KY44	Solute carrier family 11 (Proton-coupled divalent metal ion transporters), member 2, isoform CRA_c OS=Homo sapiens GN=SLC11A2 PE=2 SV=1 - [B3KY44_HUMAN]	2.68	1	1	44.86	7.6	2.67	4.89E-02
D0W033	MHC class I antigen (Fragment) OS=Homo sapiens GN=HLA-A PE=3 SV=1 - [D0W033_HUMAN]	37.73	1	12	31.57	5.9	2.69	4.15E-03
P09110	3-ketoacyl-CoA thiolase, peroxisomal OS=Homo sapiens GN=ACAA1 PE=1 SV=2 - [THIK_HUMAN]	46.23	4	50	44.26	8.4	2.70	1.00E-02
H7C4E5	Cytochrome c oxidase copper chaperone (Fragment) OS=Homo sapiens GN=COX17 PE=1 SV=1 - [H7C4E5_HUMAN]	12.07	1	1	6.41	7.7	2.75	2.33E-03
Q8NDA2	Hemicentin-2 OS=Homo sapiens GN=HMCN2 PE=2 SV=3 - [HMCN2_HUMAN]	0.51	1	1	541.64	5.9	2.77	2.44E-02
J3KQF5	Cytosolic carboxypeptidase 4 OS=Homo sapiens GN=AGBL1 PE=1 SV=2 - [J3KQF5_HUMAN]	2.23	1	1	116.11	7.1	2.80	2.39E-02
Q05639	Elongation factor 1-alpha 2 OS=Homo sapiens GN=EEF1A2 PE=1 SV=1 - [EF1A2_HUMAN]	23.76	2	46	50.44	9.0	2.83	3.67E-04
Q9BTP0	Protein Wnt (Fragment) OS=Homo sapiens PE=2 SV=1 - [Q9BTP0_HUMAN]	2.73	1	1	32.33	9.0	2.84	4.53E-03
Q7Z7D3	V-set domain-containing T-cell activation inhibitor 1 OS=Homo sapiens GN=VTCN1 PE=1 SV=1 - [VTCN1_HUMAN]	6.74	2	3	30.86	5.3	2.84	3.10E-03

A0A090N8E5	Similar to OG-2 homeodomain protein-like similar to U65067 (PID:g1575526) OS=Homo sapiens GN=LOC135935 PE=4 SV=1 - [A0A090N8E5_HUMAN]	6.33	1	1	52.71	7.9	2.90	6.88E-03
A0A1B0GTN5	Protein LOC101929926 (Fragment) OS=Homo sapiens GN=LOC101929926 PE=4 SV=1 - [A0A1B0GTN5_HUMAN]	13.1	1	2	9.31	11.5	2.91	1.30E-02
A0A140VJV6	Testicular tissue protein Li 183 OS=Homo sapiens PE=2 SV=1 - [A0A140VJV6_HUMAN]	13.85	1	4	21.81	8.4	3.29	5.81E-04
R9RV30	Androgen-induced 1/Golgi SNAP receptor complex member 1 variant 1 fusion protein (Fragment) OS=Homo sapiens GN=AIG1 PE=2 SV=1 - [R9RV30_HUMAN]	11.25	1	3	9.00	8.9	3.33	2.47E-02
A8MZH8	Pituitary tumor-transforming gene 1 protein-interacting protein OS=Homo sapiens GN=PTTG1IP PE=1 SV=1 - [A8MZH8_HUMAN]	23.36	2	5	11.59	8.1	3.37	3.70E-03
Q9HCY8	Protein S100-A14 OS=Homo sapiens GN=S100A14 PE=1 SV=1 - [S10AE_HUMAN]	27.88	3	6	11.65	5.2	3.38	1.70E-03
Q9BSE5	Agmatinase, mitochondrial OS=Homo sapiens GN=AGMAT PE=1 SV=2 - [SPEB_HUMAN]	11.36	2	3	37.64	7.6	3.40	7.42E-04
Q9C002	Normal mucosa of esophagus-specific gene 1 protein OS=Homo sapiens GN=NMES1 PE=2 SV=1 - [NMES1_HUMAN]	51.81	4	10	9.61	9.5	3.46	3.35E-04
O60635	Tetraspanin-1 OS=Homo sapiens GN=TSPAN1 PE=1 SV=2 - [TSN1_HUMAN]	5.39	1	1	26.28	5.2	3.47	2.43E-03
H0YN27	Protein-glutamine gamma-glutamyltransferase K (Fragment) OS=Homo sapiens GN=TGM1 PE=1 SV=1 - [H0YN27_HUMAN]	9.63	1	1	14.48	7.2	3.49	7.37E-05
Q67AU1	MHC class II antigen (Fragment) OS=Homo sapiens GN=HLA-DPA1 PE=4 SV=1 - [Q67AU1_HUMAN]	31.18	1	4	10.84	5.1	3.58	6.74E-03

H7C0U6	Uncharacterized protein C1orf53 (Fragment) OS=Homo sapiens GN=C1orf53 PE=1 SV=1 - [H7C0U6_HUMAN]	10.98	1	1	8.78	8.6	3.72	3.35E-03
A0A0K0KRA3	MHC class I antigen (Fragment) OS=Homo sapiens GN=HLA-A PE=3 SV=1 - [A0A0K0KRA3_HUMAN]	47.51	1	11	21.19	7.0	4.12	5.31E-04
